# A scalable and operationally simple radical trifluoromethylation

**DOI:** 10.1038/ncomms8919

**Published:** 2015-08-10

**Authors:** Joel W. Beatty, James J. Douglas, Kevin P. Cole, Corey R. J. Stephenson

**Affiliations:** 1 Department of Chemistry, University of Michigan, Ann Arbor, Michigan 48109, USA; 2 Small Molecule Design and Development, Lilly Research Laboratories, Eli Lilly and Company, Indianapolis, Indiana 46285, USA

## Abstract

The large number of reagents that have been developed for the synthesis of trifluoromethylated compounds is a testament to the importance of the CF_3_ group as well as the associated synthetic challenge. Current state-of-the-art reagents for appending the CF_3_ functionality directly are highly effective; however, their use on preparative scale has minimal precedent because they require multistep synthesis for their preparation, and/or are prohibitively expensive for large-scale application. For a scalable trifluoromethylation methodology, trifluoroacetic acid and its anhydride represent an attractive solution in terms of cost and availability; however, because of the exceedingly high oxidation potential of trifluoroacetate, previous endeavours to use this material as a CF_3_ source have required the use of highly forcing conditions. Here we report a strategy for the use of trifluoroacetic anhydride for a scalable and operationally simple trifluoromethylation reaction using pyridine *N*-oxide and photoredox catalysis to affect a facile decarboxylation to the CF_3_ radical.

## Abstract (web summary)

Trifluoromethylation is a key transformation, particularly for pharmaceuticals, but many reagents are expensive and difficult to scale up. Here, the authors show that trifluoroacetic anhydride can act as a CF_3_ source, allowing the radical reactions to be easily and inexpensively carried out at scale.

## Graphical abstract

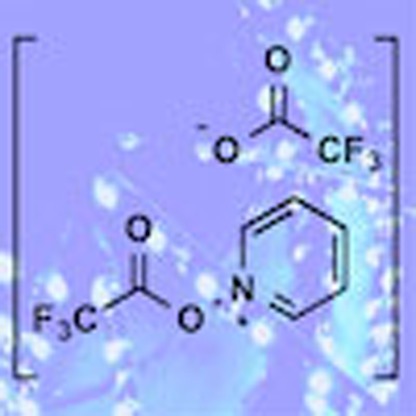

The incorporation of fluorine into drug molecules can dramatically improve metabolic stability and other pharmacokinetic properties^1^, a phenomenon reflected by the presence of fluorine in a large proportion of recent drug candidates^2^. Technologies for introducing fluorine and the trifluoromethyl (CF_3_) group have become increasingly available to medicinal chemists for the synthesis and development of lead compounds^3^,^4^. Currently, a number of effective methodologies utilizing radical^3^,^5^,^6^,^7^,^8^,^9^, nucleophilic^3^,^10^,^11^,^12^,^13^,^14^ and electrophilic^3^,^15^,^16^ CF_3_ sources are available for arene and heteroarene trifluoromethylation. The development of requisite methodologies for trifluoromethylation on preparative scale has become an increasingly important, yet challenging priority. Compounding this issue, many reagents most commonly used for laboratory-scale trifluoromethylation are prohibitively expensive and/or unobtainable in the quantities required for scale-up (for a cost comparison, see Supplementary Note 1)^17^. As a consequence, advancements in scalable, inexpensive and operationally simple protocols for fluorine incorporation are of the utmost importance for modern pharmaceutical^2^, agrochemical^18^ and specialty chemical production^19^.

To this end, we report a strategy for the use of trifluoroacetic anhydride for a scalable and operationally simple trifluoromethylation reaction using pyridine *N*-oxide and photoredox catalysis to effect decarboxylation to the CF_3_ radical. The reaction proceeds on a range of vinyl, aryl and heteroaryl substrates and has been demonstrated on a significant scale in both batch (100 g) and flow (20 g).

## Results

### Reaction design

**Figure 1:**
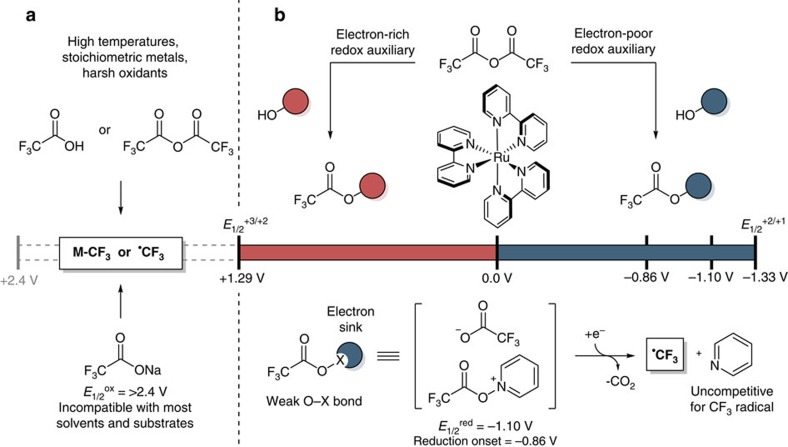
Design principles for a mild photochemical decarboxylation of trifluoroacetate. (**a**) TFA and TFAA are desirable CF_3_ sources from the perspective of availability and cost, but have traditionally proven challenging to utilize. (**b**) Strategic electrochemical tuning using the sacrificial redox auxiliary pyridine *N*-oxide allows for reductive decarboxylation within the electrochemical window of tris(bipyridine)ruthenium(II). The reagent undergoes irreversible reduction (determined with differential pulse voltammetry, *E*_1/2_^red^=−1.10 V versus SCE in MeCN, Supplementary Figs 1 and 2) with onset reduction observable at −0.86 V versus SCE.

Trifluoroacetic acid (TFA) and its anhydride (TFAA) represent attractive sources of CF_3_ because of their ready availability and inexpensive nature (TFAA $35 per kg at 1,000 kg) but have seen minimal application towards this end. This dearth of methods is a direct result of the challenge associated with the decarboxylation of TFA (Fig. 1a)—a process that occurs in a polar manner only at high temperatures (>140 °C for copper trifluoroacetate)^20^,^21^. Alternative methods avoiding these high temperatures employ strongly oxidizing conditions (stoichiometric silver^22^, XeF_2_ (ref. 23) or peroxide^24^) to access the carboxylate radical, which then extrudes CO_2_ to produce the CF_3_ radical^25^. Because of the exceedingly high oxidation potential of the TFA anion (F_3_CCO_2_Na *E*_1/2_^ox^>+2.4 V versus SCE (saturated calomel electrode))^26^, direct electrochemical methods are limited in scope, as these forcing potentials are strong enough to oxidize most organic solvents.

To address this challenge, we envisioned that a mild decarboxylation of TFAA could be accomplished through appending a sacrificial redox auxiliary to alter the requisite electrochemical potentials (Fig. 1b)^27^. As part of the implementation of this design strategy, we took into account aspects of Oda and Okadas' studies on the use of *N*-(acyloxy)phthalimides to facilitate decarboxylation^28^, as well as insights from Barton on the use of thiohydroxamic acid derivatives as photocleavable auxiliaries^29^. In this regard, the use of an electron-rich auxiliary would enable the oxidation of the TFAA adduct at less-forcing potentials; however, because of electronic matching effects, the resultant electrophilic CF_3_ radical would be highly likely to recombine with the cleaved auxiliary^30^. The alternative reduction of an electron-poor auxiliary presents a solution to this problem, as the use of an electron-poor reagent would fail to out-compete more electron-rich substrates for CF_3_ radical sequestration.

We identified pyridine *N*-oxide as the reagent of choice for the formation of a reducible TFAA adduct (Fig. 1b). The reagent combination is effective in equal stoichiometry with respect to the substrate, and is sufficiently inexpensive for large-scale implementation (pyridine *N*-oxide $40–$70 per kg at 1,000 kg). Furthermore, following cleavage of the weak N–O bond and CO_2_ extrusion, the generation of pyridine as a byproduct is a highly beneficial design feature, preventing the need for exogenous base and avoiding competitive reagent functionalization. The adduct exhibits reduction at mild potentials (*E*_1/2_^red^=−1.10 V versus SCE) with an observable reduction onset at −0.86 V versus SCE. The use of a photoactive redox catalyst such as tris(bipyridine)ruthenium(II) chloride^31^ (Ru(bpy)_3_Cl_2_) was seen as ideal, as its broad electrochemical window encompasses the reduction potential of the *N*-oxide/TFAA adduct (Fig. 1b), and should provide feasible scalability through redox-neutral catalytic operation^32^. Stern–Volmer quenching studies suggest that the adduct is effective in oxidatively quenching the catalyst’s excited state (see Supplementary Fig. 3); however, it should be noted that the measured onset reduction of the reagent is more negative than the calculated half wave reduction potential of the Ru(bpy)_3_^2+*^ excited state (*E*_1/2_*^II/III^=−0.81 V)^32^.

### Trifluoromethylation of vinyl, aryl and heteroaryl substrates

**Figure 2:**
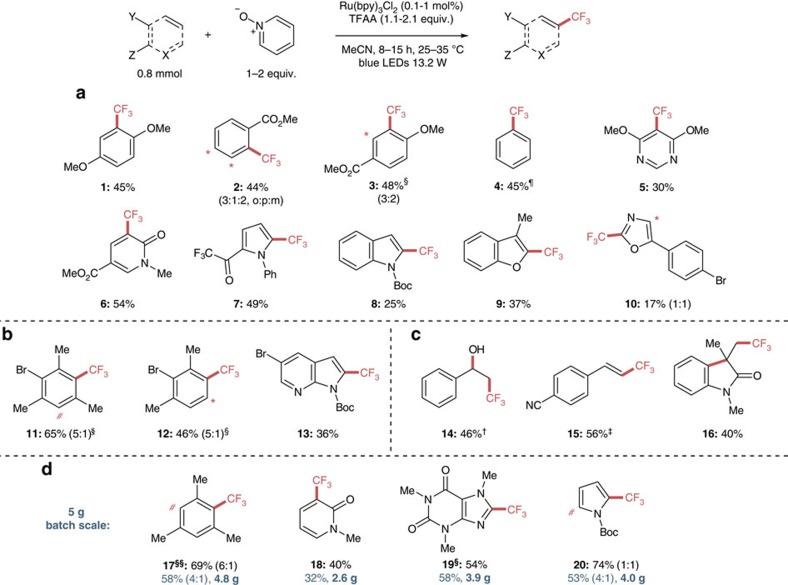
Scalable radical trifluoromethylation of arenes, heteroarenes and alkenes with trifluoroacetic anhydride. (**a**) A variety of electron-rich and electron-neutral substrates are amenable to trifluoromethylation. (**b**) Substrates with functionality for cross-coupling were compatible with the reaction conditions. (**c**) Radical trifluoromethylation of alkenes. (**d**) Products **17**–**20** were obtained from reactions run on 5 g scale using 0.1 mol% Ru(bpy)_3_Cl_2_. Isolated yields are indicated below each entry, except in the case of volatile compounds (^19^F NMR yields) and represent a single experiment. See Supplementary Methods for experimental details. *Position of functionalization on the minor regioisomeric product. ¶10 equiv. benzene. #Position of functionalization on the minor doubly functionalized product. §4 equiv. of pyridine *N*-oxide and 8 equiv. of TFAA, 24 h. †Run in CH_2_Cl_2_, stirred with methanol on reaction completion. ‡Run in CH_2_Cl_2_, stirred with DBU on reaction completion. §§3 equiv. of pyridine *N*-oxide and 3.1 equiv. of TFAA. Ru(bpy)_3_Cl_2_, tris(bipyridine)ruthenium(II) chloride; TFAA, trifluoroacetic anhydride; MeCN, acetonitrile; Me, methyl; Boc, *tert*-butyloxycarbonyl; Ph, phenyl; Ts, *para*-toluenesulfonyl.

Trifluoromethylation reactions were performed in acetonitrile, with 1–2 equivalents of pyridine *N*-oxide providing generally optimal results (Fig. 2, for optimization details see Supplementary Table 1). Importantly, the reaction was found to be tolerant of air and adventitious moisture, resulting in a practical reaction set-up without the need for dry solvent, pre-dried reagents (pyridine *N*-oxide is hygroscopic), or degassing of the reaction mixture. Arenes containing both electron-donating and mildly electron-withdrawing groups could be trifluoromethylated in moderate yields (Fig. 2a, **1**–**3**), and benzene could be trifluoromethylated in 45% yield (**4**). A variety of heterocycles could also be functionalized (**5**–**10**), and aryl bromides were found to be well tolerated by the reaction conditions (Fig. 2b). In addition, atom transfer reactivity was accessible on a number of substrates (Fig. 2c) with effective control for hydrolysis (**14**), elimination (**15**) and cyclization (**16**) of the CF_3_ addition products.

Free NH and OH groups are unsurprisingly acylated in the presence of TFAA, resulting in a decrease in substrate electron density and corresponding reactivity. For example, *N*-Boc-aniline is readily acylated, and the resulting *N*-acyl carbamate is not trifluoromethylated under the reaction conditions. In addition, certain electron-rich substrates undergo Friedel-Crafts reactivity in the presence of TFAA, including 5-(4-bromophenyl)oxazole (precursor to **10**, partial acylation), and *N*-phenyl pyrrole. In the case of *N*-phenyl pyrrole, the use of an additional equivalent of TFAA results in formation of **7** in one pot. Under optimized conditions, as shown in Fig. 2, minimal quantities of trifluoromethylated pyridine derivatives were observed (≤5%); however, small amounts of trifluoromethylated byproducts could be observed with increasing equivalents of pyridine *N*-oxide. While there are many reported methodologies employing the radical addition of CF_3_ to pyridine derivatives, we observe a high preference for substrate functionalization, validating a critical aspect of our reaction design. Consistent with these observations, electron-poor substrates including pyridines displayed minimal reactivity (see Supplementary Fig. 4). Substrate C–H functionalization during the reaction results in the generation of acid, which is inherently buffered by the concomitant formation of free pyridine. This buffering effect also results in a more pronounced difference between the electron density of the substrate and that of pyridinium trifluoroacetate. On reaction completion, these polar products of reagent consumption could be easily removed, either through direct filtration of the reaction mixture or through aqueous workup.

The simplicity of operation and availability of reagents combined to allow us to demonstrate the reaction on preparative laboratory scale. Conducting the reaction on 5 g of material in commonly available laboratory glassware employing 0.1 mol% Ru(bpy)_3_Cl_2_, we observed only a moderate reduction in isolated yield (Fig. 2d, **17**–**20**) realizing our goal of scalability, and demonstrating the potential application of this methodology.

**Figure 3:**
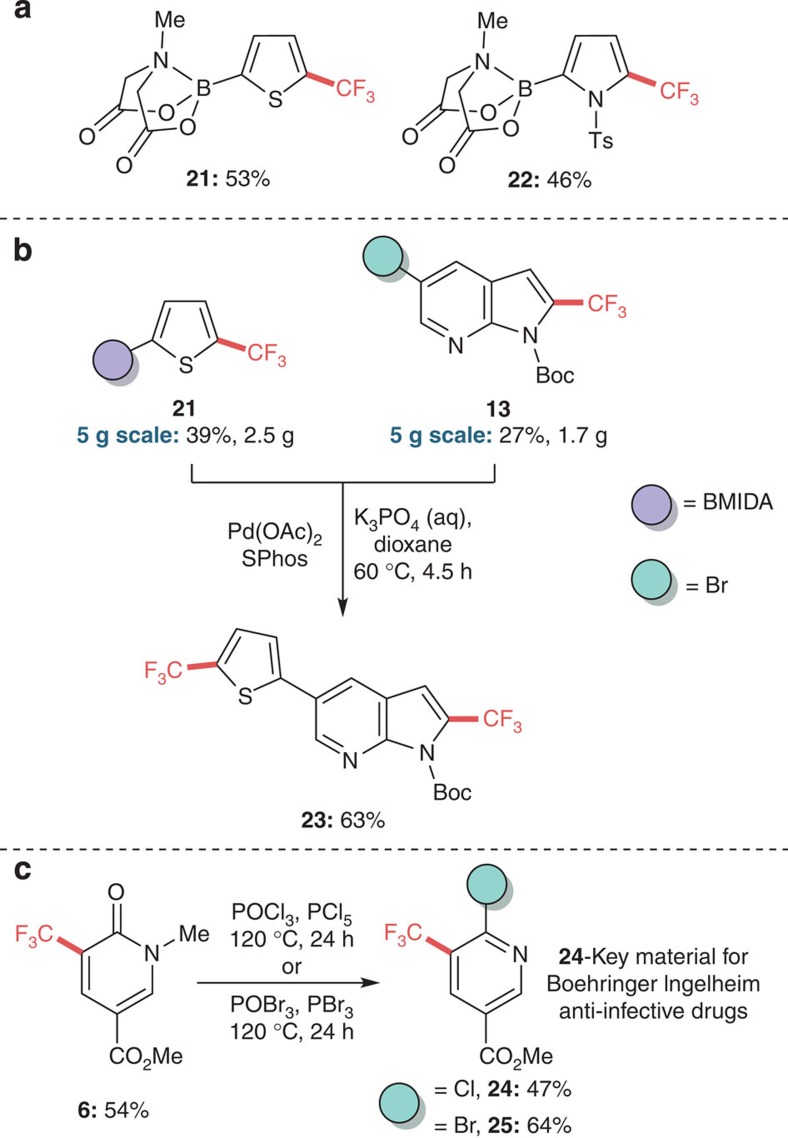
Synthetic utility of the trifluoromethylation procedure. (**a**) Trifluoromethylation of cross-coupling precursors. (**b**) Trifluoromethylation followed by cross-coupling leads to the production of specialty scaffolds. (**c**) Synthesis of 3-(trifluoromethyl)pyridine derivatives can be accomplished through pyridone functionalization. Boc, *tert*-butyloxycarbonyl; SPhos, 2-dicyclohexylphosphino-2′,6′-dimethoxybiphenyl; BMIDA, boronic acid methyliminodiacetic acid ester. Yields represent a single experiment.

Preparative scale reactions on materials containing the appropriate handle to allow further functionalization also proceeded successfully. Methyliminodiacetic acid ester (MIDA) boronates **21** and **22** could be readily obtained via the trifluoromethylation conditions, demonstrating the stability of the MIDA functionality to these radical conditions (Fig. 3a). Thiophene **21** was produced on a 5-g scale and cross-coupled with azaindole **13** to provide the highly functionalized doubly trifluoromethylated compound **23** (Fig. 3b). This trifluoromethylation/cross-coupling strategy represents an attractive tool to rapidly access a wide range of CF_3_-containing scaffolds, with potential application to the construction of compound libraries used in drug discovery. This type of approach is a complementary strategy to late-stage trifluoromethylation, by which functional-group diversity is generated from a single advanced intermediate^5^.

### Application to the synthesis of CF_3_ pyridines

While the methodology is not currently applicable to direct the formation of medicinally relevant pyridine substrates, 2-halo-3-trifluoromethylated pyridine motifs can be accessed via further transformation of *N*-methyl-2-pyridone analogues such as **6** (Fig. 3c). 2-Halo-3-trifluoromethylated pyridines are useful building blocks that can serve as versatile precursors to drug-like compounds; for example, pyridine **24** represents a key starting material for anti-infective drugs currently in development by Boehringer Inglehiem^17^. The current route to kilogram quantities of **24** was developed with an emphasis on cost, limiting reagent choice (see Supplementary Note 1), as ‘the large-scale availability of CF_3_SiMe_3_ and higher alkyl variants is still limited and their cost can be prohibitive for use in commercial pharmaceutical manufacture’^17^. We viewed this substrate as an ideal test of the strategic use of our trifluoromethylation procedure. Accordingly, *N*-methyl pyridone **6** was synthesized in 54% yield from the corresponding pyridone then converted to pyridine **24** (47% yield unoptimized) to provide the desired compound and demonstrate an alternative synthesis utilizing TFAA.

### Reactions at large scale and in flow

**Figure 4:**
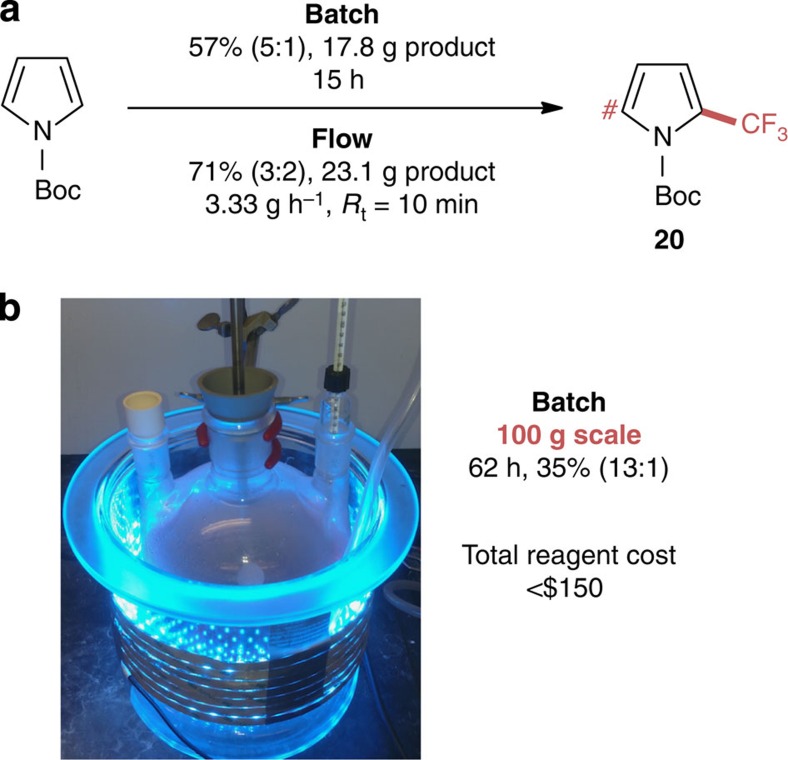
Scalable trifluoromethylation of *N*-Boc-pyrrole in batch and flow. (**a**) An 18.3-g reaction in batch provides 17.8 g of product (57%) in 15 h. Improved yields were obtained when the reaction was run at a steady state in flow, with a 71% yield of isolated material from 20 g of starting material. (**b**) The reaction can be run on 100 g scale for <$150 total reagent and catalyst cost calculated using academic vendor pricing. #Position of functionalization on the minor doubly functionalized product. Yields represent a single experiment. Boc, *tert*-butyloxycarbonyl.

Owing to the minimal cost of the reagent and low catalyst loadings, we began to assess the viability of this reaction on process scale (Fig. 4). A reaction conducted using 18 g of *N*-Boc-pyrrole provided a comparable yield to that obtained on small scale (17.8 g 57%). Furthermore, a promising yield of 35% was obtained on 100 g of material; the reaction time was notably longer (62 versus 15 h), a feature that can be attributed to a reduction in the relative light intensity in the larger vessel as we chose not to fully optimize the light source at this time (see Supplementary Methods). These initial results indicate that the method is potentially applicable to large-scale batch production; however, another attractive option for scale up involves the use of continuous processing. The benefits from conducting photochemical reactions in flow have been previously demonstrated^33^,^34^, and predominantly originate from more efficient light irradiation of the reaction solution. A preliminary study found improved results (23.1 g 71%) for a 20-g reaction performed in flow with a residence time of 10 min (10 ml reactor volume), demonstrating the viability of this procedure in the context of continuous processing (Supplementary Fig. 5). Efforts towards further batch scale up combined with optimization of the continuous flow conditions are ongoing and will be reported in due course.

## Discussion

The direct trifluoromethylation of arenes and heteroarenes circumvents more traditional multistep procedures for CF_3_ arene production, most notably the radical chlorination of aryl methyl groups followed by high-pressure treatment in hydrofluoric acid^35^. This current industrial standard has been demonstrated to be highly effective; however, alternative functional-group tolerant procedures for direct trifluoromethylation remain an important priority. In this regard, further work to utilize inexpensive and atom-economical CF_3_ sources such as HCF_3_ or TFA should be prioritized. The challenge of utilizing TFAA as a CF_3_ source is reflected by moderate yields for the reported transformation; nevertheless, this work represents a significant breakthrough that will allow for further understanding of this reactivity, with the aim of developing a cost-effective large-scale route to specialty trifluoromethylated chemicals. Finally, while trifluoromethylation has demonstrated its synthetic value in the pharmaceutical, agrochemical and fine chemical industries, radical perfluoroalkylation has found widespread use in the synthesis of specialty materials and in fluorous tagging^36^. Owing to the availability of various perfluoroalkyl anhydrides, we believe that our method may find use in this regard, or furthermore as a general means of accessing radical species from carboxylic acids that are challenging to oxidize via traditional means.

## Methods

### General trifluoromethylation procedure

To a 2-dram vial equipped with a stir bar was added pyridine *N*-oxide (76 mg, 0.80 mmol, 1.0 equiv), Ru(bpy)_3_Cl_2_·6H_2_O (6.0 mg, 1.0 mol%) and substrate (0.80 mmol). The combined materials were then dissolved in MeCN (2.0 ml) and stirred to form a homogeneous solution. Trifluoroacetic anhydride (120 μl, 180 mg, 0.88 mmol, 1.1 equiv) was then added to the resulting solution. The vial was equipped with a screw-on cap with septum, and a 25-gauge needle was placed through the septum for the duration of the reaction. Three 4.4 W LED light strips (positioned 2.5 cm away) were turned on and the reaction was allowed to run for 12–15 h before the light source was removed. Workup was performed by diluting the reaction with CH_2_Cl_2_ and washing with 1 N HCl, followed by saturated NaHCO_3_ and then brine. The organic layer was dried over sodium sulfate before filtering and concentrating at 40 °C under reduced pressure. For NMR analysis of the compounds in this article, see Supplementary Figs 6–46.

## Additional information

**How to cite this article:** Beatty, J. W. *et al*. A scalable and operationally simple radical trifluoromethylation. *Nat. Commun.* 6:7919 doi: 10.1038/ncomms8919 (2015).

## Supplementary Material

Supplementary Information Supplementary Figures 1-46, Supplementary Table 1, Supplementary Note 1, Supplementary Methods and Supplementary References
