# Supplementary material for: A scalable and operationally simple radical trifluoromethylation
Source: Nat Commun. 2015 Aug 10;6:7919. doi: 10.1038/ncomms8919 (PMC4533119; doi:10.1038/ncomms8919)
Supplement: Supplementary Information — Supplementary Figures 1-46, Supplementary Table 1, Supplementary Note 1, Supplementary Methods and Supplementary References [file ncomms8919-s1.pdf]

Supplementary Figures

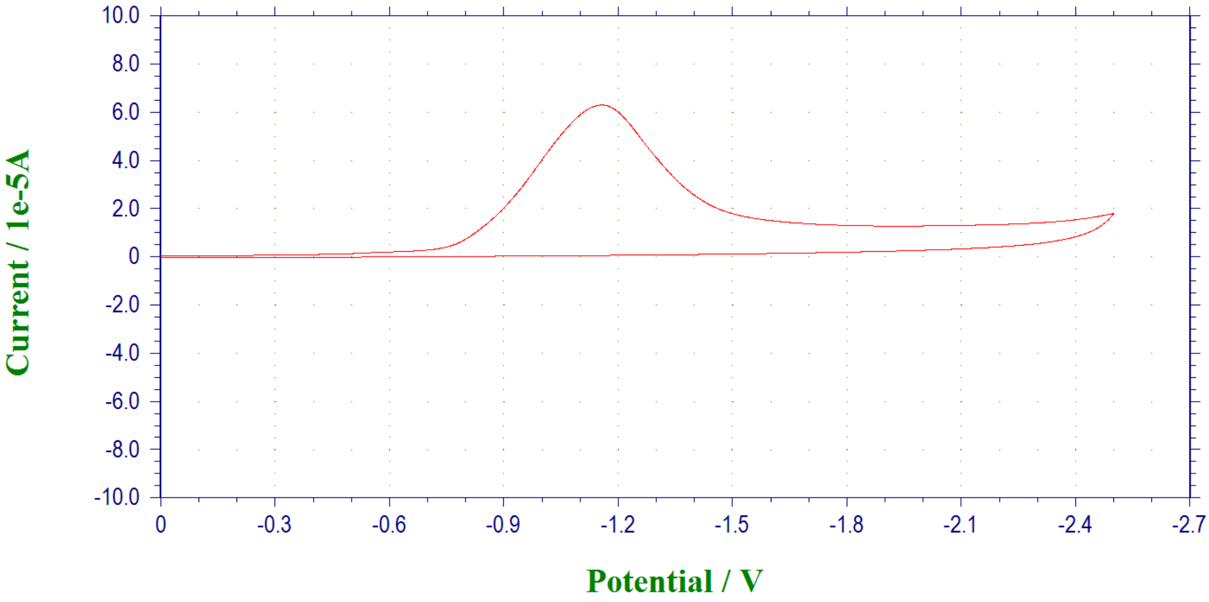

Supplementary Figure 1 | Voltammetry Measurements. Cyclic voltammetry measurements of the pyridine-*N*-oxide/TFAA adduct were performed with a model 1000 series multi-potentiostat from CH Instruments. Measurements were performed with a glassy carbon working electrode, Pt auxiliary electrode, Ag/AgCl reference electrode, Bu_4NPF_6 electrolyte (0.1M in MeCN), and analyte (pyridine-*N*-oxide:TFAA, 1:1, 0.01M) with a sweep rate of 10 mV s^{-1}. An irreversible reduction is observed ($E_{1/2}^{red}$ = –1.21 vs SCE). Onset reduction is observable near –0.8 V vs SCE.

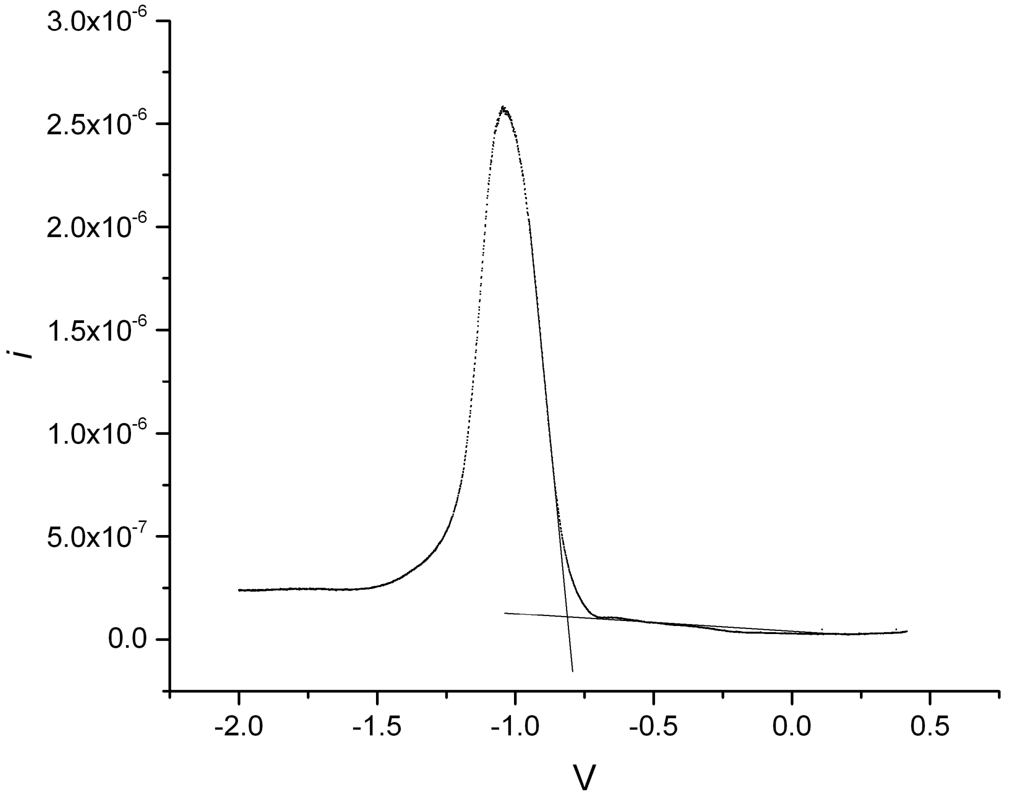

Supplementary Figure 2 | Differential Pulse Voltammetry of the TFAA/pyridine-*N*-oxide adduct. To obtain a more reliable reduction potential (and reduction onset) independent of sweep rate, differential pulsed voltammetry was performed on an identically prepared sample and cell setup with the following settings: Incr E (V) = 0.001, Amplitude (V) = 0.005, Pulse Width (sec) = 0.05, Sampling Width (sec) = 0.01, Pulse Period (sec) = 0.5. The results obtained indicate a slightly more positive reduction potential than that obtained by CV. $E_{1/2}^{red}$ = –1.10 V vs. SCE, onset reduction = –0.86 V vs SCE.

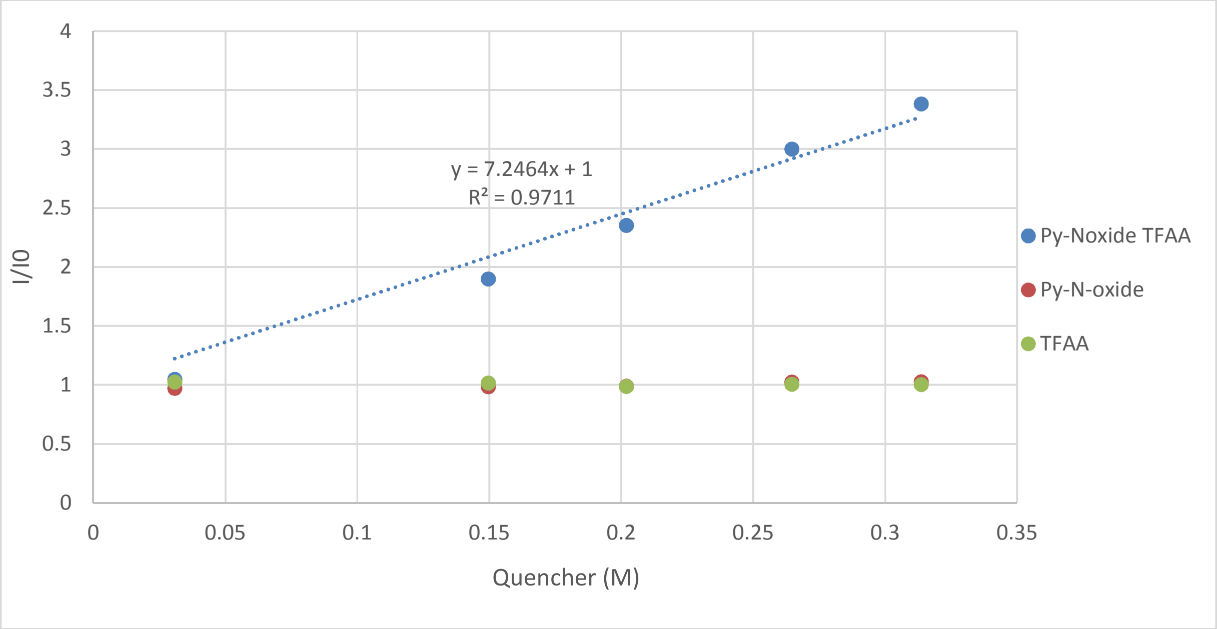

Supplementary Figure 3 | Emission Quenching Experiments. Quenching data was obtained using a Fluoromax-2 Fluorimeter. All quenching data was recorded using a quartz cuvette with a stir bar at between 24.5 and 25.5°C with Ru(bpy)$_3$Cl$_2$ (9.97 x10^{-6} M) in non-degassed MeCN. Excitation was performed at 452 nm with emission measured at 615 nm. All values are the average of 3 measurements. The combined pyridine-*N*-oxide TFAA was run at 1:1.1 equivalents (pyridine-*N*-oxide:TFAA).

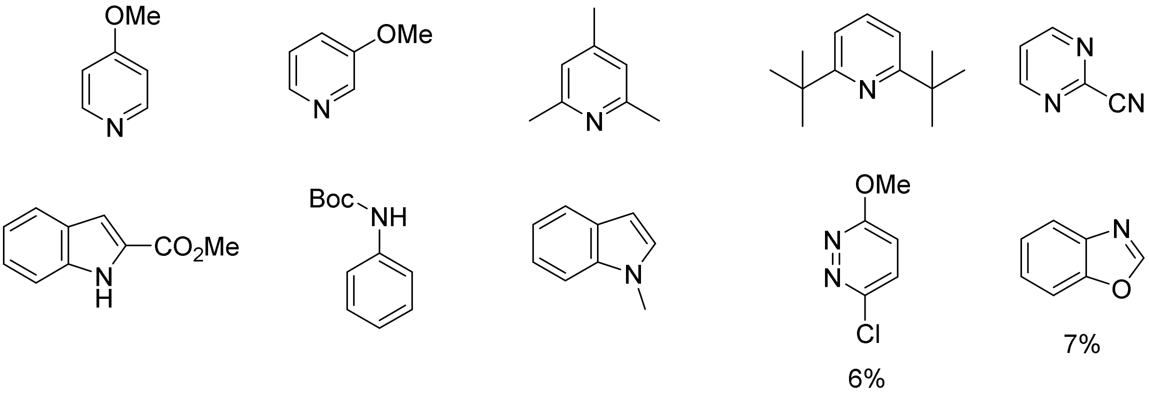

Supplementary Figure 4 | Failed Substrates. The substrates listed above failed to provide trifluoromethylated products in sufficient quantities under the described conditions. Free NH groups were acylated. N-methylindole underwent acylation at the 3-position.

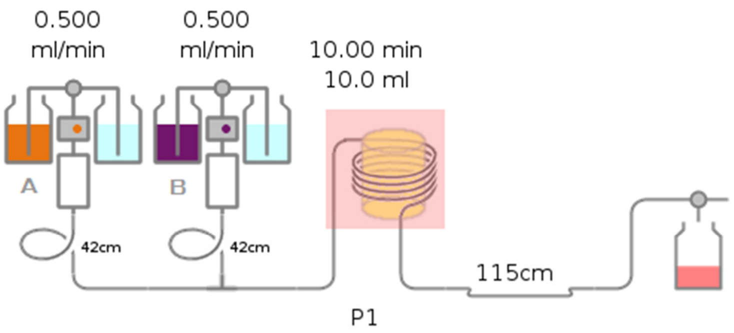

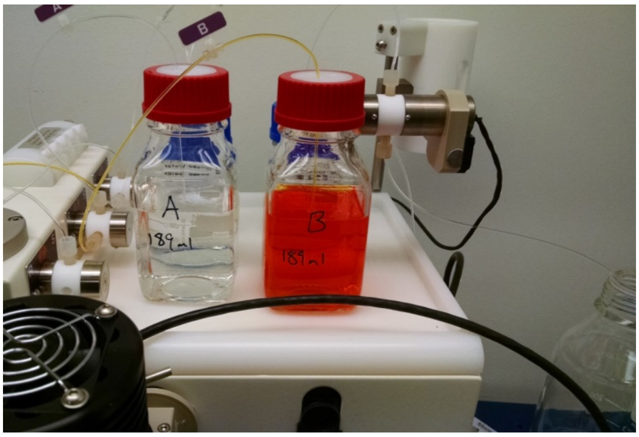

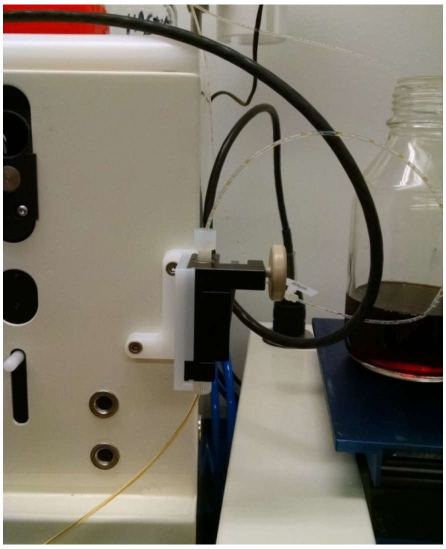

Supplementary Figure 5 | Scale-up in flow. Schematic of Vapourtec® E-series photo reactor setup used for reaction scale-up (top), with images of the prepared solutions (left) and observable outgassing of the reaction solution as it exits the back-pressure regulator (right). Solutions A and B are referred to in the flow procedure, which can be found in the Supplementary Methods section.

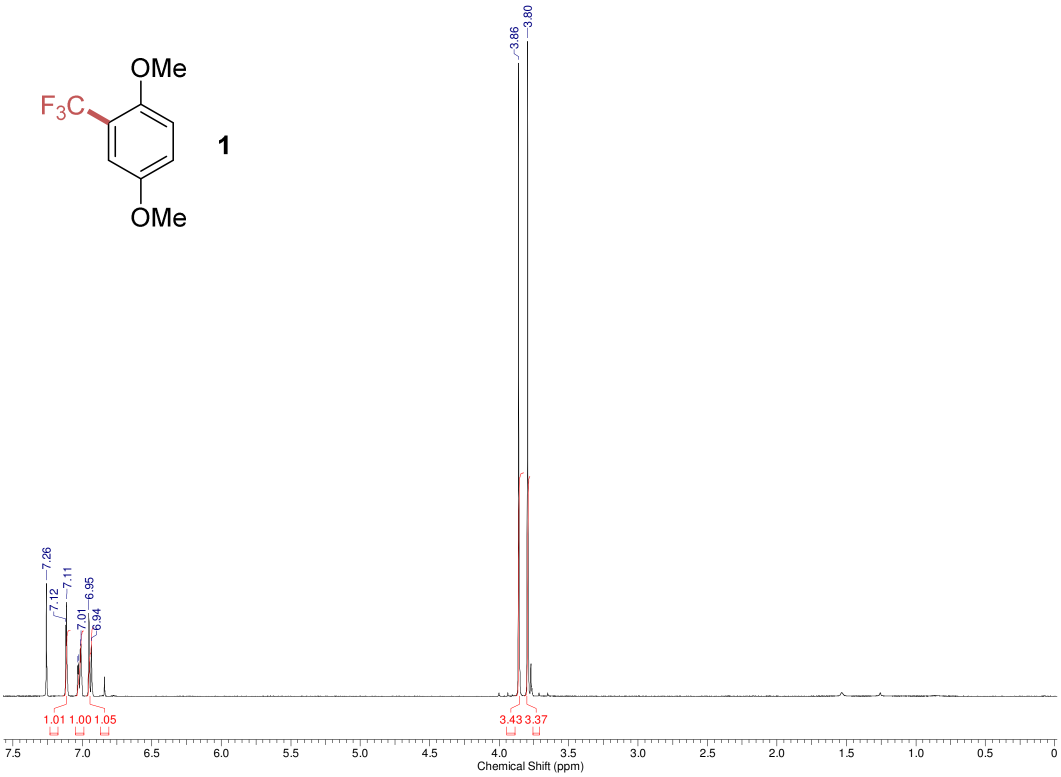

Supplementary Figure 6 | ^{1}H NMR spectrum of 1,4-dimethoxy-2-(trifluoromethyl)benzene. (500 MHz, $CDCl_3$, 298 K).

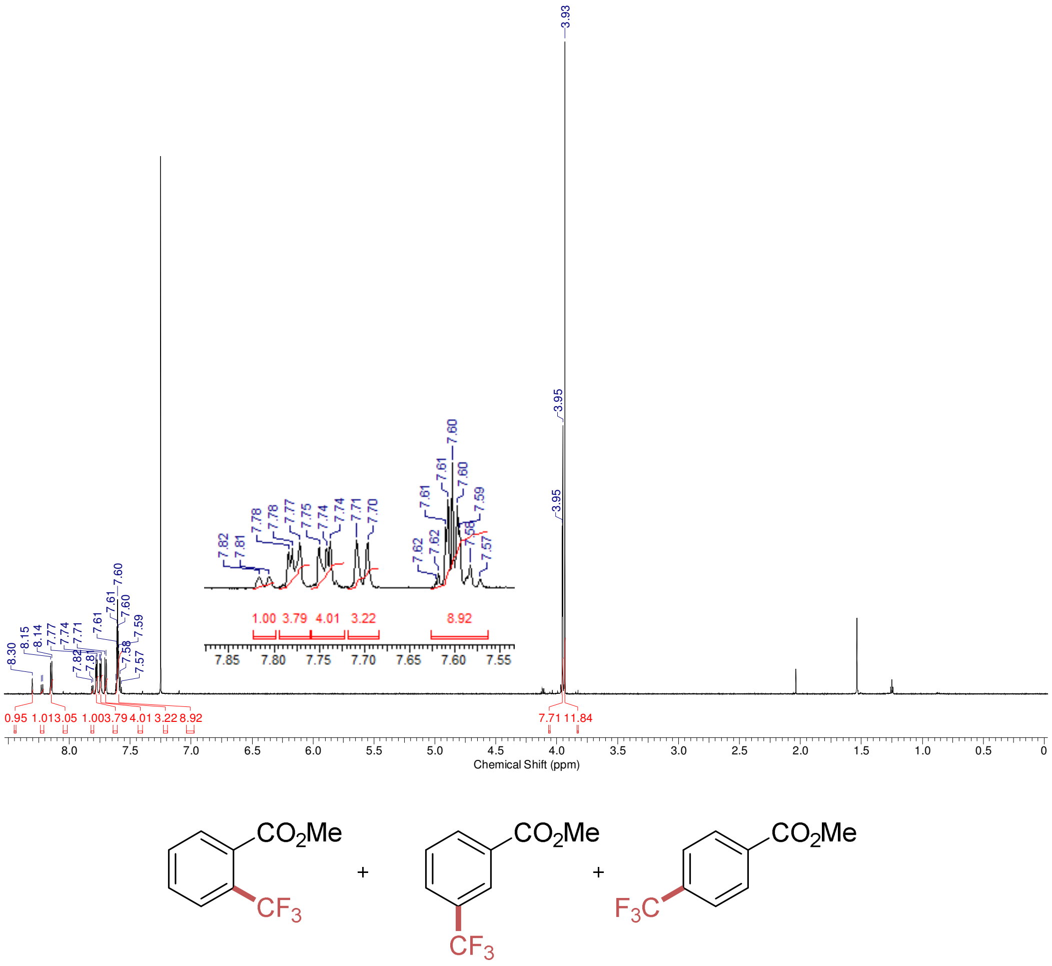

2

Supplementary Figure 7 | ^{1}H NMR spectrum of a mixture of methyl 2-(trifluoromethyl)benzoate, methyl 3-(trifluoromethyl)benzoate, and methyl 4- (trifluoromethyl)benzoate. (500 MHz, $CDCl_3$, 298 K).

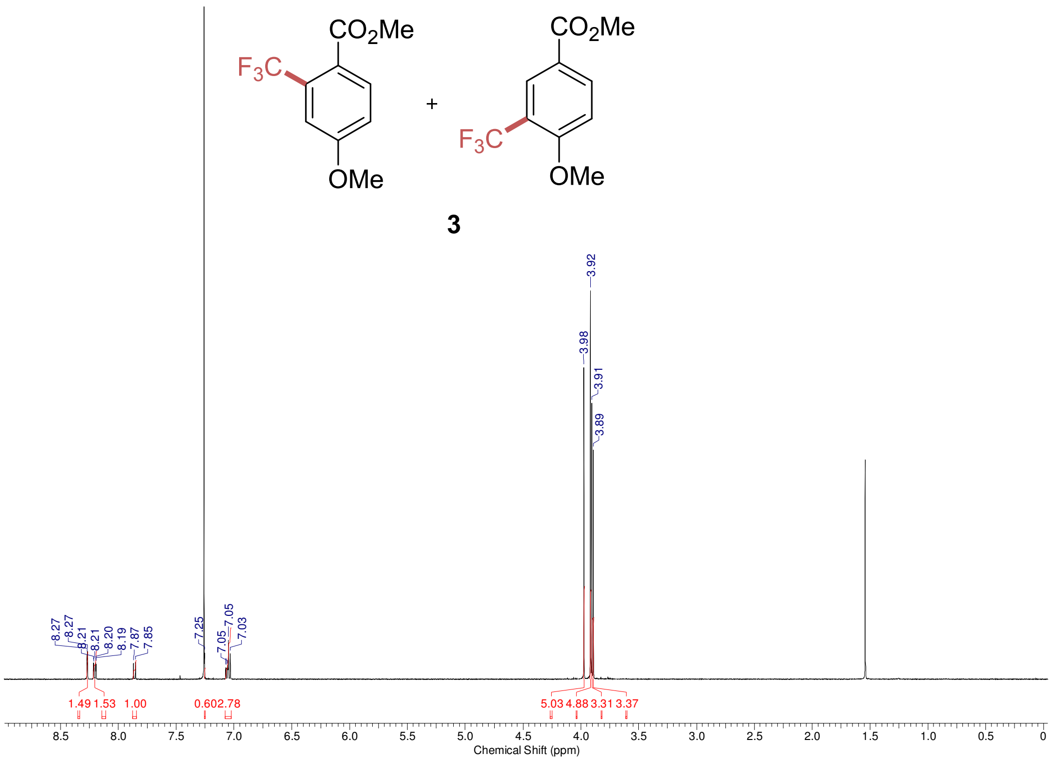

Supplementary Figure 8 | ^{1}H NMR Spectrum of a mixture of methyl 4-methoxy-2-(trifluoromethyl)benzene and methyl 4-methoxy-3-(trifluoromethyl)benzene. (500 MHz, $CDCl_3$, 298 K).

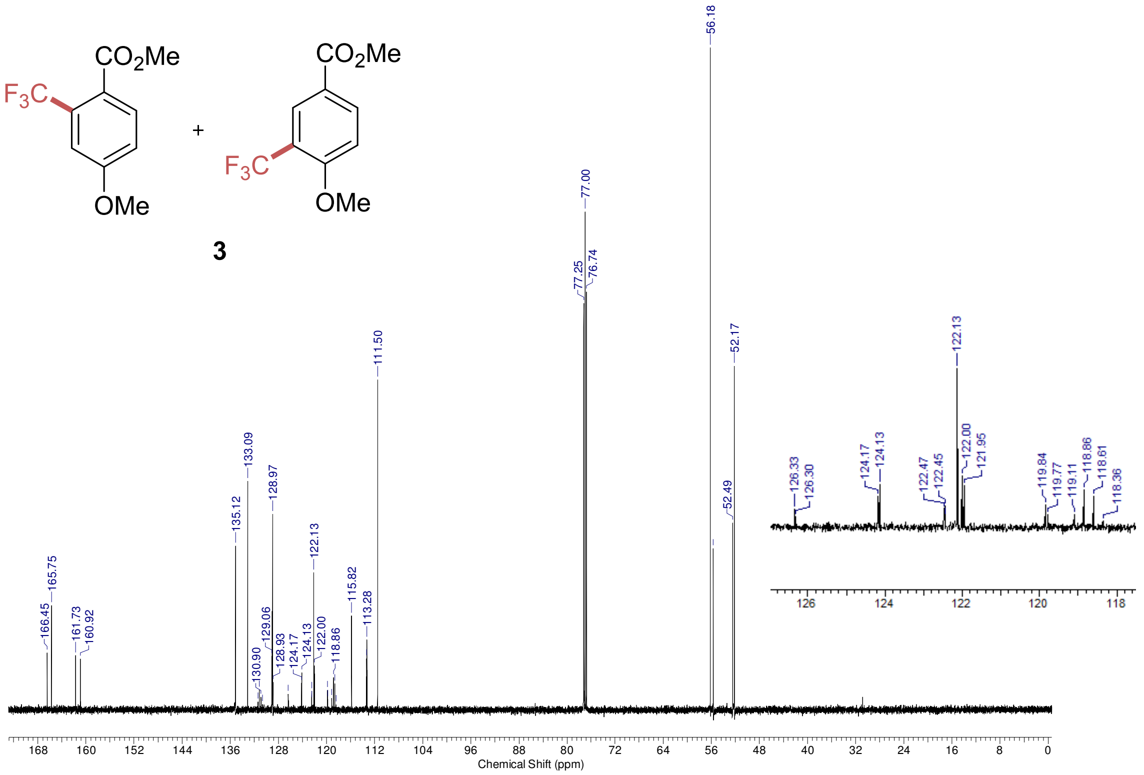

Supplementary Figure 9 | ^{13}C NMR Spectrum of a mixture of methyl 4-methoxy-2-(trifluoromethyl)benzene and methyl 4-methoxy-3-(trifluoromethyl)benzene. (125 MHz, $CDCl_3$, 298 K).

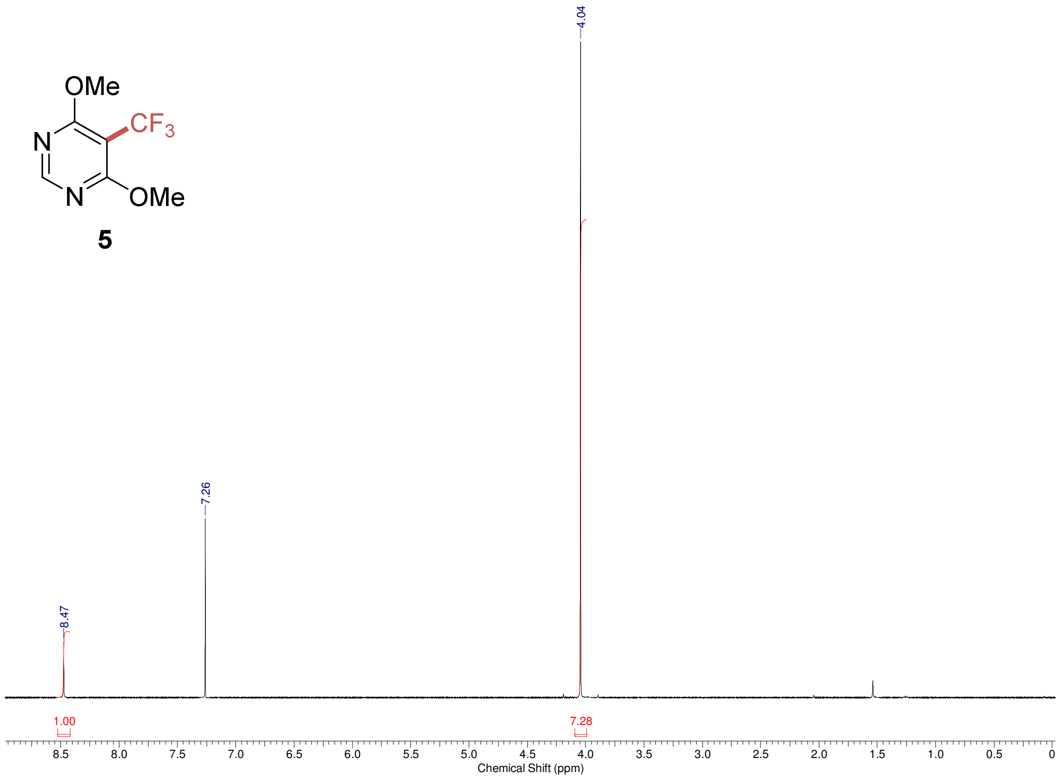

Supplementary Figure 10 | ^{1}H NMR Spectrum of 4,6-dimethoxy-5-(trifluoromethyl)pyrimidine. (500 MHz, $CDCl_3$, 298 K).

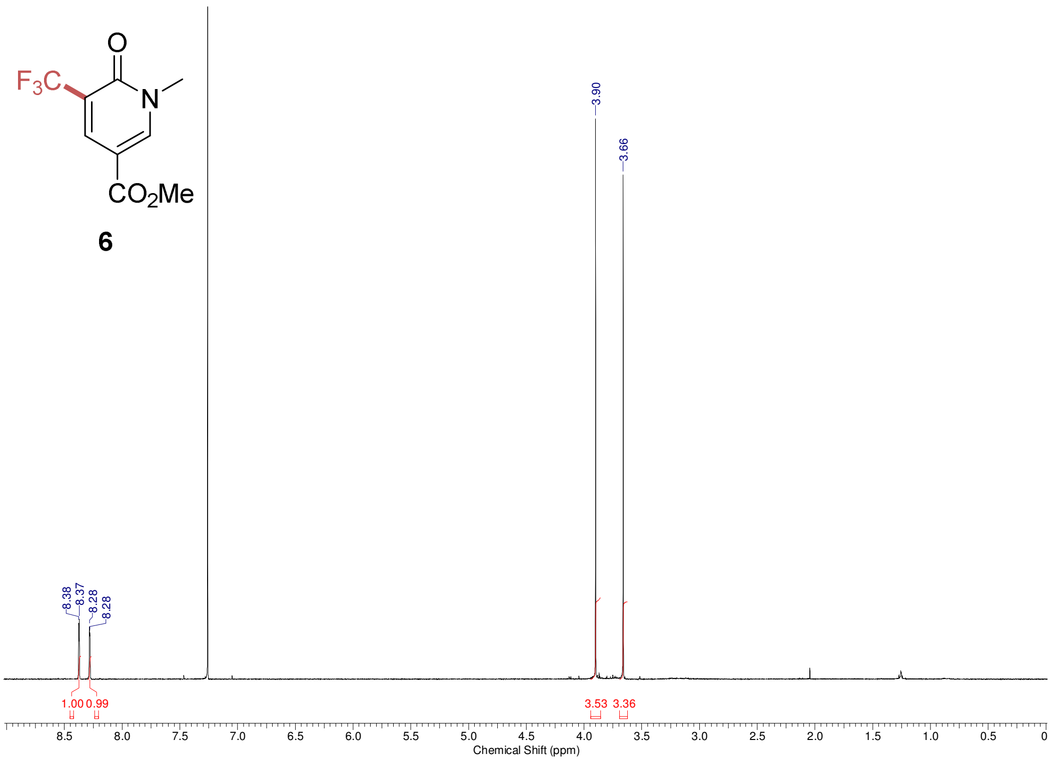

Supplementary Figure 11 | ^{1}H NMR Spectrum of methyl 1-methyl-6-oxo-5-(trifluoromethyl)-1,6-dihydropyridine-3-carboxylate. (500 MHz, $CDCl_3$, 298 K).

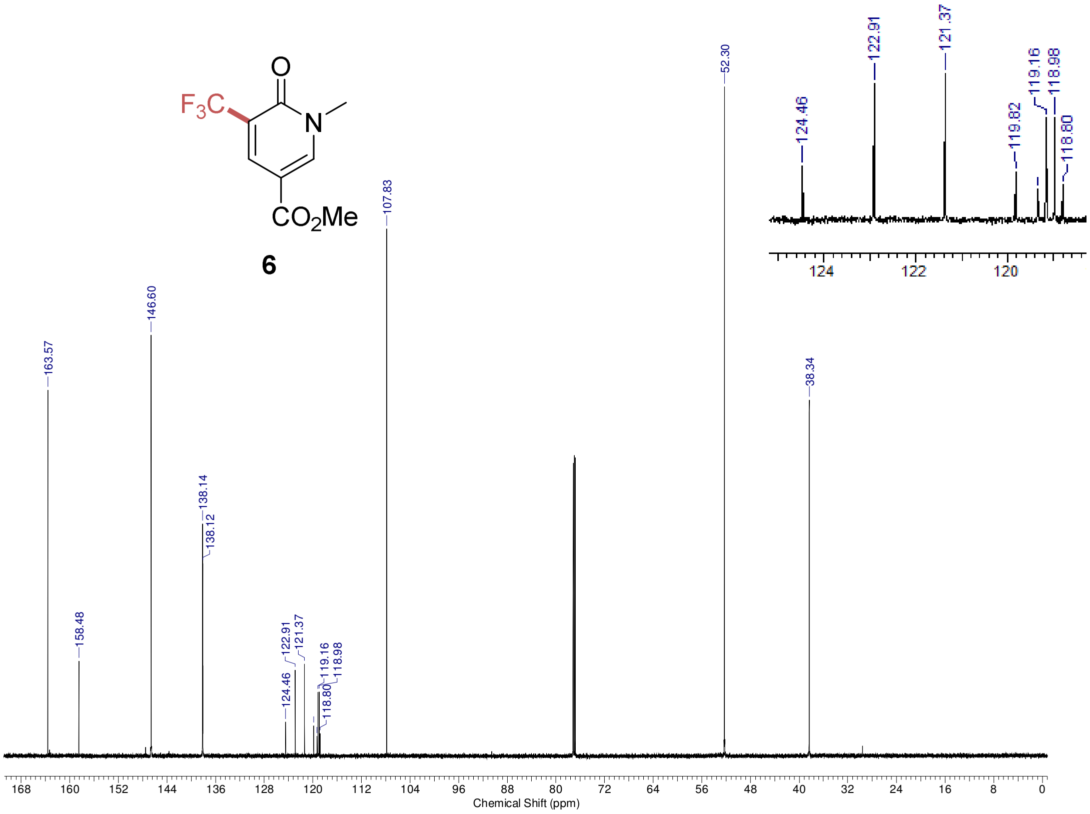

Supplementary Figure 12 | ^{13}C NMR Spectrum of methyl 1-methyl-6-oxo-5-(trifluoromethyl)-1,6-dihydropyridine-3-carboxylate. (175 MHz, $CDCl_3$, 298K).

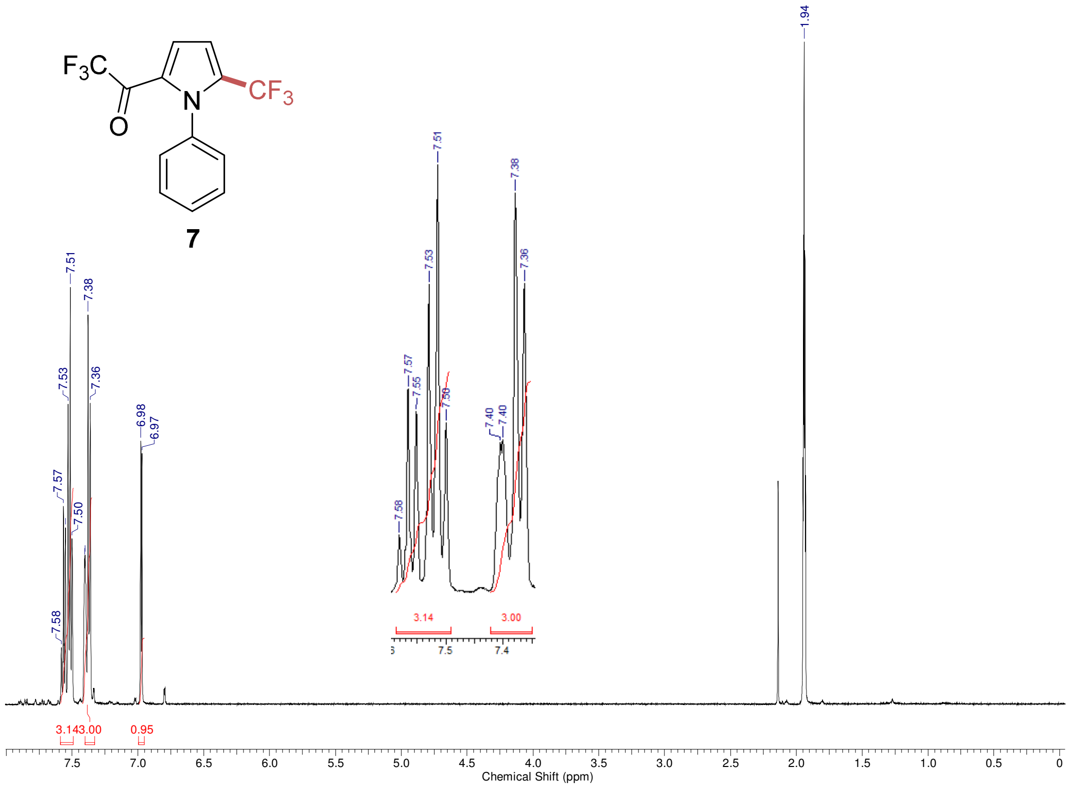

Supplementary Figure 13 | ^{1}H NMR spectrum of 2,2,2-trifluoro-1-(1-phenyl-5-(trifluoromethyl)-1H-pyrrol-2-yl)ethan-1-one. (500 MHz, $CDCl_3$, 298 K).

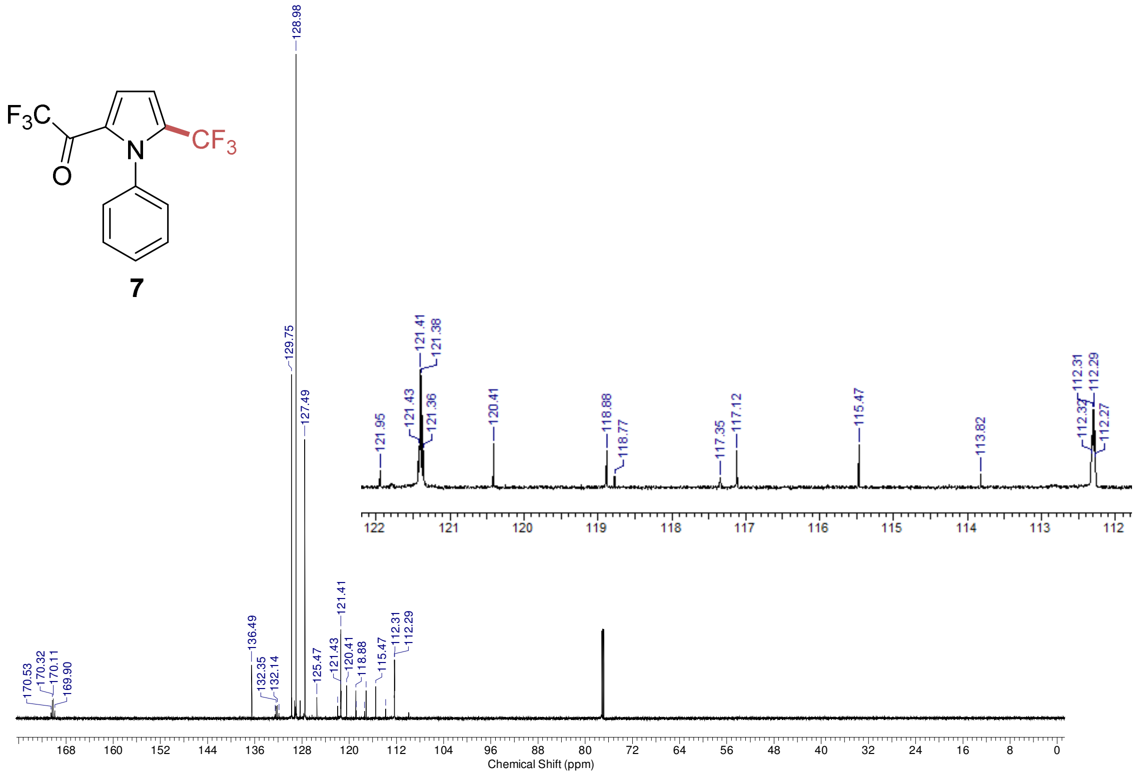

Supplementary Figure 14 | ^{13}C NMR spectrum of 2,2,2-trifluoro-1-(1-phenyl-5-(trifluoromethyl)-1H-pyrrol-2-yl)ethan-1-one. (175 MHz, $CDCl_3$, 298 K).

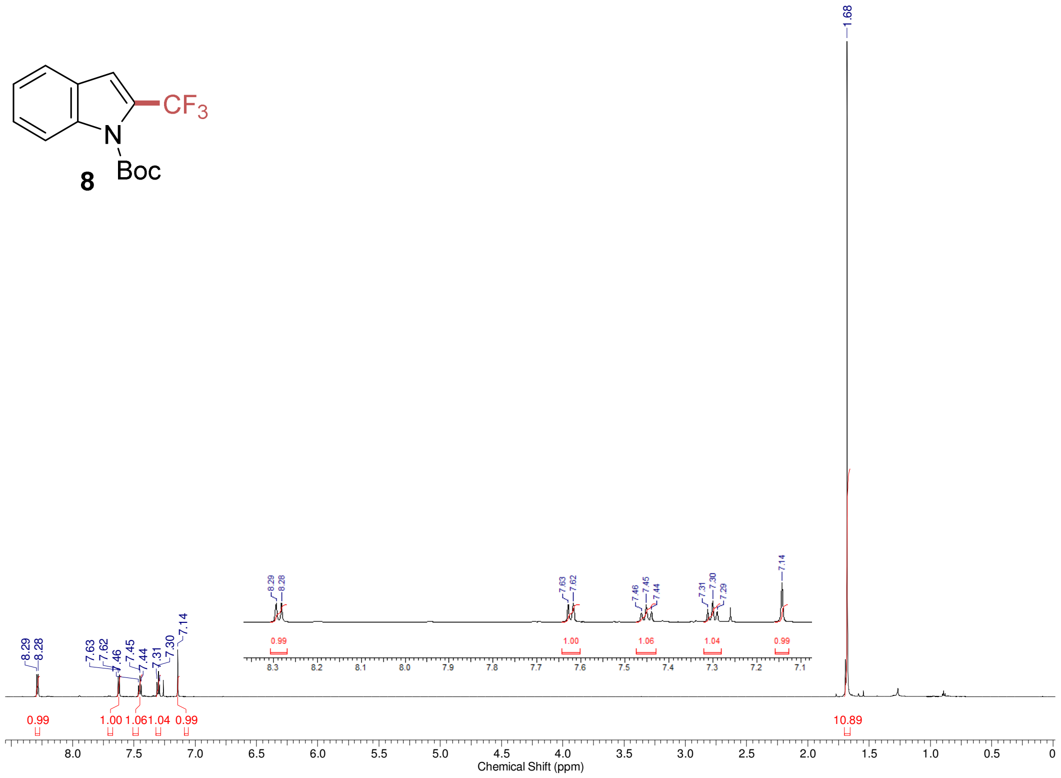

Supplementary Figure 15 | ^{1}H NMR spectrum of *tert*-butyl 2-(trifluoromethyl)-1*H*-indole-1-carboxylate. (500 MHz, $CDCl_3$, 298 K).

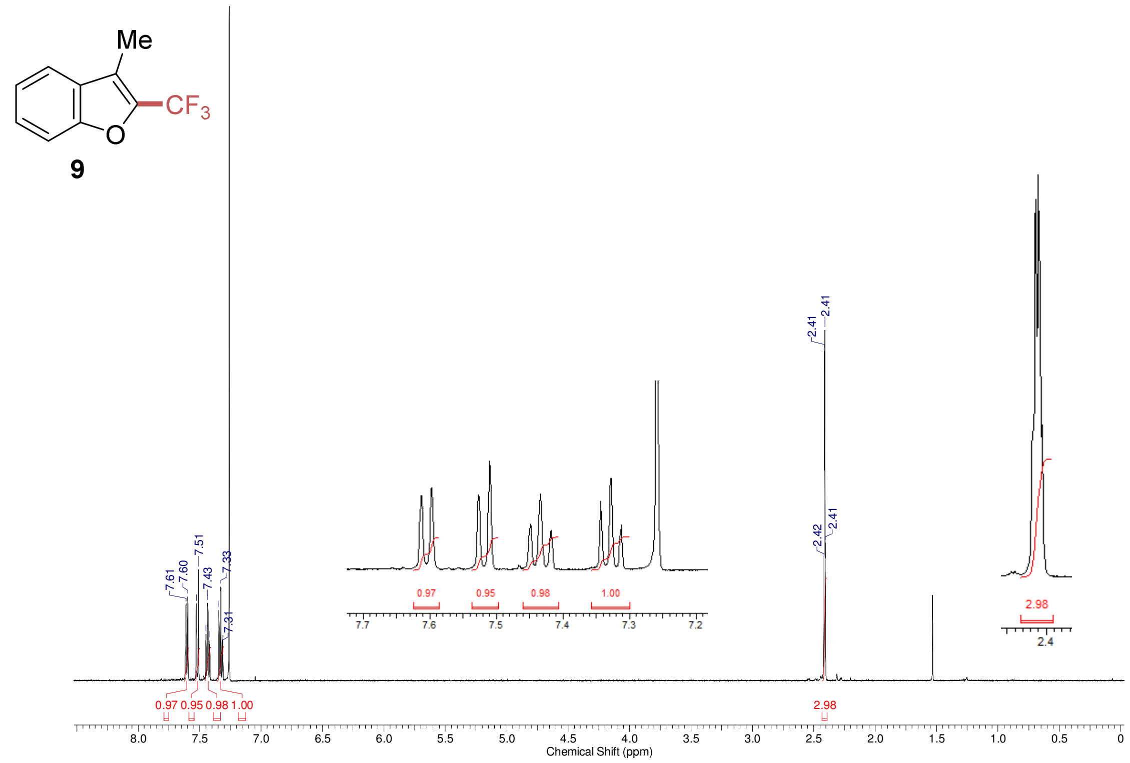

Supplementary Figure 16 | ^{1}H NMR spectrum of 3-methyl-2-(trifluoromethyl)benzofuran. (500 MHz, $CDCl_3$, 298 K).

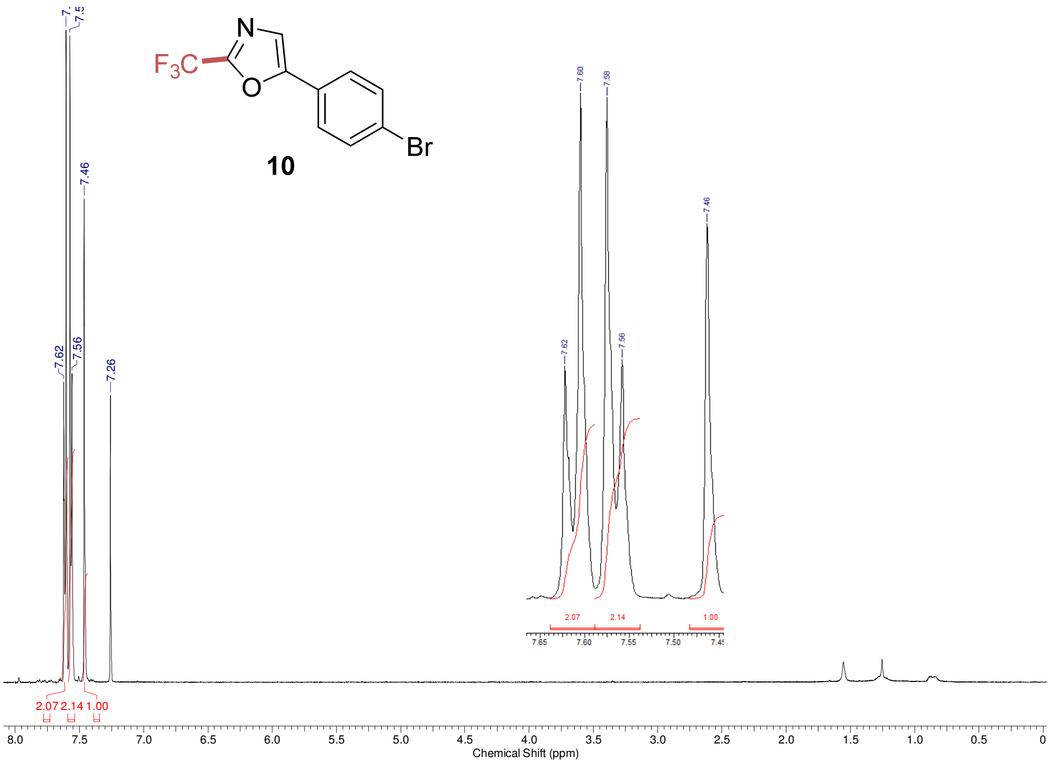

Supplementary Figure 17 | ^{1}H NMR spectrum of 5-(4-bromophenyl)-2-(trifluoromethyl)oxazole. (700 MHz, $CDCl_3$, 298 K).

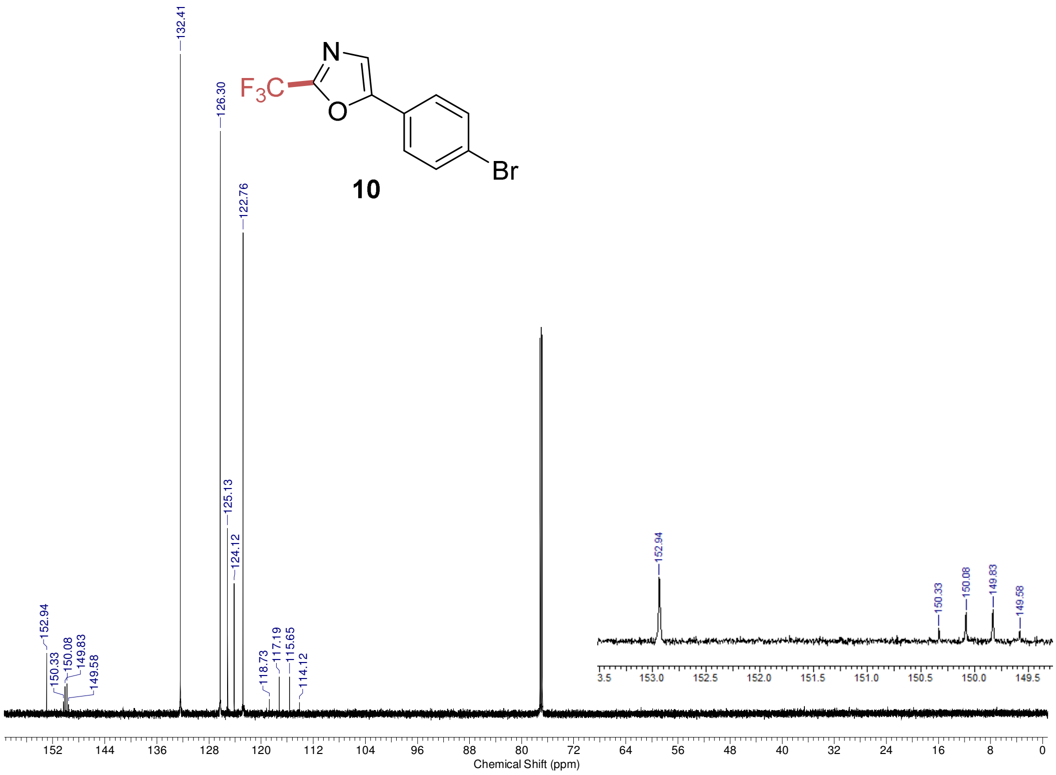

Supplementary Figure 18 | ^{13}C NMR spectrum of 5-(4-bromophenyl)-2-(trifluoromethyl)oxazole. (175 MHz, $CDCl_3$, 298 K).

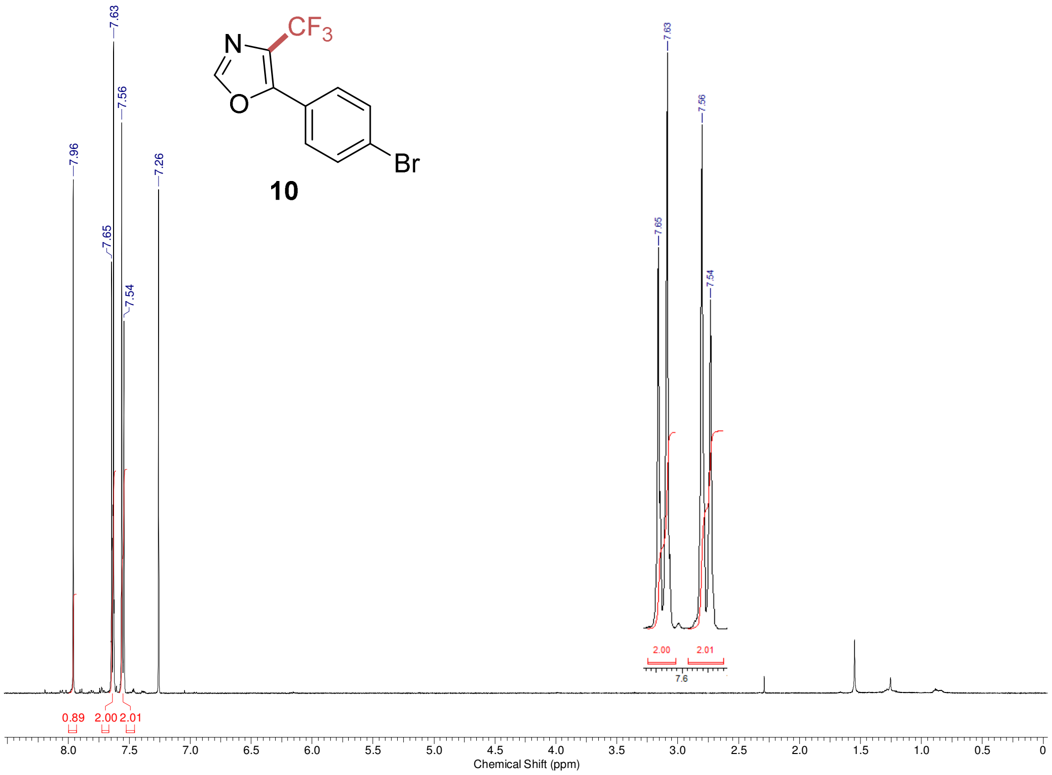

Supplementary Figure 19 | ^{1}H NMR spectrum of 5-(4-bromophenyl)-4-(trifluoromethyl)oxazole. (700 MHz, $CDCl_3$, 298 K).

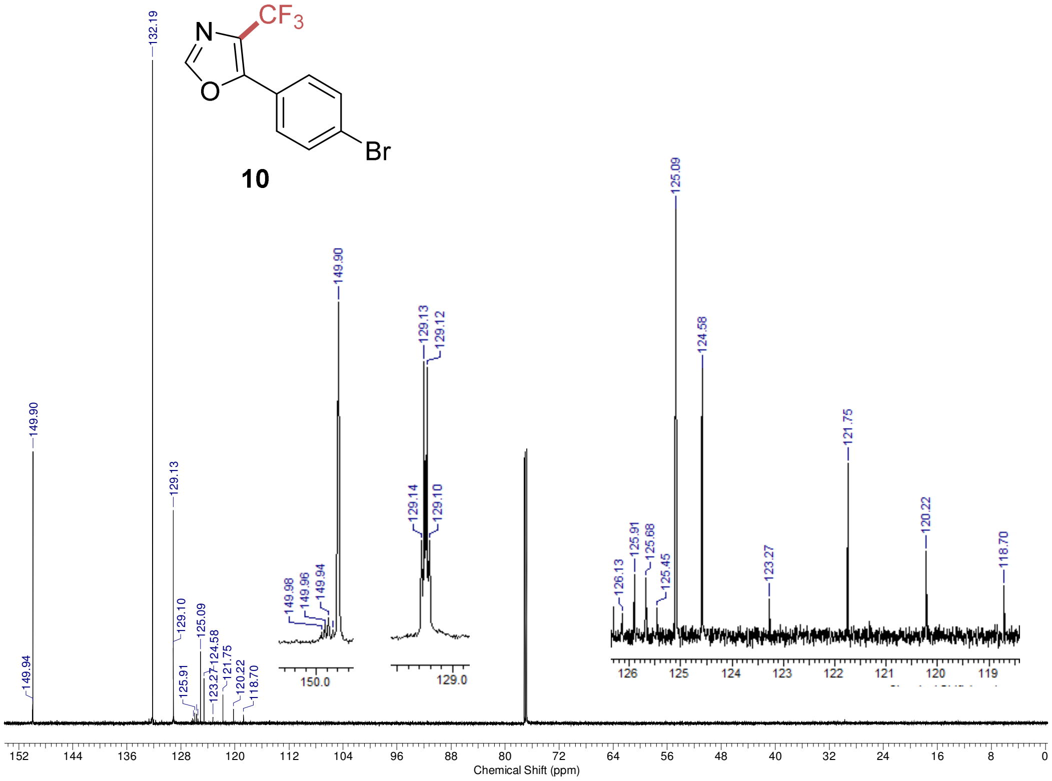

Supplementary Figure 20 | ^{13}C NMR spectrum of 5-(4-bromophenyl)-4-(trifluoromethyl)oxazole. (175 MHz, $CDCl_3$, 298 K).

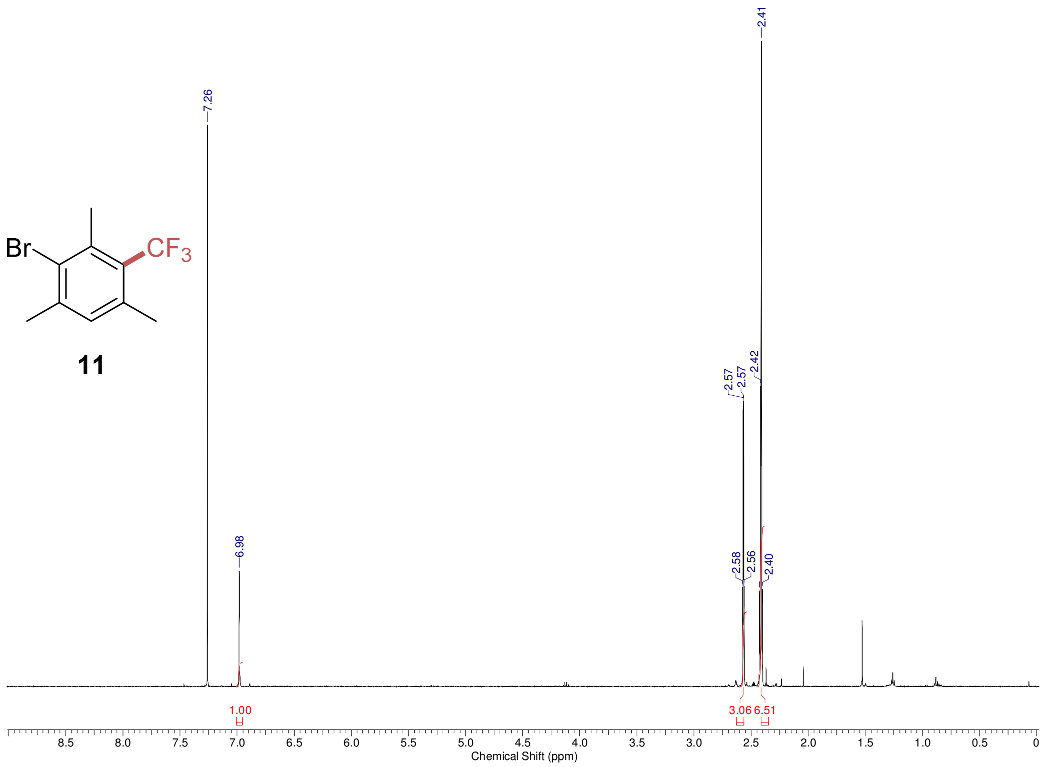

Supplementary Figure 21 | ^{1}H NMR spectrum of 2-bromo-1,3,5-trimethyl-4-(trifluoromethyl)benzene. (500 MHz, $CDCl_3$, 298 K).

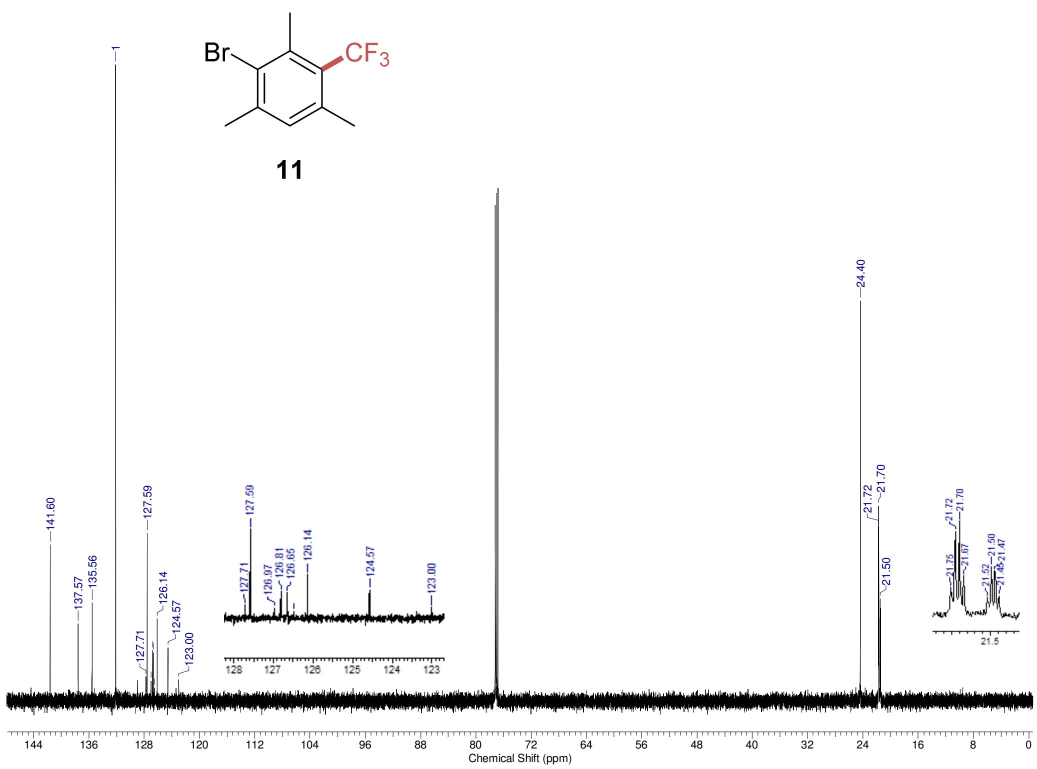

Supplementary Figure 22 | ^{13}C NMR spectrum of 2-bromo-1,3,5-trimethyl-4-(trifluoromethyl)benzene. (175 MHz, $CDCl_3$, 298 K).

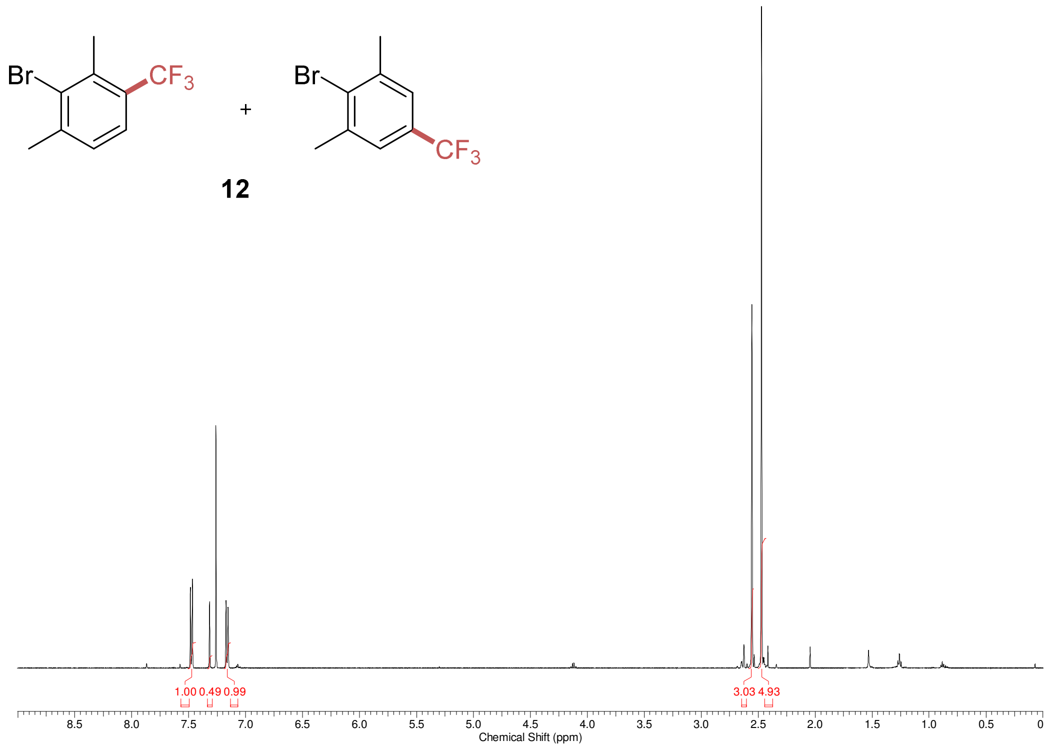

Supplementary Figure 23 | ^{1}H NMR spectrum of a mixture of 2-bromo-1,3-dimethyl-4-(trifluoromethyl)benzene and 2-bromo-1,3-dimethyl-5-(trifluoromethyl)benzene. (500 MHz, $CDCl_3$, 298 K).

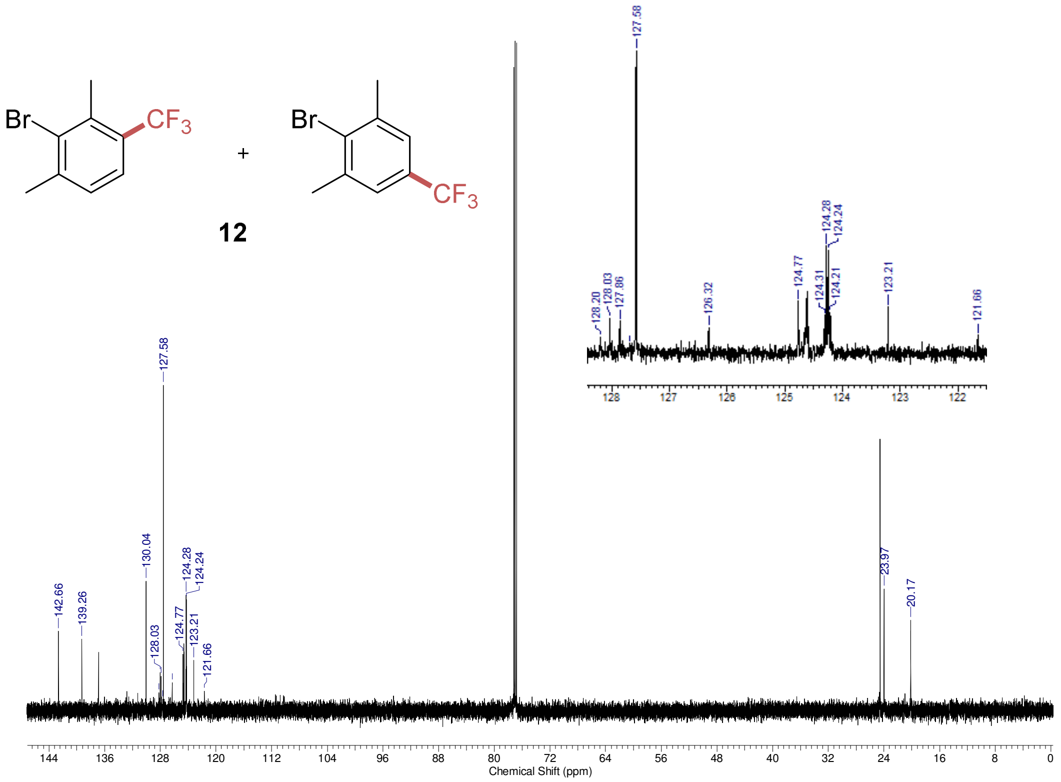

Supplementary Figure 24 | ^{13}C NMR spectrum of a mixture of 2-bromo-1,3-dimethyl-4-(trifluoromethyl)benzene and 2-bromo-1,3-dimethyl-5-(trifluoromethyl)benzene. (175 MHz, $CDCl_3$, 298 K).

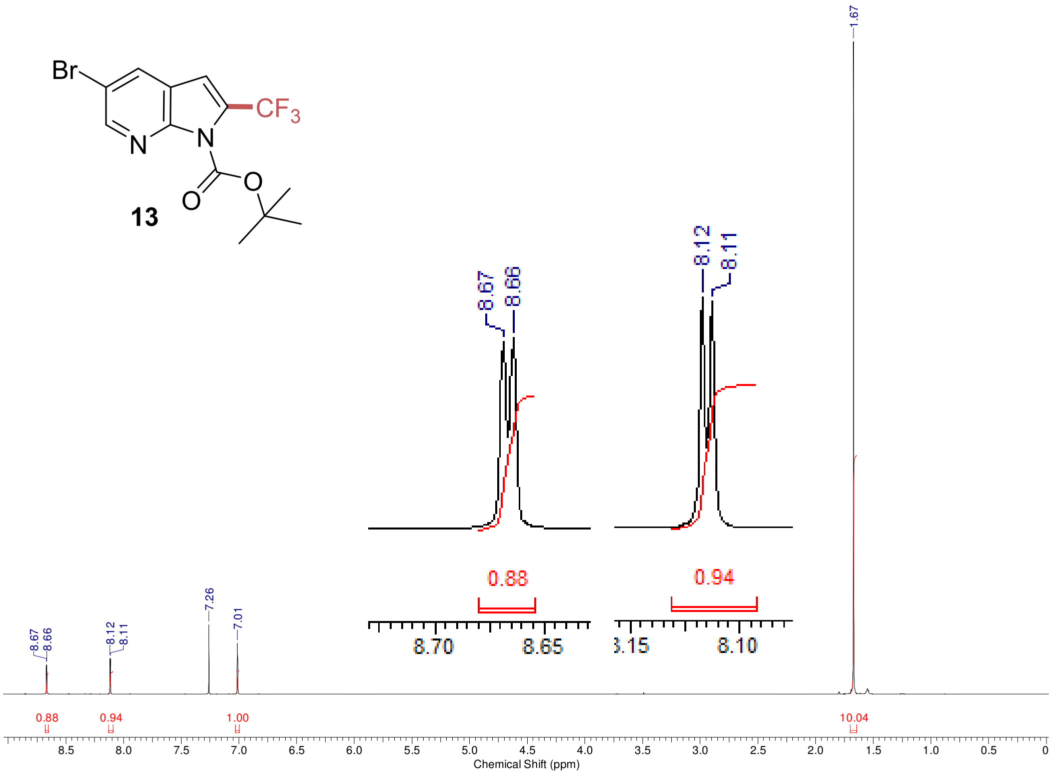

Supplementary Figure 25 | ^{1}H NMR spectrum of *tert*-butyl 5-bromo-2-(trifluoromethyl)-1H-pyrrolo[2,3-b]pyridine-1-carboxylate. (500 MHz, $CDCl_3$, 298 K).

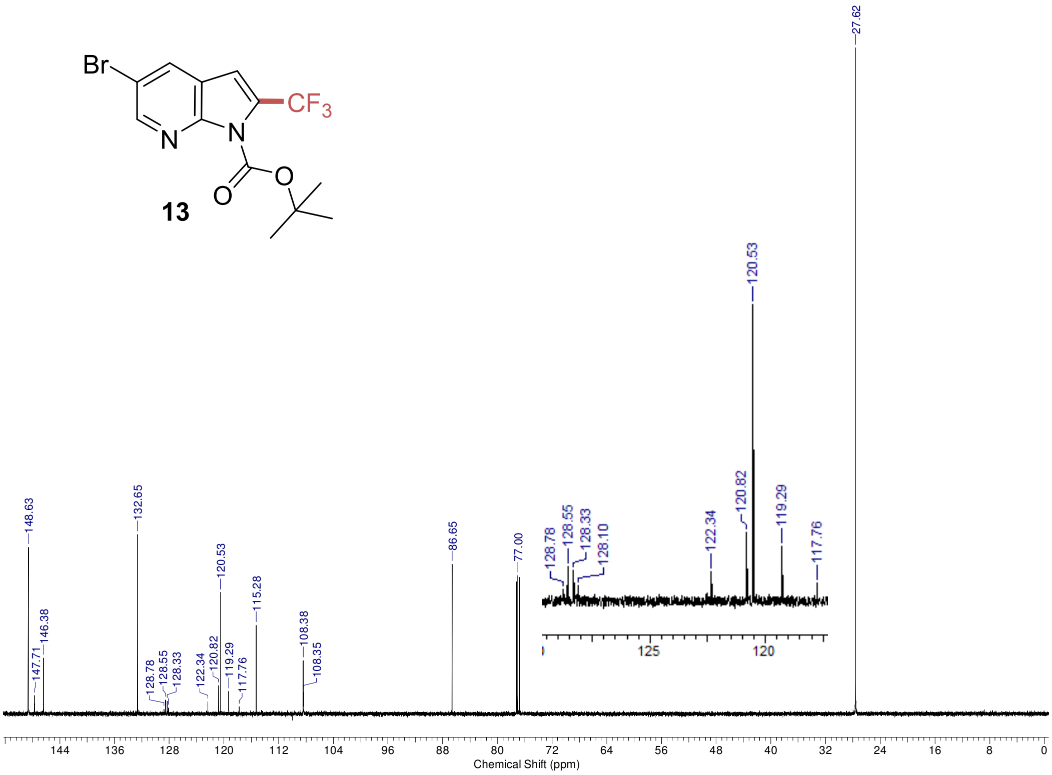

Supplementary Figure 26 | ^{13}C NMR spectrum of *tert*-butyl 5-bromo-2-(trifluoromethyl)-1H-pyrrolo[2,3-b]pyridine-1-carboxylate. (175 MHz, $CDCl_3$, 298 K).

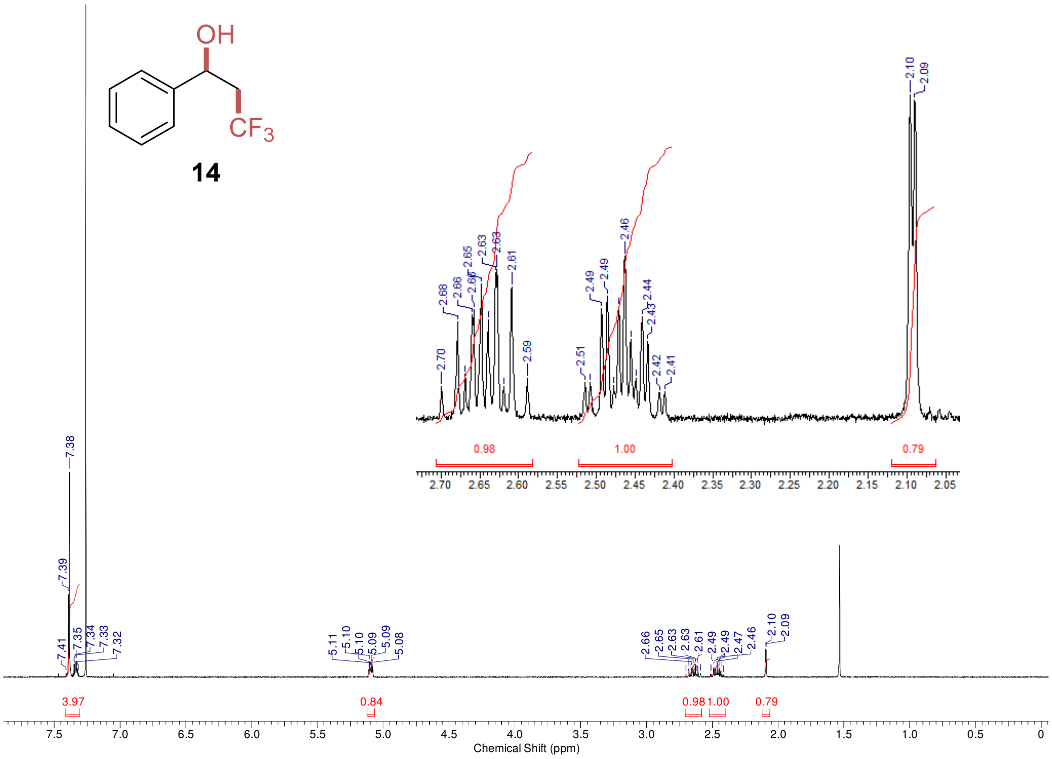

Supplementary Figure 27 | ^{1}H NMR spectrum of 3,3,3-trifluoro-1-phenylpropan-1-ol. (500 MHz, $CDCl_3$, 298 K).

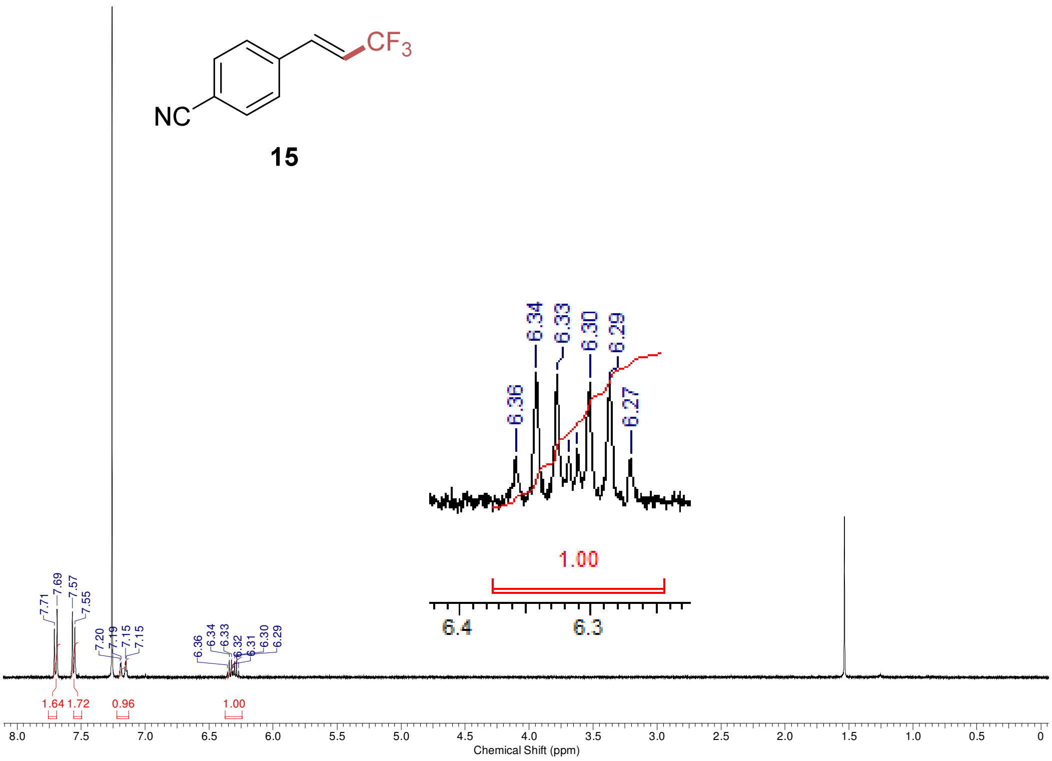

Supplementary Figure 28 | ^{1}H NMR spectrum of (*E*)-4-(3,3,3-trifluoroprop-1-en-1-yl)benzonitrile. (500 MHz, $CDCl_3$, 298 K).

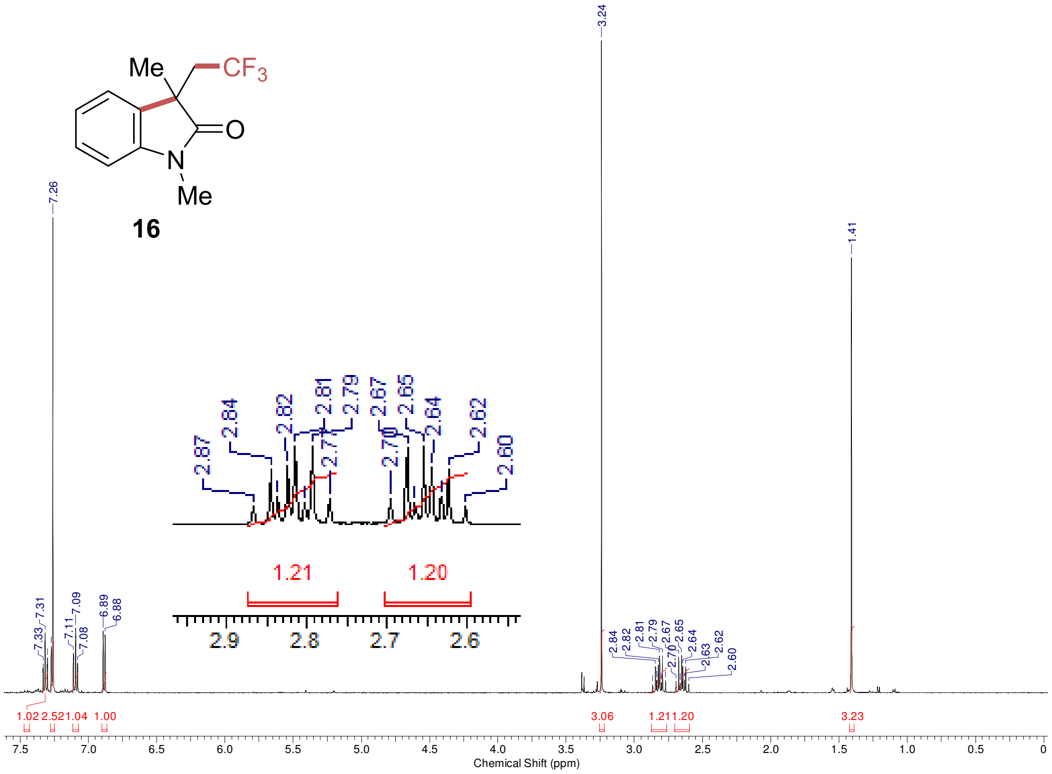

Supplementary Figure 29 | ^{1}H NMR spectrum of 1,3-dimethyl-3-(2,2,2-trifluoroethyl)indolin-2-one. (500 MHz, $CDCl_3$, 298 K).

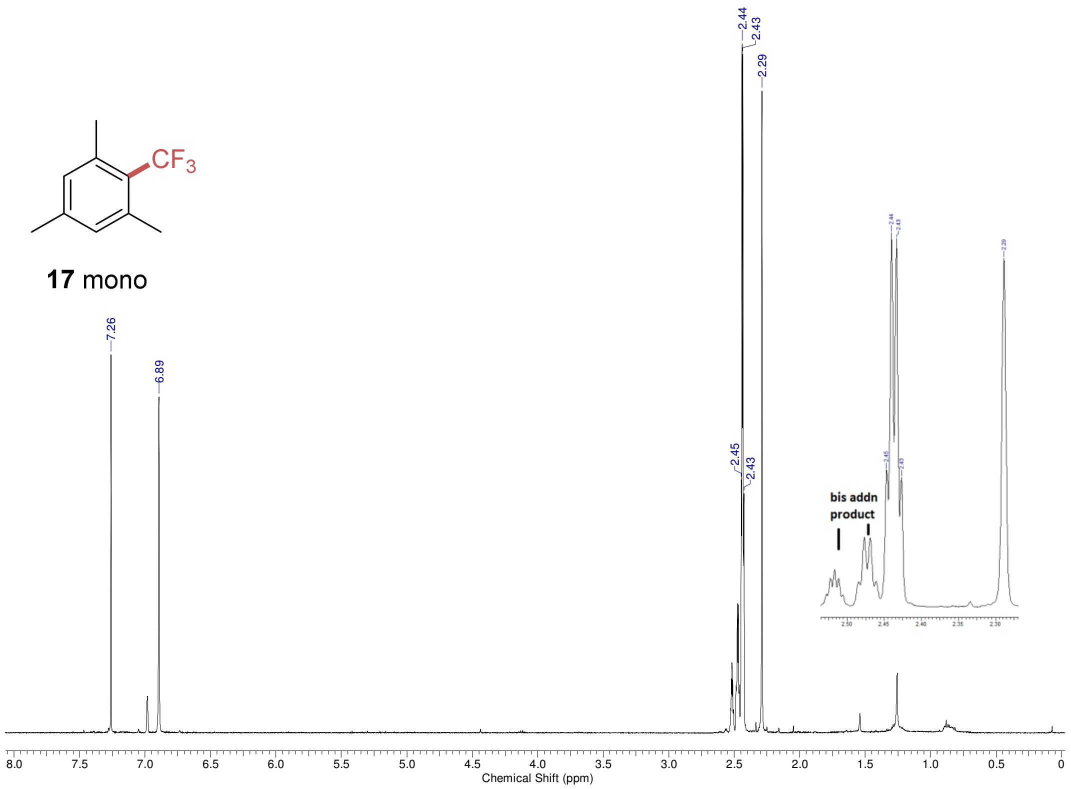

Supplementary Figure 30 | ^{1}H NMR spectrum of 1,3,5-trimethyl-2-(trifluoromethyl)benzene1. (500 MHz, $CDCl_3$, 298 K).

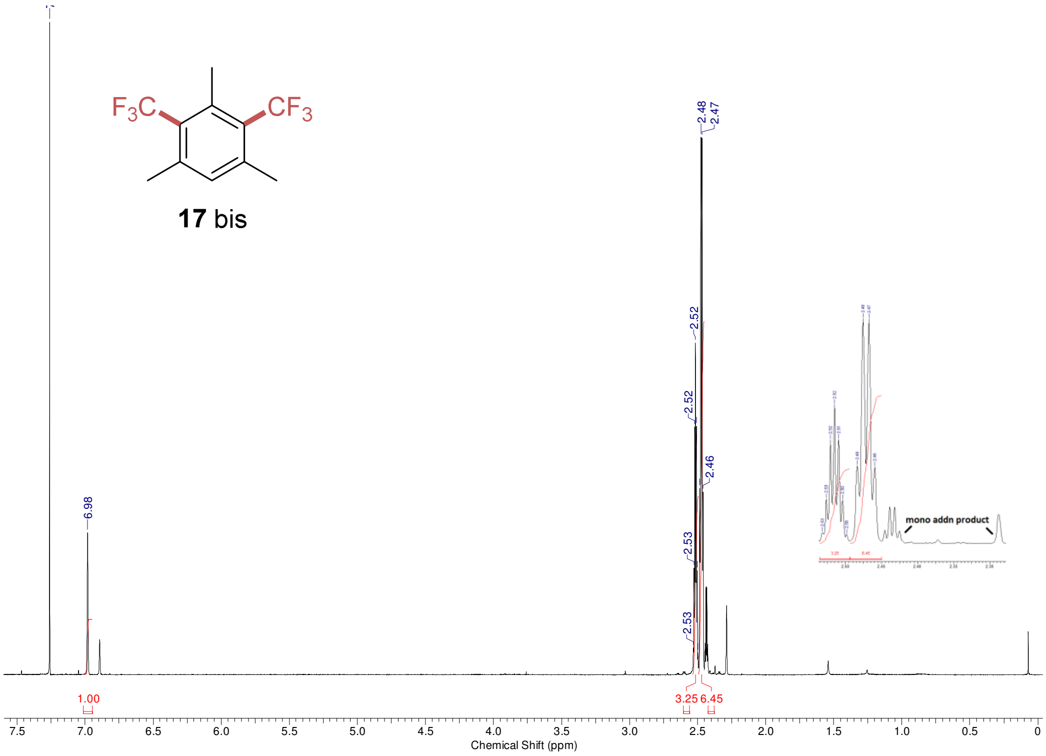

Supplementary Figure 31 | ^{1}H NMR spectrum of 1,3,5-trimethyl-2,4-bis(trifluoromethyl)benzene. (500 MHz, $CDCl_3$, 298 K).

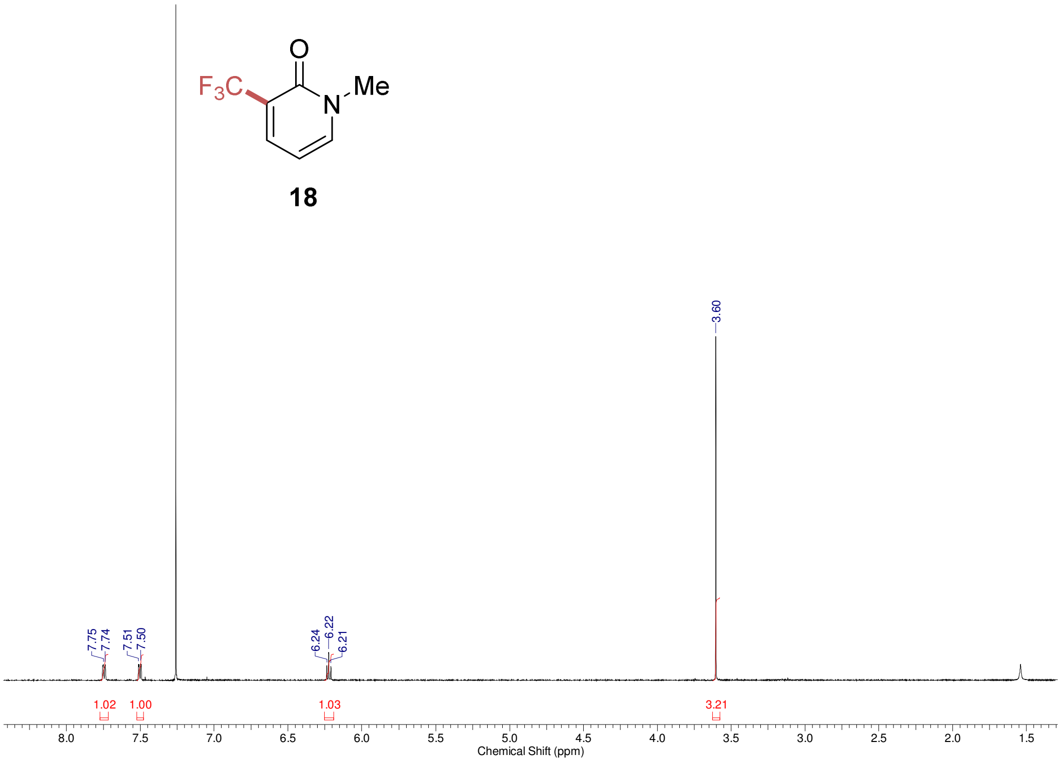

Supplementary Figure 32 | ^{1}H NMR spectrum of 1-methyl-3-(trifluoromethyl)pyridine-2(1*H*)-one. (500 MHz, $CDCl_3$, 298 K).

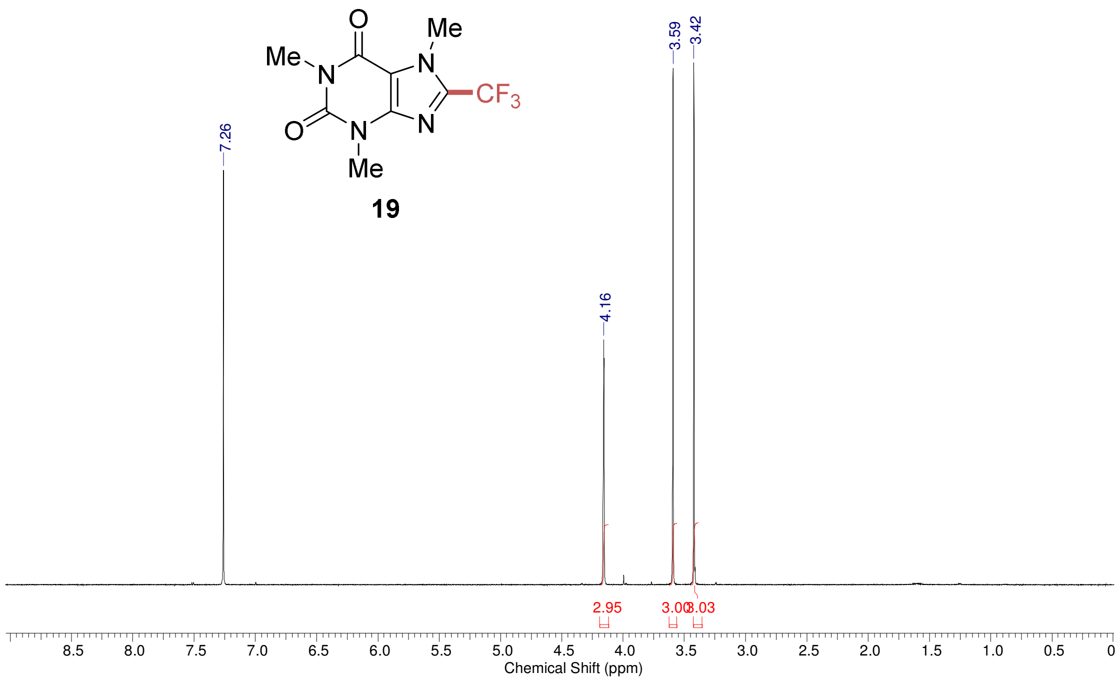

Supplementary Figure 33 | ^{1}H NMR spectrum of 1,3,7-trimethyl-8-(trifluoromethyl)-3,7-dihydro-1*H*-purine-2,6-dione. (500 MHz, $CDCl_3$, 298 K).

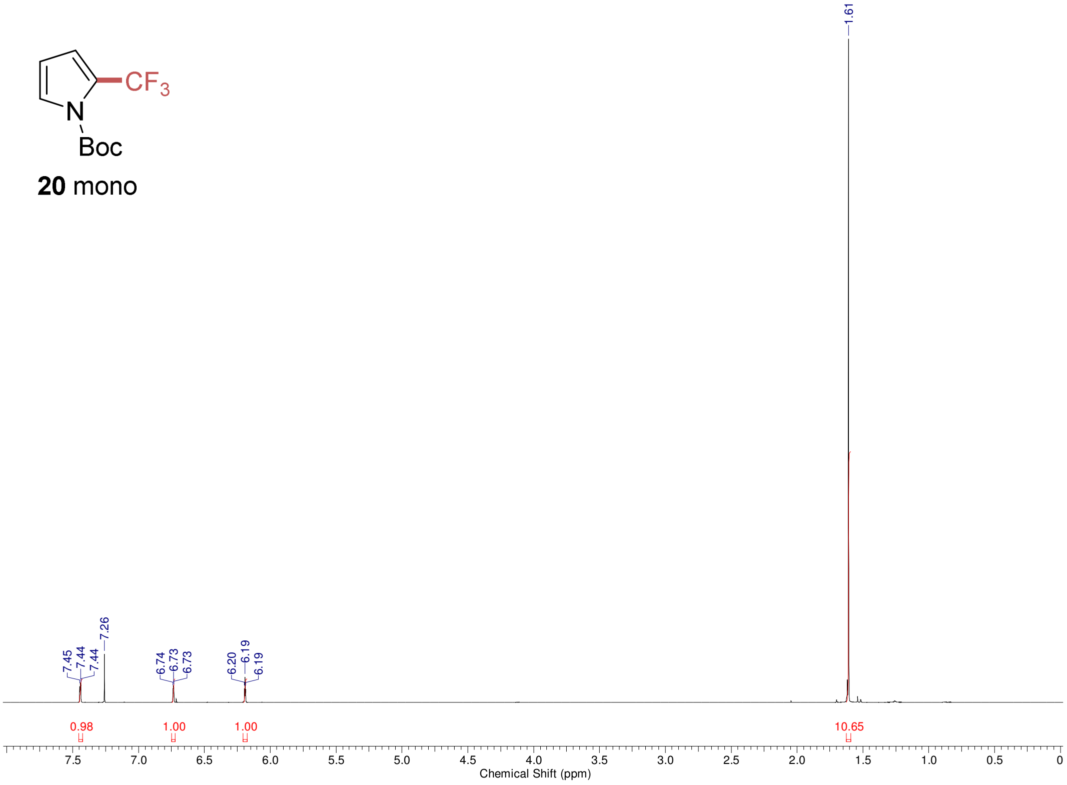

Supplementary Figure 34 | ^{1}H NMR spectrum of *tert*-butyl 2-(trifluoromethyl)-1*H*-pyrrole-1-carboxylate. (500 MHz, $CDCl_3$, 298 K).

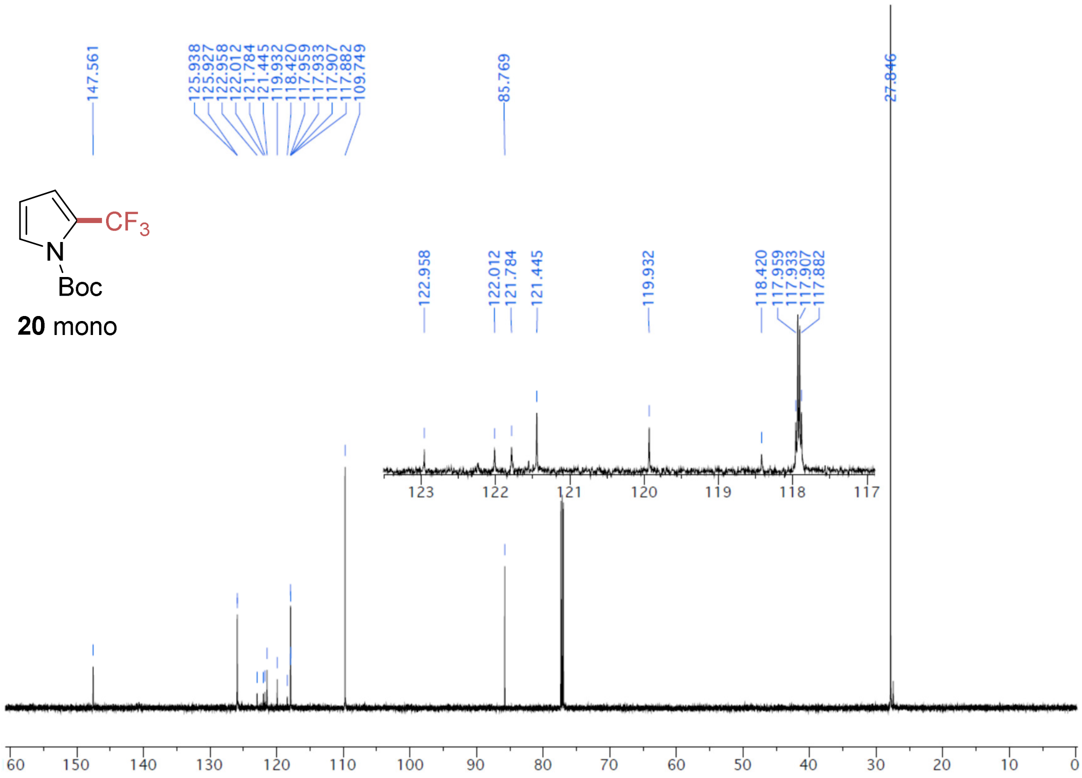

Supplementary Figure 35 | ^{13}C NMR spectrum of *tert*-butyl 2-(trifluoromethyl)-1*H*-pyrrole-1-carboxylate. (175 MHz, $CDCl_3$, 298 K).

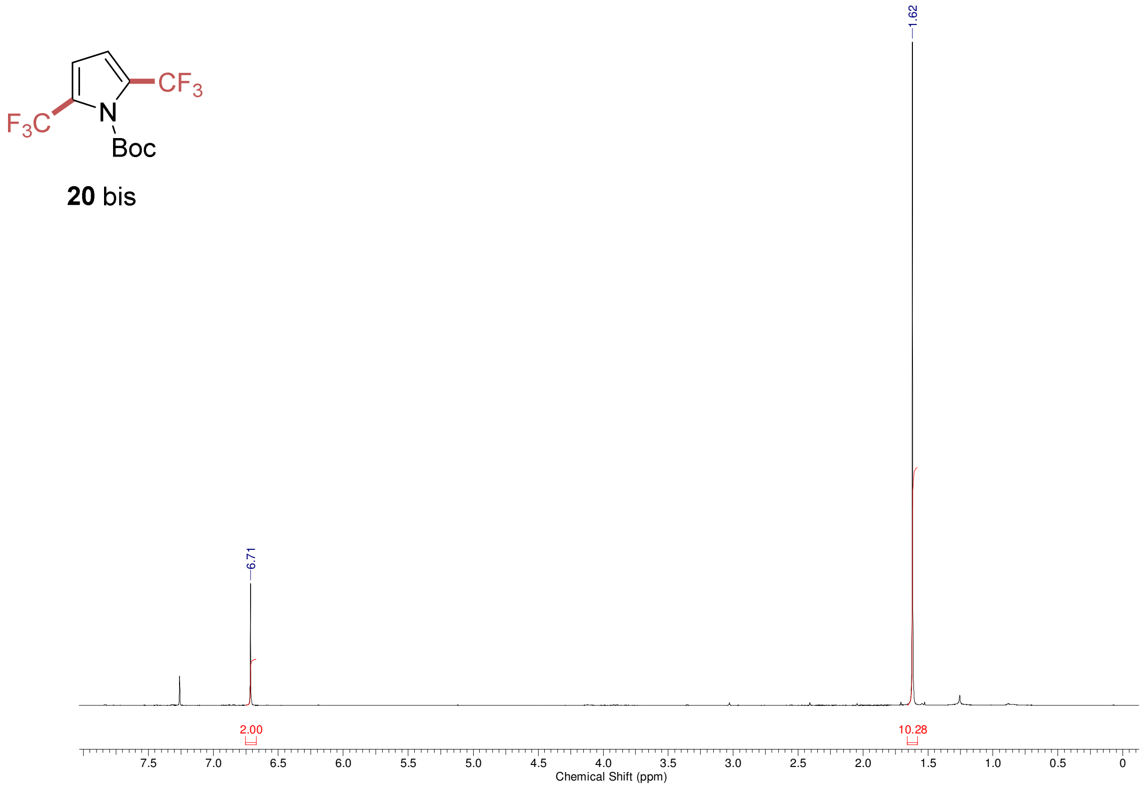

20 bis

Supplementary Figure 36 | ^{1}H NMR spectrum of *tert*-butyl 2,5-bis(trifluoromethyl)-1*H*-pyrrole-1-carboxylate. (500 MHz, $CDCl_3$, 298 K).

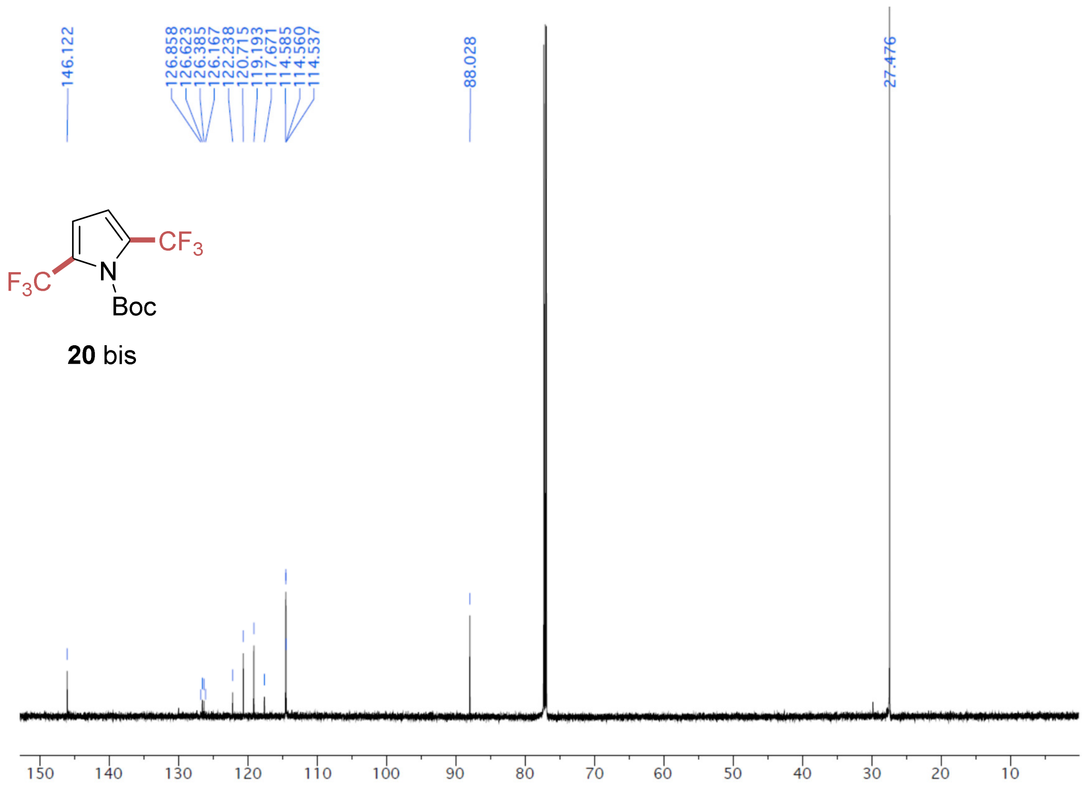

Supplementary Figure 37 | ^{1}H NMR spectrum of *tert*-butyl 2,5-bis(trifluoromethyl)-1*H*-pyrrole-1-carboxylate. (175 MHz, $CDCl_3$, 298 K).

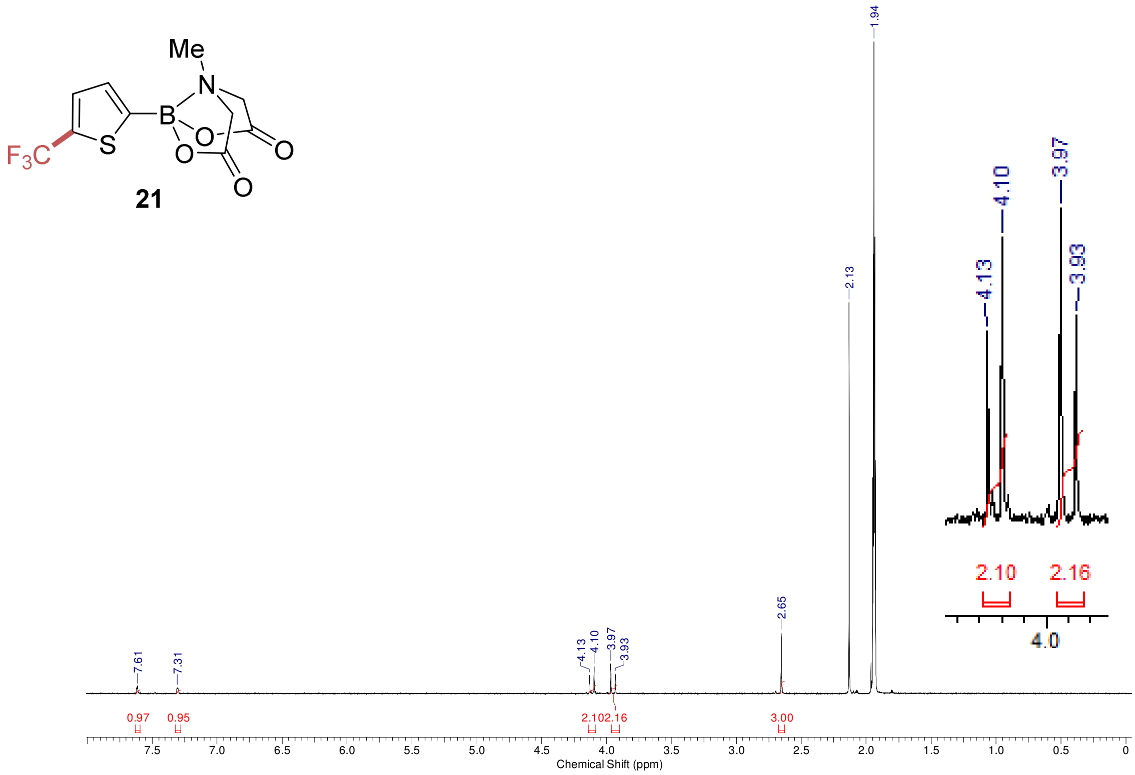

Supplementary Figure 38 | ^{1}H NMR spectrum of (5-(trifluoromethyl)thiophen-2-yl)boronic acid MIDA ester. (400 MHz, CD_3CN, 298 K).

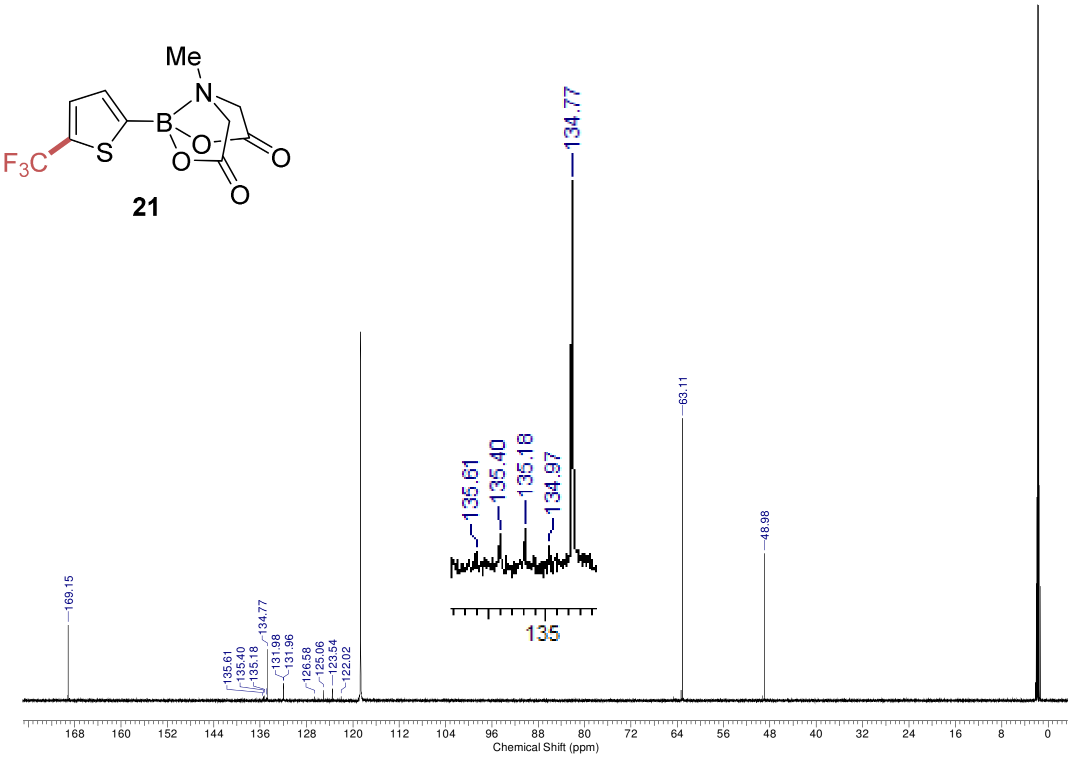

Supplementary Figure 39 | ^{13}C NMR spectrum of (5-(trifluoromethyl)thiophen-2-yl)boronic acid MIDA ester. (175 MHz, CD_3CN, 298 K).

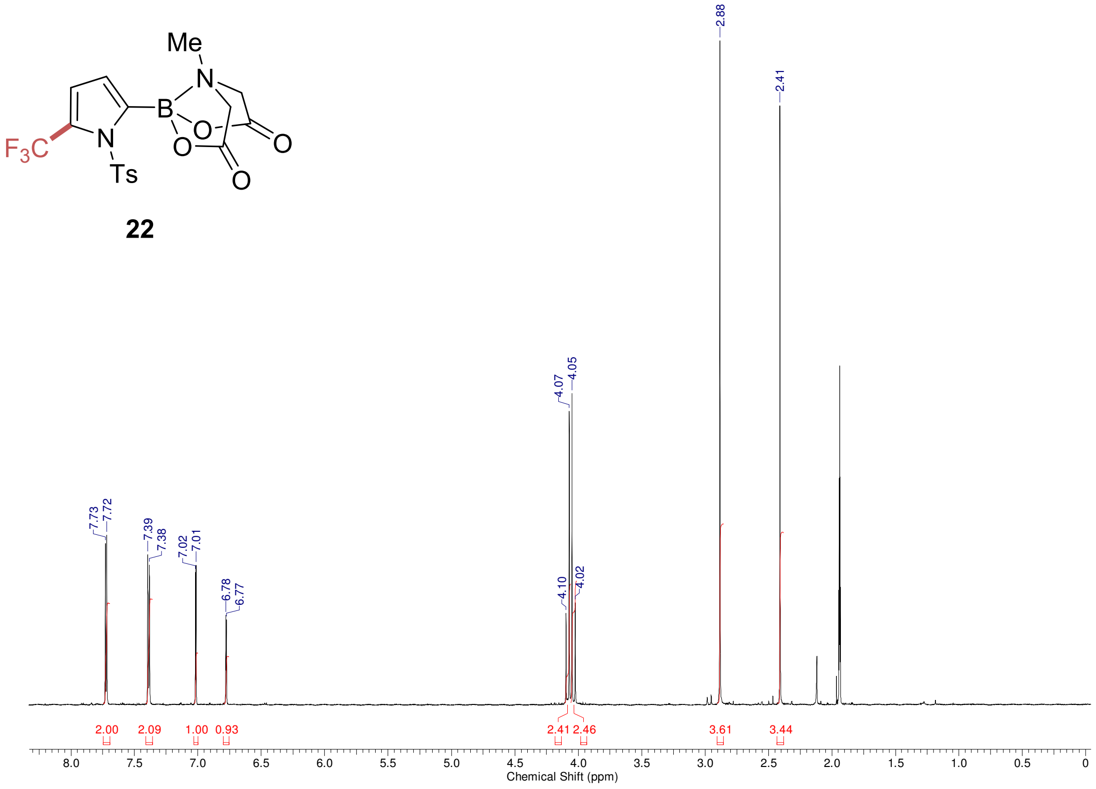

22

Supplementary Figure 40 | ^{1}H NMR spectrum of (1-tosyl-5-(trifluoromethyl)-1*H*-pyrrol-2-yl)boronic acid MIDA ester. (700 MHz, CD_3CN, 298 K).

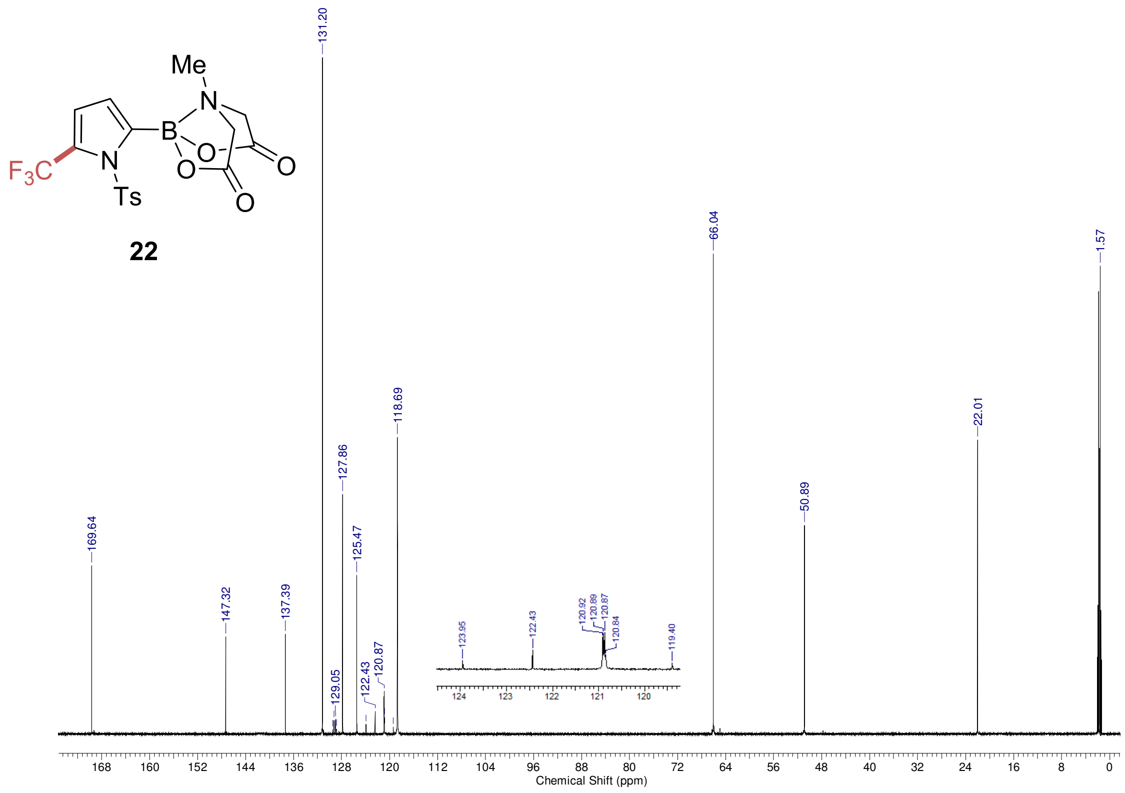

Supplementary Figure 41 | ^{13}C NMR spectrum of (1-tosyl-5-(trifluoromethyl)-1*H*-pyrrol-2-yl)boronic acid MIDA ester. (175 MHz, CD_3CN, 298 K).

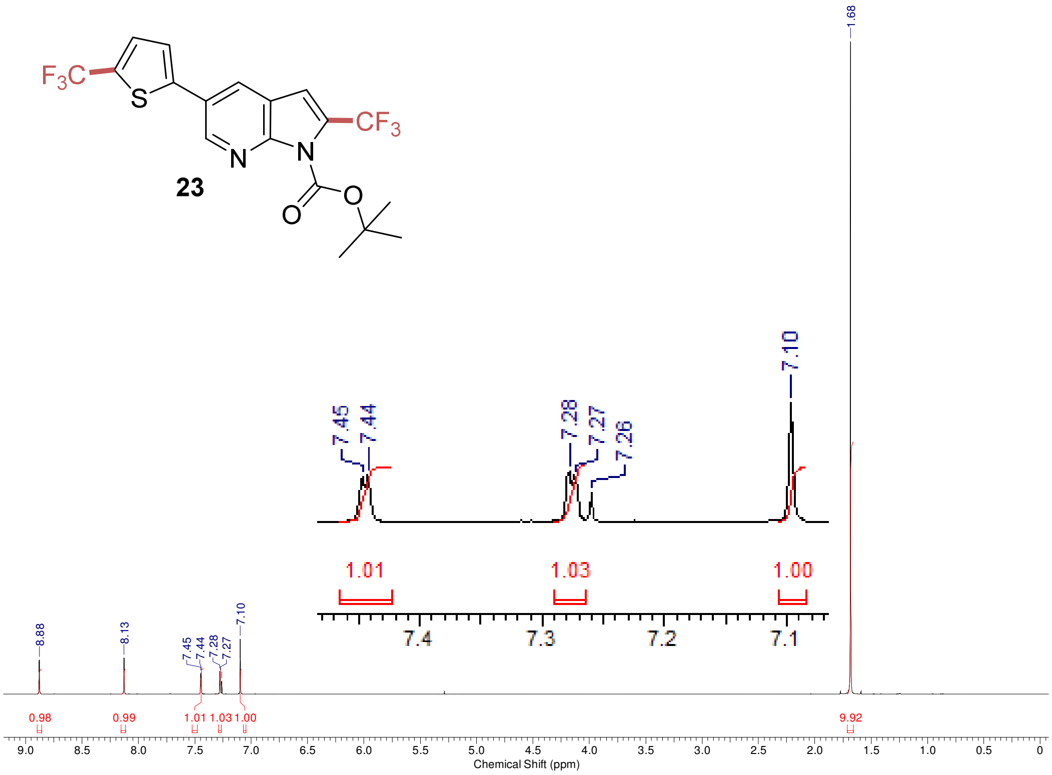

Supplementary Figure 42 | ^{1}H NMR spectrum of *tert*-butyl 2-(trifluoromethyl)-5-(5-(trifluoromethyl)thiophen-2-yl)-1*H*-indole-1-carboxylate. (700 MHz, $CDCl_3$, 298 K).

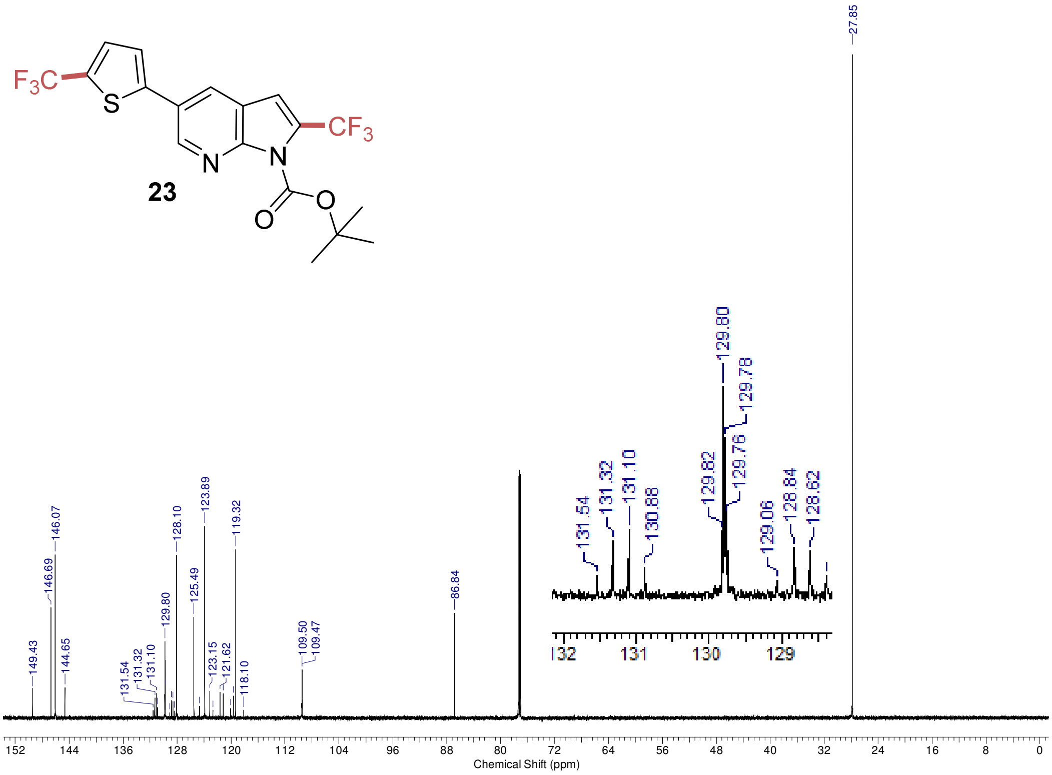

Supplementary Figure 43 | ^{13}C NMR spectrum of *tert*-butyl 2-(trifluoromethyl)-5-(5-(trifluoromethyl)thiophen-2-yl)-1*H*-indole-1-carboxylate. (700 MHz, CD_3CN, 298 K).

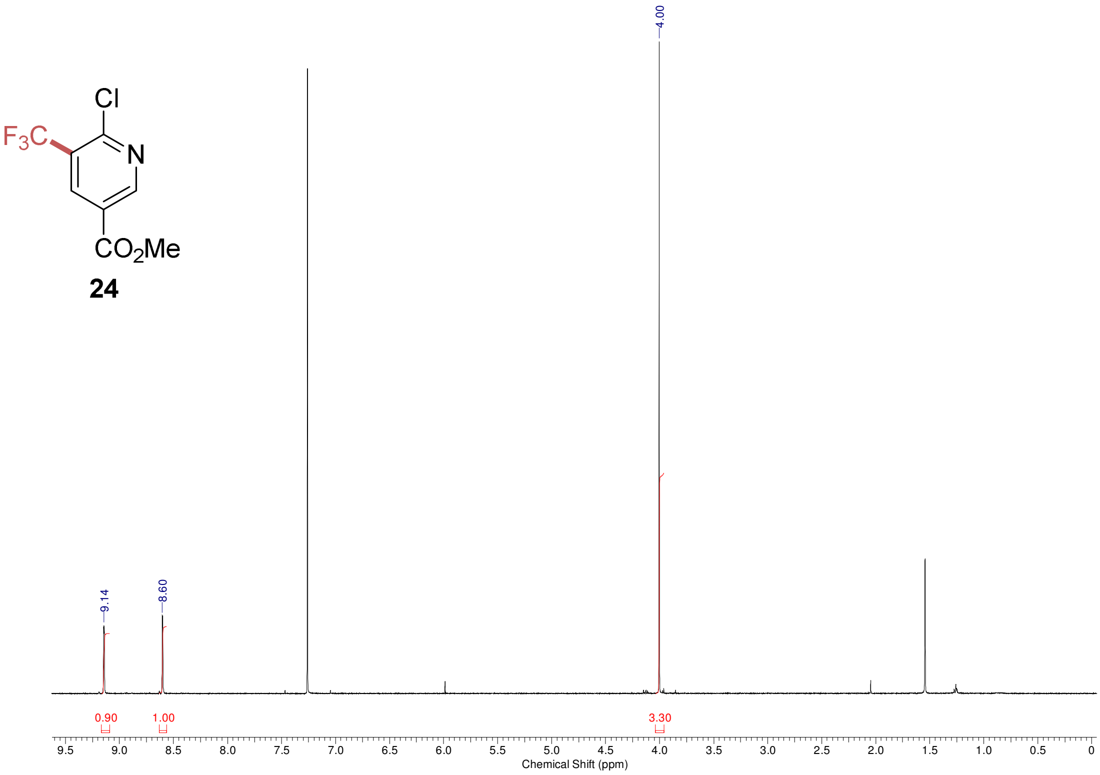

Supplementary Figure 44 | ^{1}H NMR spectrum of methyl 6-chloro-5-(trifluoromethyl)nicotinate. (500 MHz, $CDCl_3$, 298 K).

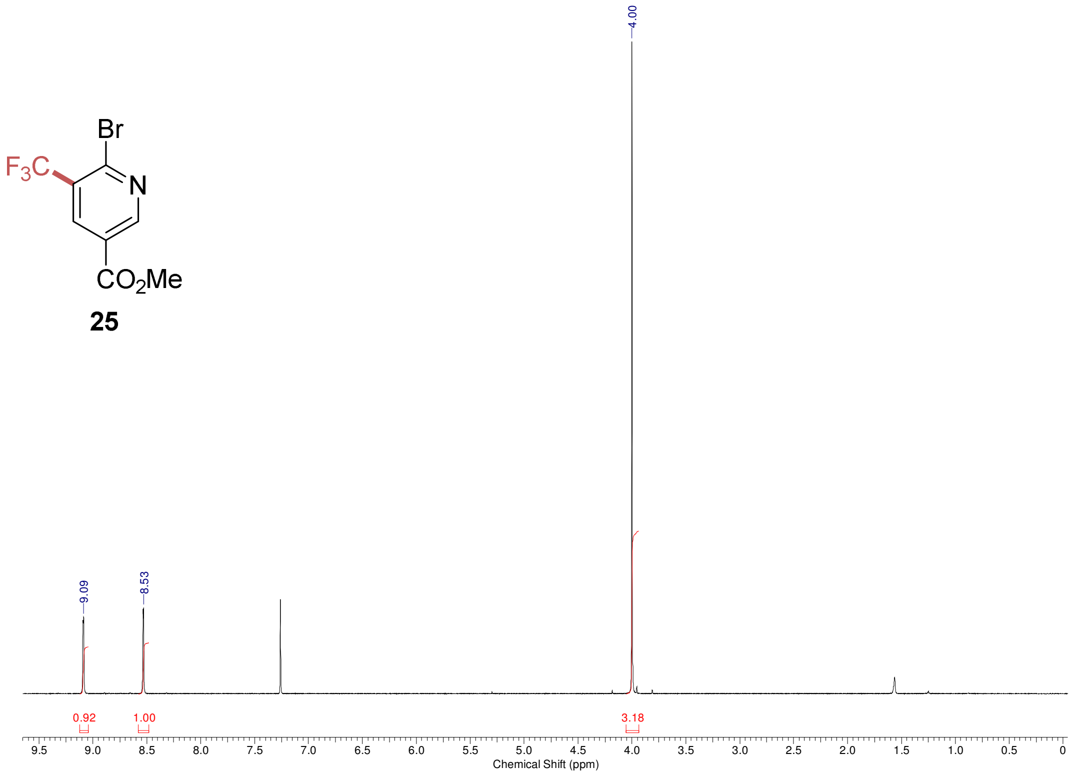

Supplementary Figure 45 | ^{1}H NMR spectrum of methyl 6-bromo-5-(trifluoromethyl)nicotinate. (500 MHz, $CDCl_3$, 298 K).

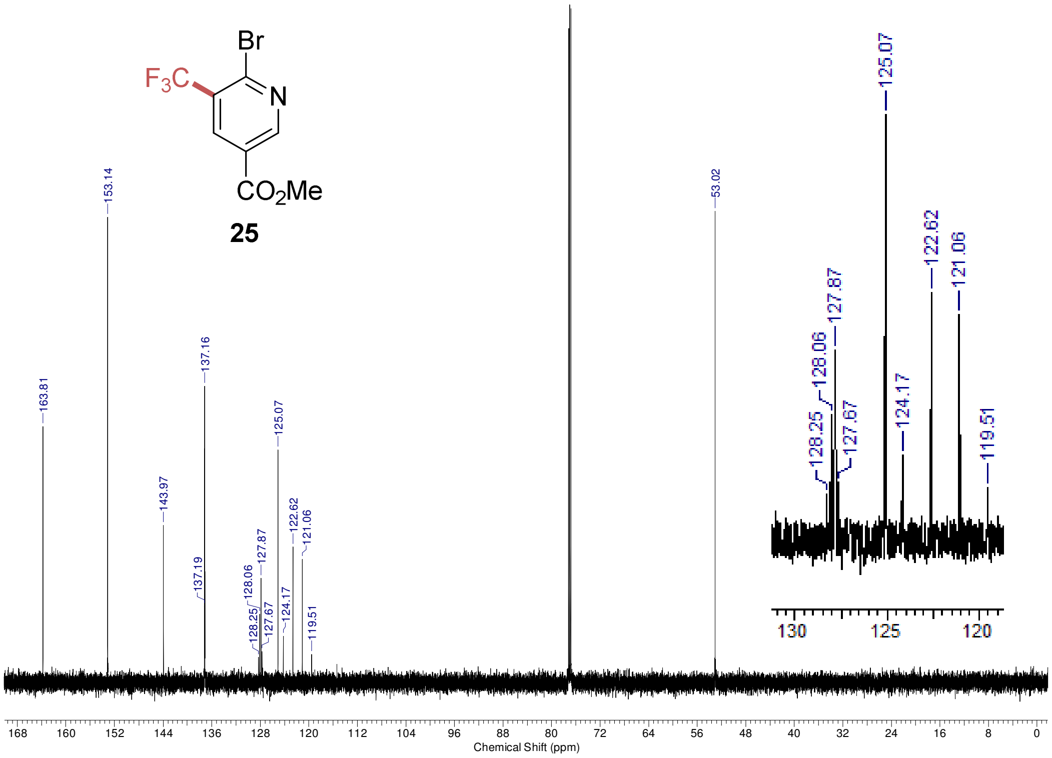

Supplementary Figure 46 | ^{13}C NMR spectrum of methyl 6-bromo-5-(trifluoromethyl)nicotinate. (175 MHz, $CDCl_3$, 298 K).

Supplementary Tables

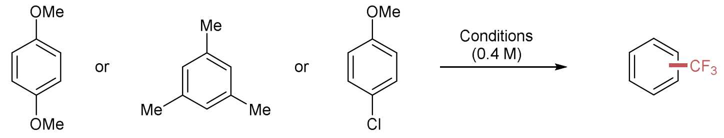

A B C

Entry	Substrate[a]	Solvent	Catalyst (1 mol%)	pyridine-*N*-oxide (equiv.)	TFAA (equiv.)	Time (h)	Yield (%)[b]
1	**A**	CH_2Cl_2	Ir(ppy)$_2$(dtbbpy)PF_6	2[c]	2	24	39%
2	**A**	CH_2Cl_2	Ir(ppy)$_2$(dtbbpy)PF_6	2	2	24	39%
3	**B**	CH_2Cl_2	Ir(ppy)$_2$(dtbbpy)PF_6	2	2	12	62% (56:6)[d]
4	**C**	CH_2Cl_2	Ir(ppy)$_2$(dtbbpy)PF_6	2	4	24	33% (2:1)[e]
5	**C**	CH_2Cl_2	Ir(ppy)$_2$(dtbbpy)PF_6	2	4	24	37% (2:1)[e]
6	**C**	CH_2Cl_2	Ru(phen)$_3$$Cl_2$	2	4	24	15%
7	**C**	CH_2Cl_2	Ru(bpy)$_3$$Cl_2$	2	4	24	38% (2:1)[e]
8	**C**	CH_2Cl_2	Ru(bpy)$_3$$Cl_2$	1	1.1	15	33%
9[f]	**C**	CH_2Cl_2	Ru(bpy)$_3$$Cl_2$	1	1.1	15	34%
11	**C**	MeCN	Ru(bpy)$_3$$Cl_2$	2	4	15	42% (2:1)[e]
12	**C**	MeCN	Ru(bpy)$_3$$Cl_2$	2	4	45	55% (2:1)[e]
13	**C**	MeCN	Ru(bpy)$_3$$Cl_2$	4	8	15	51% (2:1)[e]
14	**B**	MeCN	Ir(ppy)$_2$(dtbbpy)PF_6	1	1.1	15	48% (45:3)[d]
15	**B**	MeCN	Ir(dF(CF3)ppy)$_2$(dtbbpy)PF_6	1	1.1	15	32% (31:1)[d]
16	**B**	MeCN	Ru(bpy)$_3$$Cl_2$	1	1.1	15	52% (48:4)[d]
17	**B**	MeCN	9-Mesityl-10-methylacridinium	1	1.1	15	7%
18	**B**	MeCN	Eosin Y (5 mol%)	1	1.1	15	0%
19	**B**	CH_2Cl_2	Ru(bpy)$_3$$Cl_2$	1	1.1	15	50% (47:3)[d]
20	**B**	DMF	Ru(bpy)$_3$$Cl_2$	1	1.1	15	25%
21	**B**	MeCN	Ru(bpy)$_3$$Cl_2$ (0.1 mol%)	1	1.1	15	51% (48:4)[d]
22	**B**	MeCN	none	1	1.1	15	0%
23[g]	**B**	MeCN	Ru(bpy)$_3$$Cl_2$	1	1.1	15	0%
24	**B**	MeCN	Ru(bpy)$_3$$Cl_2$	none	1.1	15	0%
25	**B**	MeCN	Ru(bpy)$_3$$Cl_2$	2	2.1	15	**69% (59:10)**[d]
26	**B**	MeCN	Ru(bpy)$_3$$Cl_2$	3	3.1	15	71% (49:22)[d]

[a]Run on 0.8 mmol scale. [b]FNMR yields vs. 1 equivalent of trifluorotoluene as internal standard. [c]Dry, lyophilized pyridine-*N*-oxide used. [d]Mono-:di-functionalized product ratio. [e]Regioisomeric ratio. [f]Degassed (freeze-pump-thaw x 3) [g]Light excluded.

Supplementary Table 1| Select Optimization and Control Experiments

Supplementary Notes

Supplementary Note 1:

Crude Reagent Cost Comparison

For a peer-reviewed cost comparison of some trifluoromethylation reagents, see: McReynolds, K. A. *et al.* J. Fluorine Chem. 131, 1108–1112 (2010), the relevant results of which are summarized here. The calculations in this particular paper were performed based on the largest quantity available in the 2009-2010 Aldrich catalogue.

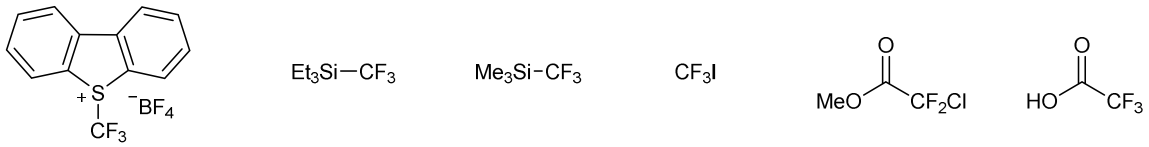

$23,160/mol $11,790/mol $2,254/mol $755.2/mol $315.0/mol $23.70/mol

CF_3 source cost comparisons from McReynolds, K. A. et al. J. Fluorine Chem. 131, 1108–1112 (2010)

In this vein, as there is no up-to-date and direct comparison available concerning many popular and effective CF_3 sources, we have compiled a price-per-mole comparison of a select number of CF_3 sources from the Aldrich website (see next page). These prices are extrapolated (or, in the case of TFAA, TFA, and CF_3H, interpolated) from the prices for the largest available quantities listed. It should be noted that these price comparisons are not strictly fair or accurate, as the largest quantity available for each material differs substantially; furthermore, although material availability *should* be factored into an analysis of which trifluromethylating reagents are the most useful on scale, we recognize that the Aldrich catalogue pricing will most likely not be an accurate reflection of material availability. Therefore for certain reagents we also include pricing based on sources available to Eli Lilly and Co. for moderate scale 0.5-5 Kg and projected estimates at larger scale (1000 Kg).

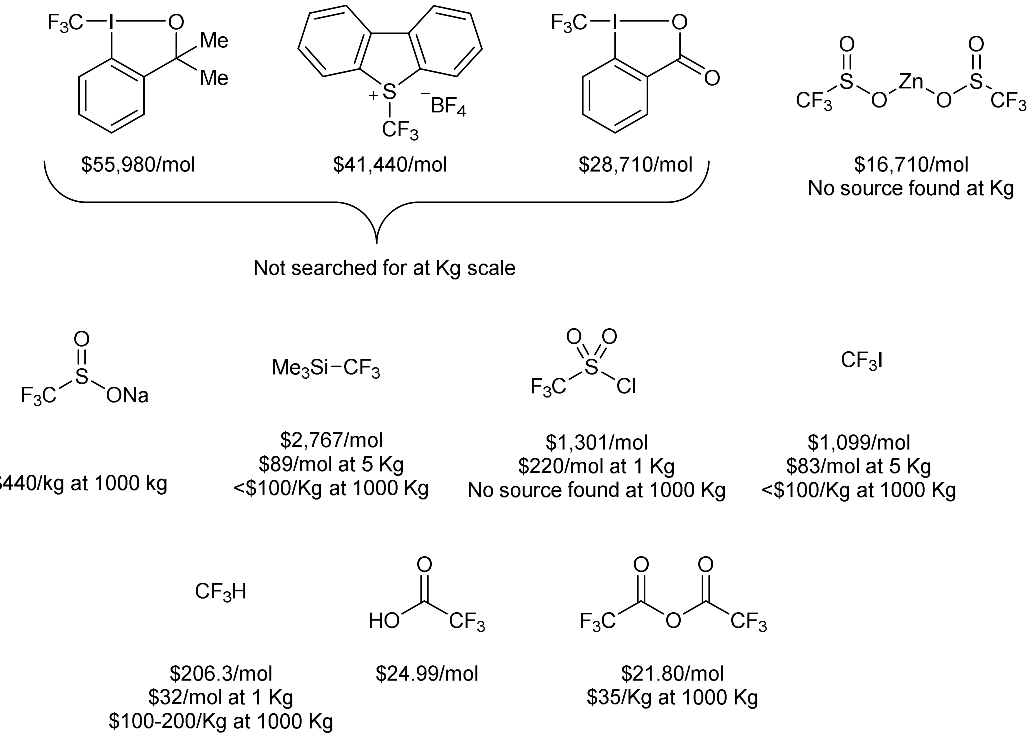

CF_3 source cost comparison from the Sigma Aldrich website and from sources available to Eli Lilly and Co, as of April 2015. Price-per-mole values were extrapolated or interpolated from the largest quantity available for each reagent.

Supplementary Methods

General information & Reaction Apparatus:

All chemicals were used as received. Reactions were monitored by TLC and visualized with a dual short wave/long wave UV lamp. Column flash chromatography was performed using 230-400 mesh silica gel or via automated column chromatography. Preparative TLC purifications were run on silica plates of 1000 μm thickness. NMR spectra were recorded on Varian MR400, Varian Inova 500, Varian Vnmrs 500, or Varian Vnmrs 700 spectrometers. Chemical shifts for ^{1}HNMR were reported as δ, parts per million, relative to the signal of $CHCl_3$ at 7.26 ppm. Chemical shifts for ^{13}CNMR were reported as δ, parts per million, relative to the center line signal of the $CDCl_3$ triplet at 77.0 ppm. Chemical shifts for ^{19}FNMR were reported as δ, parts per million, relative to the signal of a trifluorotoluene internal standard at -63.72 ppm. The abbreviations s, br. s, d, dd, br. d, ddd, t, q, br. q, qi, m, and br. m stand for the resonance multiplicity singlet, broad singlet, doublet, doublet of doublets, broad doublet, doublet of doublet of doublets, triplet, quartet, broad quartet, quintet, multiplet and broad multiplet, respectively. IR spectra were recorded on a Perkin-Elmer Spectrum BX FT-IR spectrometer fitted with an ATR accessory. Mass Spectra were recorded at the Mass Spectrometry Facility at the Department of Chemistry of the University of Michigan in Ann Arbor, MI on an Agilent Q-TOF HPCL-MS with ESI high resolution mass spectrometer. LED lights and the requisite power box and cables were purchased from Creative Lighting Solutions (http://www.creativelightings.com) with the following item codes: CL-FRS5050-12WP-12V (4.4W blue LED light strip), CL-FRS5050WPDD-5M-12V-BL (72 W LED strip), CL-PS94670-25W (25 W power supply), CL-PS16020-150W (150 W power supply), CL-PC6FT-PCW (power cord), CL-TERMBL-5P (terminal block). A reaction performed with a 24 W CFL placed 5 cm from the vial provided identical results.

Unless stated otherwise, all reactions were run on a 0.8mmol scale in a 2 dram vial equipped with stir bar and septum. The light apparatuses used to irradiate the reactions were constructed from test tube racks and wrapped with three 4W LED strips. Reactions were run only in slots marked by an X in the picture below so as to keep a moderate distance from the light source (~2.5 cm). At this distance the temperature of the reactions did not exceed 35 ˚C.

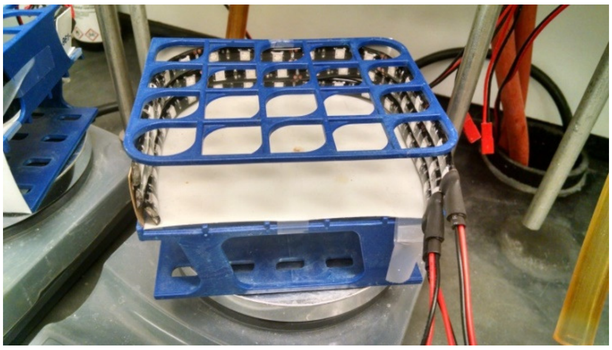

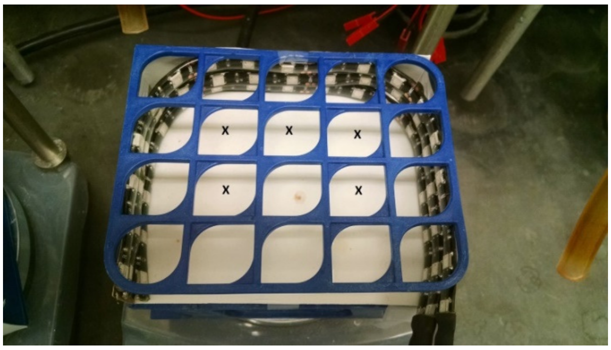

Synthesis of Compounds 1-22:

Optimized Trifluoromethylation Procedure:
For most reported substrates, both procedures 1 and 2 were performed. The reaction mixtures obtained by superior conditions (as judged by lack of byproduct formation in preference to higher yields) were then purified as described. In select circumstances, the reactions were amenable to the use of more equivalents of pyridine-*N*-oxide and TFAA (procedure 3).

Procedure 1:
To a 2 dram vial equipped with a stir bar was added pyridine *N*-oxide (76 mg, 0.80 mmol, 1.0 equiv), $Ru(bpy)_3Cl_2•6H_2O$ (6.0 mg, 1.0 mol%), and substrate (0.80 mmol). The combined materials were then dissolved in MeCN (2.0 ml) and stirred to form a homogeneous solution. Trifluoroacetic anhydride (120 µl, 190 mg, 0.88 mmol, 1.1 equiv) was then added to the resulting solution. The vial was equipped with a screw-on cap with septum, and a 25 gauge needle was placed through the septum for the duration of the reaction. Three 4.4 W LED light strips (positioned 2.5 cm away) were turned on and the reaction was allowed to run for 12-15 hours before the light source was removed. Trifluorotoluene (98 µl, 0.80 mmol) was added as a stoichiometric internal standard. A sample of the reaction was removed and diluted with $CDCl_3$ for NMR analysis. The trifluorotoluene signal was referenced to -63.72 ppm. Workup was performed by diluting the reaction with CH_2Cl_2 and washing with 1N HCl, followed by saturated $NaHCO_3$ and then brine. The organic layer was dried over sodium sulfate before filtering and concentrating at 40 ˚C under reduced pressure.

Procedure 2:
Procedure 2 is identical to Procedure 1 except 152 mg of pyridine-*N*-oxide (1.6 mmol, 2.0 equiv) and 237 µl of trifluoroacetic anhydride (353 mg, 1.68 mmol, 2.1 equiv) were used.

Procedure 3:
Procedure 2 is identical to Procedure 1 except 304 mg of pyridine-*N*-oxide (3.2 mmol, 4.0 equiv) and 902 µl of trifluoroacetic anhydride (1.34 g, 6.4 mmol, 8.0 equiv) were used, and the reaction was run for 24 hours.

Substrates:

1,4-dimethoxy-2-(trifluoromethyl)benzene:[1] **(1)**

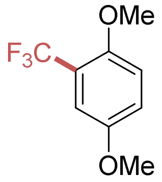

Procedure 1: 26% yield (FNMR)
Procedure 2: 45% yield (FNMR)
The reaction was run according to **Procedure 2** and was purified by column chromatography (0% to 15% CH_2Cl_2 in hexanes) to afford the title compound (51 mg, 21%, volatile) and remaining starting material (17 mg, 10%). The acquired ^{1}HNMR spectrum as identical to that reported in the literature; ^{1}HNMR ($CDCl_3$, 500 MHz): δ 7.12 (d, J = 2.8 Hz, 1H), 7.02 (dd, J = 9.1, 2.8 Hz, 1H), 6.94 (d, J = 9.1 Hz, 1H), 3.86 (s, 3H), 3.80 (s, 3H);

methyl 2-(trifluoromethyl)benzoate,[2] methyl 3-(trifluoromethyl)benzoate[3] and methyl 4-(trifluoromethyl)benzoate[4] (2)

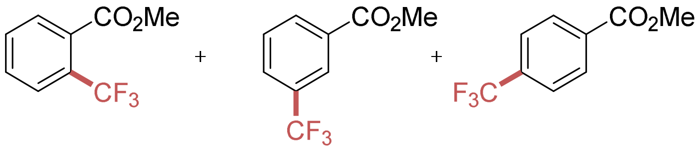

Procedure 1: 24% yield (FNMR, 13:4:7, o:m:p)

Procedure 2: 44% yield (FNMR, 23:8:13, o:m:p)

The reaction was run according to **Procedure 2** and was purified by preparative TLC (1:1 CH_2Cl_2:hexanes) to yield the inseparable isomers as a clear oil (37 mg, 23%) in a 4.2:1.0:1.7 ratio (o:m:p). The ^{1}HNMR of the obtained mixture matched each of the known products. ^{1}HNMR ($CDCl_3$, 500 MHz): δ 8.31 (s, 1H, *meta*), 8.24 (d, *J* = 7.5 Hz, 1H, *meta*), 8.16 (d, *J* = 8.4 Hz, 2H, *para*), 7.82 (d, *J* = 7.8 Hz, 1H, *meta*), 7.79-7.78 (complex m, 1H, *ortho*), 7.76-7.75 (complex m, 1H, *ortho*), 7.71 (d, *J* = 8.4 Hz, 2H, *para*), 7.63-7.58 (m, overlap, 2H *ortho*, 1H *meta*), 3.95 (s, 3H, *meta*), 3.95 (s, 3H, *para*), 3.93 (s, 3H, *ortho*).

methyl 4-methoxy-2-(trifluoromethyl)benzene and methyl 4-methoxy-3-(trifluoromethyl)benzene (3)

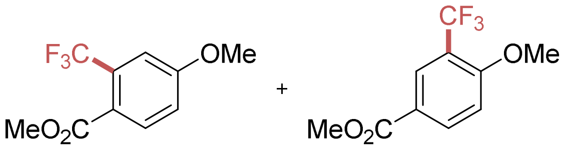

The reaction was run according to **Procedure 3**. Upon completion, the reaction was worked up and purified by preparative TLC (100% CH_2Cl_2) to provide the title compounds in a 2:3 regioisomeric mixture (90.3 mg, 48%) along with recovered starting material (35.1 mg, 26%) and methyl 4-methoxy-3,6-dioxocyclohexa-1,4-diene-1-carboxylate (10.3 mg, 7%). The product regioisomers were characterized as a mixture:

^{1}HNMR ($CDCl_3$, 500 MHz): δ 8.27 (d, *J* = 2.2 Hz, 1H, *major*), 8.20 (dd, *J* = 8.5, 2.2 Hz, 1H, *major*), 7.86 (d, *J* = 8.7 Hz, 1H, *minor*), 7.25 (d, *J* = 2.3 Hz, 1H, *minor*), 7.06 (dd, *J* = 8.7, 2.3 Hz, 1H, *minor*), 7.04 (d, *J* = 8.5 Hz, 1H, *major*); ^{13}CNMR ($CDCl_3$, 125 MHz): δ 166.5, 165.8, 161.7, 160.9, 135.1, 133.1, 131.0 (q, J_{CF} = 32.4 Hz), 129.0 (q, J_{CF} = 4.8 Hz), 123.1 (q, J_{CF} = 271.8 Hz), 123.0 (q, J_{CF} = 273.7), 122.5 (q, J_{CF} = 1.9 Hz), 122.1, 118.7 (q, J_{CF} = 31.5 Hz), 115.8, 113.3 (q, J_{CF} = 6.0 Hz), 111.5, 56.2, 55.7, 52.5, 52.2; ^{19}FNMR ($CDCl_3$, 465 MHz): δ -60.90 (*minor*), -63.89 (*major*); IR (neat): 3106, 3018, 2963, 2849, 1728, 1707, 1618, 1582, 1509, 1424, 1264, 1236, 1138, 1115, 1016; HRMS (ESI) *m/z* calculated for $C_{10}H_{10}F_3O_3$ ([M+H]+) 235.0577, found 235.0574.

(trifluoromethyl)benzene (4)

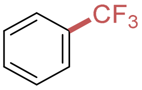

Procedure 1: 25% yield (FNMR)

Procedure 1 (10 equiv benzene): 45% yield (FNMR)

Each reaction for this substrate was quenched with methanol before adding trifluoroethanol as an internal standard (0.8 mmol, 58 μl). Due to the wide availability of the product, no purification was attempted on this reaction mixture. The proton and fluorine signals of the product were identical to those of a commercial sample.

4,6-dimethoxy-5-(trifluoromethyl)pyrimidine[5] (5)

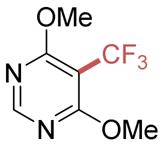

Procedure 1: 23% yield (FNMR)
Procedure 2: 37% yield (FNMR)
The reaction was run according to **Procedure 2** and purified by preparative TLC (10% EtOAc in hexanes) to provide the title compound as a white solid (50 mg, 30%) along with recovered starting material (14 mg, 13%). The acquired ^{1}HNMR spectrum matched that reported in the literature; ^{1}HNMR ($CDCl_3$, 500 MHz): δ 8.47 (s, 1H), 4.04 (s, 6H);

methyl 1-methyl-6-oxo-5-(trifluoromethyl)-1,6-dihydropyridine-3-carboxylate (6)

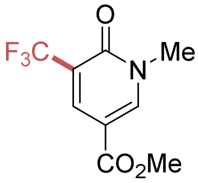

Procedure 1: 38% yield (FNMR)
Procedure 2: 48% yield (FNMR)
The reaction was run according to **Procedure 2** and was concentrated after 15 hours without workup. The crude residue was purified on SiO_2 (5% to 40% EtOAc) to provide the title compound (100 mg, 54%) as an off-white solid. Remaining starting material (8.0 mg, 6%) was eluted off the column in 65% EtOAc in hexanes. ^{1}HNMR ($CDCl_3$, 500 MHz): δ 8.37 (d, J = 2.3 Hz, 1H), 8.28 (d, J = 2.3 Hz, 1H), 3.90 (s, 3H), 3.66 (s, 3H); ^{13}CNMR ($CDCl_3$, 175 MHz): δ 163.6, 158.5, 146.6, 138.1 (q, J_{CF} = 5.2 Hz), 122.1 (q, J_{CF} = 271.1 Hz), 119.1 (q, J_{CF} = 31.4 Hz), 107.8, 52.3, 38.3; ^{19}FNMR ($CDCl_3$, 465 MHz): δ -67.23; IR (neat): 3066, 2965, 1717, 1671, 1560, 1448, 1255, 1122; HRMS (ESI) *m/z* calculated for $C_9H_9F_3NO_3$ ($[M+H]^+$) 236.0529, found 236.0529;

2,2,2-trifluoro-1-(1-phenyl-5-(trifluoromethyl)-1H-pyrrol-2-yl)ethan-1-one: **(7)**

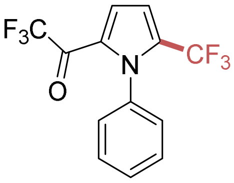

Procedure 1: 39% yield (FNMR)
Procedure 2: 53% yield (FNMR)
The reaction mixture from **Procedure 2** was purified by preparative TLC (10% EtOAc in hexanes) to afford the title compound (200 mg, 49%) as a clear oil along with recovered starting material (20 mg, 10%). ^{1}HNMR (MeCN-d_3, 500 MHz): δ 7.58-7.50 (m, overlap, 3H), 7.40-7.36 (m, overlap, 3H), 6.97 (d, J = 4.6 Hz, 1H); ^{13}CNMR ($CDCl_3$, 175 MHz): δ 170.2 (q, J_{CF} = 36.8 Hz), 136.5, 132.2 (q, J_{CF} = 38.1 Hz), 129.8, 129.0, 127.5, 125.5, 121.4 (q, J_{CF} = 4.1 Hz), 119.7 (q, J_{CF} = 269.8 Hz), 116.3 (q, J_{CF} = 290.9 Hz), 112.3 (q, J_{CF} = 3.2 Hz); ^{19}FNMR ($CDCl_3$, 470 MHz): δ -58.93, -73.30; IR (neat): 3149, 1705, 1502, 1346, 1202, 1123; HRMS (EI) *m/z* calculated for $C_{13}H_8F_6NO$ ($[M+H]^+$) 307.0432, found 307.0440.

***tert*-butyl 2-(trifluoromethyl)-1*H*-indole-1-carboxylate:[6] (8)**

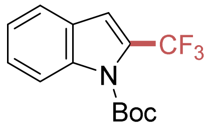

Procedure 1: 26% yield (FNMR)
Procedure 2: 19% yield (FNMR)
The reaction was run according to **Procedure 1** and purified on SiO_2 (100% hexanes) to afford the title compound (58 mg, 25%) as a clear oil, along with recovered starting material (26 mg, 15%). The acquired ^{1}HNMR spectrum was identical to that reported in the literature; ^{1}HNMR ($CDCl_3$, 500MHz): δ 8.29 (d, J = 8.1 Hz, 1H), 7.62 (d, J = 7.7 Hz, 1H), 7.45 (dd, J = 8.1, 7.7 Hz, 1H), 7.30 (dd, J = 7.7, 7.7 Hz, 1H), 7.14 (s, 1H), 1.68 (s, 9H);

3-methyl-2-(trifluoromethyl)benzofuran:[7] (9)

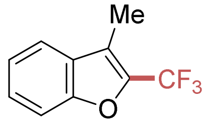

Procedure 1: 47% yield (FNMR)
The reaction was run according to Procedure 1. The crude reaction mixture was purified by preparative TLC (100% hexanes) and the title compound was isolated as a clear oil (39 mg, 37%). The acquired ^{1}HNMR spectrum matched that reported in the literature; ^{1}HNMR ($CDCl_3$, 500 MHz): δ 7.61 (d, J = 7.7 Hz, 1H), 7.52 (d, J = 8.2 Hz, 1H), 7.43 (dd, J = 7.7, 7.7 Hz, 1H), 7.33 (dd, J = 7.7, 7.7 Hz, 1H) 2.41 (q, $J_{H\text{–}F}$ = 2.0 Hz, 3H);

5-(4-bromophenyl)-2-(trifluoromethyl)oxazole and 5-(4-bromophenyl)-4-(trifluoromethyl)oxazole: (10)

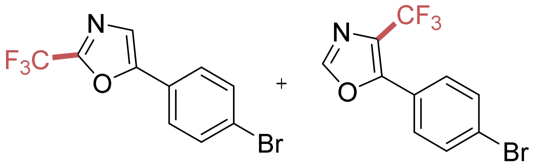

Procedure 1: 27% (FNMR) ~1:1 regioisomeric ratio
Procedure 2: 26% (FNMR) ~1:1 regioisomeric ratio
The reaction was run according to **Procedure 1** and purified on SiO_2 (1-5% EtOAc in hexanes) to provide 5-(4-bromophenyl)-2-(trifluoromethyl)oxazole (30 mg, 10%) and 5-(4-bromophenyl)-4-(trifluoromethyl)oxazole (22 mg, 7%) as white solids. In addition, starting material was recovered (100 mg, 45%) along with 1-(5-4-bromophenyl)oxazol-4-yl)-2,2,2-trifluoroethan-1-one (38 mg, 12%).

5-(4-bromophenyl)-2-(trifluoromethyl)oxazole: ^{1}HNMR ($CDCl_3$, 700 MHz): δ 7.61 (d, J = 8.8 Hz, 2H), 7.57 (d, J = 8.8 Hz, 2H), 7.46 (s, 1H); ^{13}CNMR ($CDCl_3$, 175 MHz): δ 152.9, 150.0 (q, J_{CF} = 43.6 Hz), 132.4, 126.3, 125.1, 124.1, 122.8, 116.4 (q, J_{CF} = 270.4 Hz); ^{19}FNMR ($CDCl_3$ 470 MHz): δ -66.66; IR (neat): 3146, 1677, 2587, 2478, 1406, 1202, 1137; HRMS (EI) *m/z* calculated for $C_{10}H_6BrF_3NO$ ($[M+H]^+$) 291.9579, found 291.9574.

5-(4-bromophenyl)-4-(trifluoromethyl)oxazole: ^{1}HNMR ($CDCl_3$, 700 MHz): 7.96 (s, 1H), 7.64 (d, J = 8.6 Hz, 2H), 7.55 (d, J = 8.6 Hz, 2H); ^{13}CNMR ($CDCl_3$, 175 MHz): δ 150.0 (q, J_{CF} = 3.2 Hz), 149.9, 132.2, 129.1 (q, J_{CF} = 2.0 Hz), 125.8 (q, J_{CF} = 39.5 Hz), 125.1, 124.6, 121.0 (q, J_{CF} = 268.4 Hz); ^{19}FNMR ($CDCl_3$ 470 MHz): δ -61.78;
IR (neat): 3121, 1678, 1617, 1586, 1514, 1489, 1384, 1125; HRMS (EI) *m/z* calculated for $C_{10}H_5BrF_3NO$ ($[M]^+$) 290.9507, found 290.9516.

2-bromo-1,3,5-trimethyl-4-(trifluoromethyl)benzene: (11)

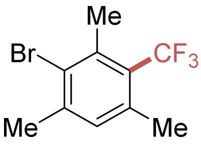

Procedure 1: 35% yield (FNMR)
Procedure 2: 48% yield (FNMR) with 4% yield of the double-addition product
Procedure 3: 65% yield (FNMR) with a 5:1 ratio of mono:di functionalized products

The reaction mixture from **Procedure 1** was purified by preparative TLC (100% hexanes) to afford 2-bromo-1,3,5-trimethyl-4-(trifluoromethyl)benzene as a clear oil (44 mg, 22%) along with 37 mg (23%) of recovered starting material; ^{1}HNMR ($CDCl_3$, 500 MHz): δ 6.96 (s, 1H), 2.57 (q, J_{HF} = 2.4 Hz, 3H), 2.43-2.40 (m, overlap, 6H); ^{13}CNMR ($CDCl_3$, 175 MHz): δ 141.6, 137.6, 135.6, 132.1, 127.6, 126.7 (q, J_{CF} = 28.6 Hz), 125.4 (q, J_{CF} = 276.6 Hz), 24.4, 21.7 (q, J_{CF} = 4.8 Hz), 21.5 (q, J_{CF} = 4.5 Hz); ^{19}FNMR ($CDCl_3$, 465 MHz): δ -54.10; IR (neat): 2983, 1380, 1283, 1225, 1153, 1112; HRMS (EI) *m/z* calculated for $C_{10}H_{11}BrF_3$ ($[M+H]^+$) 265.9918, found 265.9925.

2-bromo-1,3-dimethyl-4-(trifluoromethyl)benzene:[7] (12)

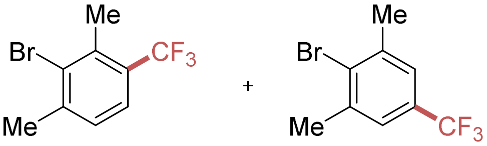

Procedure 1: 25% yield (FNMR, 5:1 regioisomeric mixture)
Procedure 2: 34% yield (FNMR, 5:1 regioisomeric mixture)
Procedure 3: 46% yield (FNMR, 5:1 regioisomeric mixture)

The reaction mixture from **Procedure 2** was purified by preparative TLC (100% hexanes) to afford the title compound in a 4:1 regioisomeric mixture (32 mg, 16%) along with 12 mg (8%) of recovered starting material. We were unable to separate the product regioisomers so they were characterized as a mixture; ^{1}HNMR ($CDCl_3$, 500 MHz): δ 7.48 (d, J = 8.1 Hz, 1H, *major*), 7.32 (s, 2H, *minor*) 7.16 (d, J = 8.1 Hz, 1H, *major*), 2.56 (s, 3H, *major*), 2.47 (s, overlap, 3H, *major*, 6H, *minor*); ^{13}CNMR ($CDCl_3$, 175 MHz): δ 142.7, 139.3, 130.0, 127.9 (q, J_{CF} = 28.6 Hz), 127.6, 124.3 (q, J_{CF} = 7.5 Hz), 124.0 (q, J_{CF} = 273.2 Hz), 23.9, 20.2 (q, J_{CF} = 2.0 Hz); ^{19}FNMR ($CDCl_3$, 465 MHz): δ -61.59 (*major*), -63.61 (*minor*); IR (neat): 2972, 1385, 1307, 1180, 1121; HRMS (EI) *m/z* calculated for $C_9H_9BrF_3$ ($[M+H]^+$) 251.9761, found 251.9765.

***tert*-butyl 5-bromo-2-(trifluoromethyl)-1H-pyrrolo[2,3-b]pyridine-1-carboxylate (13)**

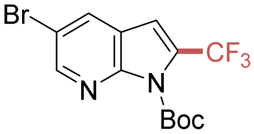

The reaction was run according to **Procedure 2** and purified via preparative TLC (5% EtOAc in hexanes) to yield the title compound (106 mg, 36%) as a white solid. ^{1}HNMR ($CDCl_3$, 500 MHz): δ 8.67 (d, J = 2.2 Hz, 1H), 8.11 (d, J = 2.2 Hz, 1H), 7.01 (s, 1H), 1.67 (s, 9H); ^{13}CNMR ($CDCl_3$, 175 MHz): δ 148.6, 147.7, 146.3, 132.6, 128.4 (q, J_{CF} = 39.9 Hz), 120.0 (q, J_{CF} = 268.6 Hz), 120.5, 115.2, 108.4(q, J_{CF} = 5.1 Hz), 86.6, 27.6; ^{19}FNMR ($CDCl_3$ 376 MHz): δ -59.77; IR (neat): 2337, 2361, 1761, 1394, 1122, 844; HRMS (ESI+) *m/z* calculated for $C_{13}H_{12}BrF_3N_2O_2Na$ ($[M+Na]^+$) 386.9927, found 386.9926.

3,3,3-trifluoro-1-phenylpropan-1-ol[8] (14)

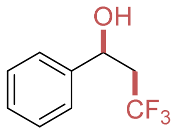

The reaction was run according to **Procedure 2** but with CH_2Cl_2 as the solvent. After 15 hours, the reaction was removed from the light source and diluted with 1.0 ml of MeOH. The reaction was stirred for an additional 60 minutes before adding 100 µl of 4N KOH solution and was then stirred for an additional 10 minutes. The reaction was then concentrated and purified on SiO_2 (100% hexanes to 5% EtOAc in hexanes) to provide the title compound (70 mg, 46%) as a colorless oil. The acquired ^{1}HNMR spectrum was consistent with that reported in the literature; ^{1}HNMR ($CDCl_3$, 400MHz): δ 7.41-7.31 (m, overlap, 5H), 5.10 (ddd, J = 9.6, 3.6, 3.2 Hz, 1H), 2.64 (tdd, J = 19.2, 15.2, 9.6 Hz), 2.46 (tdd, J = 19.2, 10.8, 3.6 Hz, 1H), 2.09 (d, J = 3.2 Hz, 1H).

(*E*)-4-(3,3,3-trifluoroprop-1-en-1-yl)benzonitrile[9] (15)

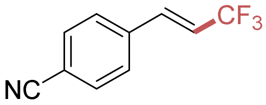

The reaction was run according to **Procedure 2** but with CH_2Cl_2 as the solvent. The following work up procedure to obtain **3** is un-optimized. After 15 hours, TFAA (110 µl, 1.0 equiv) was added to remove adventitious water, and the reaction was cooled to 0 °C. DBU (360 µl, 3.0 equiv) was added dropwise to the reaction to avoid reflux. Upon complete addition, the reaction was stirred for an additional 10 minutes before concentrating and purifying on SiO_2 (1% EtOAc in hexanes) to provide the title compound (90 mg, 56%) as a white solid. The acquired ^{1}HNMR spectrum was consistent with that reported in the literature; ^{1}HNMR ($CDCl_3$, 400MHz): δ 7.70 (d, J = 8.4 Hz, 2H), 7.56 ref. 12 (d, J = 8.4 Hz, 2H), 7.17 (dq, J = 16.3, 2.2 Hz, 1H), 6.31 (dq, J = 16.3, 6.4 Hz, 1H).

1,3-dimethyl-3-(2,2,2-trifluoroethyl)indolin-2-one[10] (16)

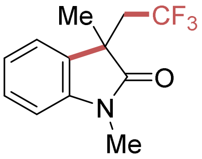

Procedure 1: 47% (FNMR)
Procedure 2: 53% (FNMR)
The reaction was run according to **Procedure 2** and was purified by preparative TLC (25% EtOAc in hexaes) to afford the title compound (78 mg, 40%) along with recovered starting material (20 mg, 14%). The acquired ^{1}HNMR spectrum was consistent with that reported in the literature; ^{1}HNMR ($CDCl_3$, 500MHz): δ 7.31 (dd, J = 7.8 Hz, 1H), 7.26 (d, 1H), 7.09 (dd, J = 7.8 Hz, 1H), 6.88 (d, J = 7.8 Hz, 1H), 3.24 (s, 3H), 2.82 (dq, J = 15.2, 10.7 Hz, 1H), 2.64 (dq, J = 15.2, 10.7 Hz, 1H), 1.41.

1,3,5-trimethyl-2-(trifluoromethyl)benzene[11] and 1,3,5-trimethyl-2,4-bis(trifluoromethyl)benzene[12] (17)

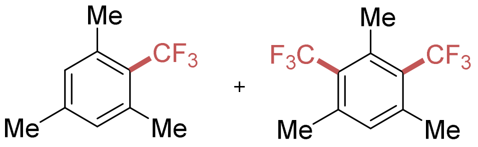

Procedure 1: 48% mono, 4% di (FNMR)
Procedure 2: 59% mono, 10% di (FNMR)
The reaction was run according to **Procedure 1** but using three equivalents of pyridine *N*-oxide (230 mg, 2.4 mmol) and 3.1 equivalents of TFAA (350 µl, 520 mg, 2.50 mmol). This was to ensure consumption of starting material, as its separation from the products is challenging. Upon completion of the reaction (49% mono, 20% di, ^{19}F NMR), the crude reaction was filtered through silica, and the resulting filtrate was concentrated to yield the title compounds (3:1, mono:di, 71 mg, 43%) as a clear oil. Further separation of the two products can be accomplished by preparative TLC (100% hexanes) to afford each product. The acquired ^{1}HNMR spectra were identical to those reported in the literature.

1,3,5-trimethyl-2-(trifluoromethyl)benzene: ^{1}HNMR ($CDCl_3$, 500 MHz): δ 6.89 (s, 2H), 2.44 (q, J_{CF} = 3.3 Hz), 2.29 (s, 3H)

1,3,5-trimethyl-2,4-bis(trifluoromethyl)benzene: ^{1}HNMR ($CDCl_3$, 500 MHz): δ 6.98 (s, 1H), 2.52 (spt, J_{HF} = 2.8 Hz, 3H), 2.47 (q, J_{HF} = 3.9 Hz, 6H)

1-methyl-3-(trifluoromethyl)pyridine-2(1*H*)-one[13] (18)

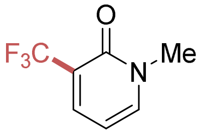

Procedure 1: 44% yield (FNMR)
Procedure 2: 41% yield (FNMR)
The reaction was run according to **Procedure 1** and was purified by column chromatography (0-40% EtOAc in hexanes) to afford a tan solid (57 mg, 40% yield). The acquired ^{1}HNMR spectrum was identical to that reported in the literature; ^{1}HNMR ($CDCl_3$, 500 MHz): δ 7.75 (d, *J* = 7.3 Hz, 1H), 7.50 (d, *J* = 7.3 Hz, 1H), 6.22 (dd, *J* = 7.3, 7.3 Hz);

1,3,7-trimethyl-8-(trifluoromethyl)-3,7-dihydro-1*H*-purine-2,6-dione: [14] **(19)**

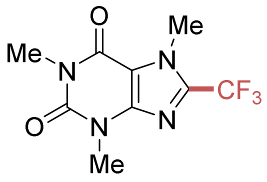

Procedure 1: 23% yield (FNMR)
Procedure 2: 41% yield (FNMR)
Procedure 3: 57% yield (FNMR)

The reaction was run according to **Procedure 3** except that due to low solubility the MeCN volume was raised to 4 ml. The substrate went into solution slowly over time. The reaction was purified on SiO_2 (10% EtOAc in hexanes) to afford the title compound (112.8 mg, 54%) as a white solid. The acquired ^{1}HNMR spectrum was identical to that reported in the literature; ^{1}HNMR ($CDCl_3$, 500 MHz): δ 4.16 (s, 3H), 3.59 (s, 3H), 3.42 (s, 3H);

***tert*-butyl 2-(trifluoromethyl)-1*H*-pyrrole-1-carboxylate and *tert*-butyl 2,5-bis(trifluoromethyl)-1*H*-pyrrole-1-carboxylate: (20)**

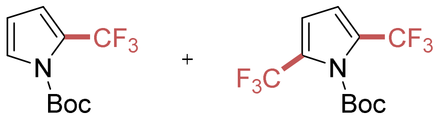

Procedure 1: 59% yield (FNMR) 55% mono, 4% bis, 15% starting material recovered
Procedure 2: 73% yield (FNMR) 37% mono, 36% bis
The reaction was run according to **Procedure 2** and was concentrated before running through a plug of silica gel with 100% DCM to decolorize the material. Concentration of the resulting filtrate provided the title compounds in a 1:1 mixture as a clear oil (160 mg, 74% yield). Further separation can be accomplished by preparative TLC (15% CH_2Cl_2 in hexanes) to afford the pure products. Products adopt a faint yellow hue upon further handling;

***tert*-butyl 2-(trifluoromethyl)-1*H*-pyrrole-1-carboxylate**: ^{1}HNMR ($CDCl_3$, 700 MHz): δ 7.44 (dd, J = 3.3, 2.2 Hz, 1H), 6.74 (br apparent t, J = 2.2 Hz, 1H), 6.19 (dd, J = 3.4, 3.3 Hz, 1H); ^{13}CNMR ($CDCl_3$, 175 MHz): δ 147.4, 125.8, 121.7 (q, J_{CF} = 40.2 Hz), 120.5 (q, J_{CF} = 265.7), 117.8 (q, J_{CF} = 4.8 Hz), 109.6, 85.6, 27.7; ^{19}FNMR ($CDCl_3$ 470 MHz): δ -59.29; IR (neat): 3151, 2986, 2938, 1752, 1305, 1320, 1284, 1124; HRMS (EI) *m/z* calculated for $C_{10}H_{13}F_3NO_2$ ($[M+H]^+$) 235.0820, found 235.0813.

***tert*-butyl 2,5-bis(trifluoromethyl)-1*H*-pyrrole-1-carboxylate**: ^{1}HNMR ($CDCl_3$, 700 MHz): δ 6.71 (s, 2H), 1.62 (s, 9H); ^{13}CNMR ($CDCl_3$, 175 MHz): δ 146.0, 126.3 (q, J_{CF} = 41.6 Hz), 119.8 (q, J_{CF} = 119.8 Hz), 114.4 (q, J_{CF} = 4.1 Hz), 87.9, 27.3; ^{19}FNMR ($CDCl_3$, 470 MHz): δ -59.36; IR (neat): 3151, 2988, 2933, 1778, 1296, 1257, 1133; HRMS (EI) *m/z* calculated for $C_6H_4F_3N$ ($[M\text{-}Boc+H]^+$) 203.0170, found 203.0170.

(5-(trifluoromethyl)thiophen-2-yl)boronic acid MIDA ester: (21)

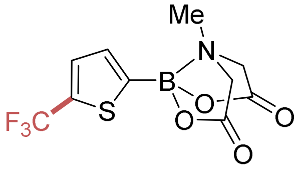

Procedure 1: 48% yield (FNMR)
Procedure 2: 64% yield (FNMR)
The reaction was run according to **Procedure 2** and was purified by column chromatography (20% MeCN in CH_2Cl_2) to afford a pink amorphous solid (~85% pure). This material was dissolved in a minimal volume of CH_2Cl_2 and diluted with diethyl ether. The product crashed out as a white suspension while the impurity (pink) oiled out on the bottom and sides of the vessel. The product slurry was decanted to provide the title compound in greater than 95% purity as a light pink amorphous solid (130 mg, 53%); ^{1}HNMR (CD_3CN, 400 MHz): δ 7.63-760 (m, 1H), 7.32-7.31 (m, 1H), 4.12 (d, *J* = 17.1, 2H), 3.96 (d, *J* = 17.1, 2H), 2.66 (s, 3H) ^{13}CNMR (CD_3CN, 175 MHz): δ 169.2, 135.3 (q, J_{CF} = 37.6 Hz), 134.8, 132.0 (q, J_{CF} = 3.3), 124.3 (q, J_{CF} = 267.7 Hz), 63.1, 49.0; ^{19}FNMR (CD_3CN 377 MHz): δ -55.53; IR (neat): 3015, 1750, 1541, 1452, 1332, 1286, 1144, 1108, 1033, 1003, 817 ; HRMS (EI) *m/z* calculated for $C_{10}H_{13}BF_3N_2O_4S$ ($[M+NH_4]^+$) 325.0637, found 325.0636.

(1-tosyl-5-(trifluoromethyl)-1*H*-pyrrol-2-yl)boronic acid MIDA ester: (22)

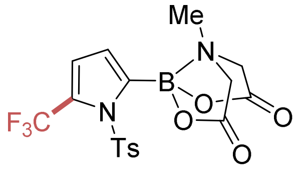

Procedure 1: 47% yield (FNMR)
Procedure 2: 57% yield (FNMR)
The reaction was run according to **Procedure 2** and was purified by filtration (20% MeCN in CH_2Cl_2) to afford a yellow amorphous solid (~85-90% pure). This material could be further purified by column chromatography (0-10% MeCN in DCM) to provide the title compound (164 mg, 46%) as a white solid: ^{1}HNMR (MeCN-*d*$_3$, 700 MHz): 7.72 (d, *J* = 8.3 Hz, 2H), 7.39 (d, *J* = 8.3 Hz, 2H), 7.02 (d, *J* = 3.7 Hz, 1H), 6.78 (d, *J* = 3.7 Hz, 1H), 4.08 (d, *J* = 17.6 Hz, 2H), 4.04 (d, *J* = 17.6 Hz, 2H), 2.88 (s, 3H), 2.41 (s, 3H); ^{13}CNMR (MeCN-*d*$_3$, 175 MHz): 169.6, 147.3, 137.4, 131.2, 129.2 (q, J_{CF} = 40.2 Hz), 127.9, 125.5, 121.7 (q, J_{CF} = 267.7 Hz), 120.9 (q, J_{CF} = 4.8 Hz), 66.0, 50.9, 22.0; ^{19}FNMR ($CDCl_3$, 470 MHz): -56.74; IR (neat): 2968, 1768, 1300, 1178, 1123, 1031; HRMS (ESI) *m/z* calculated for $C_{17}H_{17}BF_3N_2O_6S$ ($[M+H]^+$) 445.0847, found 445.0850.

Scale-up procedure for Substrates 17-20, and 13 and 21

General Procedure 4:
To a 500 mL recovery flask was added Ru(bpy)$_3$Cl$_2$•6H$_2$O (0.1 mol%), pyridine *N*-oxide (1.0, 2.0, 3.0, or 4.0 equiv.), MeCN, (2.5 mL/mmol substrate, standard reagent grade), the substrate (1.0 equiv), and the bright red homogenous solution was stirred for 2 minutes (Note the dissolution of pyridine *N*-oxide in MeCN is endothermic). Trifluoroacetic anhydride (1.1, 2.1, 3.1, or 4.1 equiv.) was added over 1 minute with no observable exotherm above 30 ˚C. The flask was placed within a 1L-jacketed beaker containing ^{i}PrOH, wrapped in a single 72W blue LED light strip and connected to a recirculating chiller set to 20 ˚C. The LED strip was turned on and after approximately 15 minutes the internal reaction temperature had reached a consistent 25-30 ˚C. After the requisite period of time the reaction mixture was worked up and purified as stated. Typically the reactions darken over time which did not represent a significant problem until the reaction was conducted on 100 g scale (**See section 10**)
Note: The LED strip becomes hot during operation and thus the jacketed beaker was employed to regulate the temperature of the reaction. This set up is un-optimized and was used for its ease of operation.

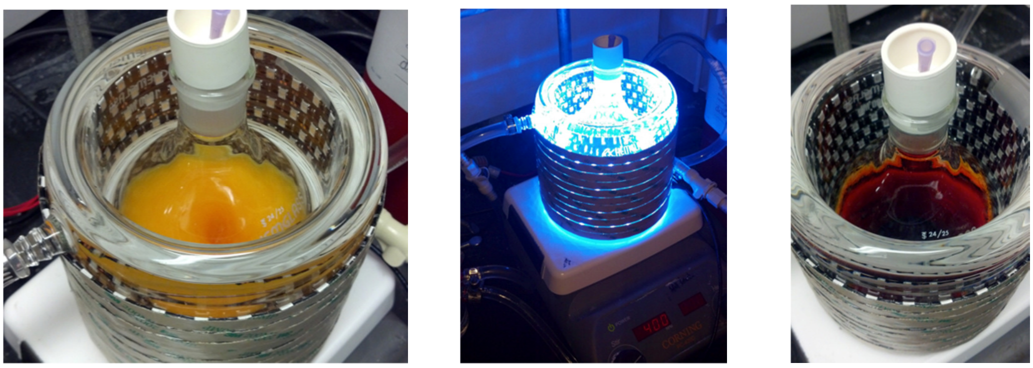

(**a**) (**b**) (**c**)

Representative reaction set up shown for Caffeine which was the only heterogeneous mixture at the outset of the reaction (**a**). The 72 W LED has significant brightness and we typically shielded the set up with aluminum foil to protect our eyes (**b**). Typically the solution darkens over the course of the reaction (**c**).

1,3,5-trimethyl-2-(trifluoromethyl)benzene and 1,3,5-trimethyl-2,4-bis(trifluoromethyl)benzene (17)

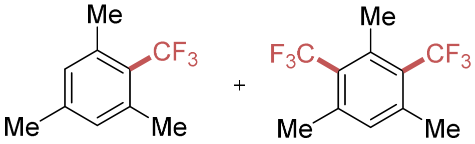

The reaction was run according to **Procedure 4.** $Ru(bpy)_3Cl_2$•$6H_2O$ (31.1 mg, 0.042 mmol, 0.1 mol%), pyridine *N*-oxide (11.9 g, 125 mmol, 3.0 equiv), MeCN (100 mL), mesitylene (5.00 g, 41.6 mmol, 1.0 equiv), and TFAA (27.1 g, 130 mmol, 3.1 equiv). After 46 h the crude dark red/black reaction mixture was concentrated to a black oil, diluted with hexane (20 mL) and filtered through a silica column (6 inch x 1.5 inch) eluting with hexanes. The colorless hexanes solution (~400 mL) was dried with Na_2SO_4 then filtered and concentrated (40 ˚C, 150 mbar) to yield the title compounds (4.80 g, 4:1 mono:di, 58%) as a clear colorless oil in accordance with the spectroscopic details previously reported (*vide supra*).

1-methyl-3-(trifluoromethyl)pyridine-2(1*H*)-one (18)

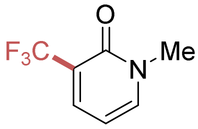

The reaction was run according to **Procedure 4.** $Ru(bpy)_3Cl_2$•$6H_2O$ (34.3 mg, 0.046 mmol, 0.1 mol%), pyridine *N*-oxide (4.36 g, 45.8 mmol, 1.0 equiv), MeCN (115 mL), 1-methyl-2-pyridone (5.00 g, 45.8 mmol, 1.0 equiv), and TFAA (10.6 g, 50.4 mmol, 1.1 equiv). After 16.5 h the crude dark red reaction mixture (35% yield by ^{19}F NMR) was diluted with CH_2Cl_2 (150 mL) then washed with 1N HCl (200 mL), sat $NaHCO_3$ (200 mL), dried with Na_2SO_4 then filtered and concentrated to yield a crude dark brown/black solid. The crude material was purified via column chromatography (50% EtAOc:Hexanes) to yield the title compound (2.56 g, 32%) as a light pink solid in accordance with the spectroscopic details previously reported (*vide supra*).

1,3,7-trimethyl-8-(trifluoromethyl)-3,7-dihydro-1*H*-purine-2,6-dione (19)

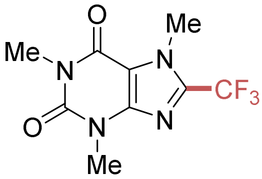

To a 500 mL recovery flask was added $Ru(bpy)_3Cl_2$•$6H_2O$ (19.3 mg, 0.026 mmol, 0.1 mol%), pyridine *N*-oxide (9.79 g, 103.0 mmol, 4.0 equiv), MeCN (250 mL), caffeine (5.0 g, 25.75 mmol, 1.0 equiv), and the bright red heterogeneous solution was stirred for 2 minutes (Note the dissolution of pyridine *N*-oxide in MeCN is endothermic and the solubility of caffeine in MeCN is low). TFAA (43.26 g, 206 mmol, 8.0 equiv) was added and the flask was placed within a 1L-jacketed beaker containing ^{i}PrOH, wrapped in a single 72W blue LED light strip and connected to a recirculating heater/chiller set to 20 ˚C. The LED strip was turned on and after approximately 15 minutes the internal reaction temperature had reached a consistent 25-30 ˚C. After 20 h the crude dark red reaction mixture (61% yield by ^{19}F NMR) was concentrated to remove the MeCN, diluted with EtOAc (200 mL) then washed with water (200 mL), sat $NaHCO_3$ (200 mL), and water (200 mL). The brown solution was dried with Na_2SO_4 then filtered and concentrated to yield a crude brown solid that was chromatographed on SiO2 (5% EtOAc to 10% EtOAc in hexanes) to yield the title compound (3.88 g, 58%) as a light brown solid in accordance with the spectroscopic details previously reported (*vide supra*).

***tert*-butyl 2-(trifluoromethyl)-1*H*-pyrrole-1-carboxylate and *tert*-butyl 2,5-bis(trifluoromethyl)-1*H*-pyrrole-1-carboxylate (20)**

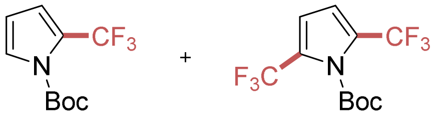

The reaction was run according to **Procedure 4.** $Ru(bpy)_3Cl_2•6H_2O$ (22.4 mg, 0.030 mmol, 0.1 mol%), pyridine *N*-oxide (5.69 g, 59.8 mmol, 2.0 equiv), MeCN (100 mL), *N*-Boc-pyrrole (5.00 g, 29.9 mmol, 1.0 equiv), and TFAA (13.2 g, 62.8 mmol, 2.1 equiv). After 15 h the crude dark red reaction mixture (50% yield mono 13% di by ^{19}F NMR) was diluted with CH_2Cl_2 (150 mL) then washed with 1N HCl (200 mL), sat $NaHCO_3$ (200 mL), dried with Na_2SO_4 then filtered and concentrated to yield a crude dark brown oil. This was diluted with hexane (20 mL) and filtered through a silica column (6 inch x 1.5 inch) eluting with hexanes. The colorless hexanes solution (~400 mL) was dried with Na_2SO_4 then filtered and concentrated to yield the title compound (4.50 g, 4:1 mono:di, 61%) as a yellow oil in accordance with the spectroscopic details previously reported (*vide supra*). The yellow oil could be further purified to remove the color via Kugelrohr distillation (75–80 ˚C, 1 mbar) to provide (4.02 g, 4:1 mono:di, 53%).

(5-(trifluoromethyl)thiophen-2-yl)boronic acid MIDA ester (21)

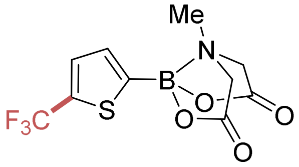

The reaction was run according to **Procedure 4** but using a 100 mL round-bottom-flask. $Ru(bpy)_3Cl_2•6H_2O$ (15.8 mg, 0.021 mmol, 0.1 mol%), pyridine *N*-oxide (4.01 g, 42.2 mmol, 2.0 equiv), MeCN (55 mL), thiophen-2-yl boronic acid MIDA ester (5.04 g, 21.1 mmol, 1.0 equiv), and TFAA (9.30 g, 44.3 mmol, 2.1 equiv). After 15 h the crude dark pink reaction mixture (57% yield by ^{19}F NMR) was concentrated to ~10 mL then diluted with CH_2Cl_2 (150 mL) then washed with 1N HCl (100 mL), twice with sat $NaHCO_3$ (100 mL), dried with Na_2SO_4 then filtered and concentrated to yield a crude pink foam. The crude material was purified via column chromatography (5% MeCN:CH_2Cl_2 to 10% MeCN:CH_2Cl_2) to yield the title compound (2.52 g, 39%) as a white solid in accordance with the spectroscopic details previously reported (*vide supra*). Significant material is lost during column chromatography as the SM and product have very similar RF.

***tert*-butyl 5-bromo-2-(trifluoromethyl)-1H-pyrrolo[2,3-b]pyridine-1-carboxylate (13)**

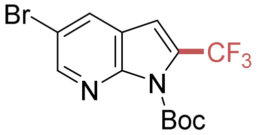

The reaction was run according to **Procedure 4** but using a 100 mL round-bottom-flask. $Ru(bpy)_3Cl_2•6H_2O$ (15.8 mg, 0.017 mmol, 0.1 mol%), pyridine *N*-oxide (3.20 g, 33.6 mmol, 2.0 equiv), MeCN (55 mL), *tert*-butyl 5-bromo-2-(trifluoromethyl)-1H-indole-1-carboxylate (5.00 g, 16.8 mmol, 1.0 equiv), and TFAA (7.42 g, 35.3 mmol, 2.1 equiv). After 15 h the crude dark red reaction mixture (34% yield by ^{19}F NMR) was diluted with CH_2Cl_2 (100 mL) then washed with 1N HCl (100 mL), sat $NaHCO_3$ (100 mL), dried with Na_2SO_4 then filtered and concentrated to yield a crude solid. The crude material was purified via column chromatography (0% to 5% Hexane:EtAOc) then recrystallized from hexanes to yield the title compound (1.68 g, 27%) as a white solid in accordance with the spectroscopic details previously reported (*vide supra*).

Cross-coupling of 13 and 21

***tert*-butyl 2-(trifluoromethyl)-5-(5-(trifluoromethyl)thiophen-2-yl)-1*H*-indole-1-carboxylate (23)**

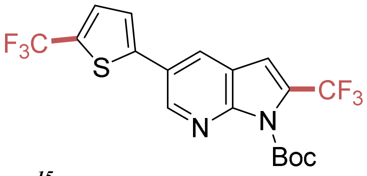

Following the procedure of Burke *et al.*[15] To a 25 mL RBF with a stir bar was added *tert*-butyl 5-bromo-2-(trifluoromethyl)-1H-pyrrolo[2,3-b]pyridine-1-carboxylate (297 mg, 1.0 mmol, 1.0 equiv), (5-(trifluoromethyl)thiophen-2-yl)boronic acid MIDA ester (368 mg, 1.2 mmol, 1.2 equiv), SPhos (41.1 mg, 0.1 mmol, 0.1 equiv), $Pd(OAc)_2$ (11.2 mg, 0.005 mmol, 0.05 equiv) and the flask was evacuated and backfilled with nitrogen 3 times. 1,4-Dioxane (55 mL) was added and the solution stirred for 10 minutess followed by the addition of K_2PO_4 (2.5 mL 3M (aq) and sparged for 30 mins with N_2) and the mixture was heated to 60 ˚C for 4.5 h. The dark brown solution was cooled to rt, added to a separatory funnel, diluted with 1N NaOH (10 mL) and extracted with Et_2O (3 × 25 mL). The combined organics were dried with Na_2SO_4 then filtered through celite and concentrated to yield a crude brown solid. The crude material was purified via column chromatography (5% Hexane:EtAOc) to yield the title compound (274 mg, 63%) as a white solid.

^{1}HNMR ($CDCl_3$, 700 MHz): δ 8.88 (d, J = 2.2 Hz, 1H), 8.13 (d, J = 2.2 Hz, 1H), 7.44 (d, J = 2.9 Hz 1H), 7.28 (d, J = 2.9 Hz 1H), 7.10 (s, 1H), 1.68 (s, 9H); ^{13}CNMR ($CDCl_3$, 175 MHz): δ 149.4, 146.7, 146.0, 144.6, 131.2 (q, J_{CF} = 38.7 Hz), 129.8 (q, J_{CF} = 3.8 Hz), 128.6 (q, J_{CF} = 39.8 Hz), 128.1, 125.5, 123.9, 122.3 (q, J_{CF} = 268.7 Hz), 120.4 (q, J_{CF} = 268.5 Hz), 119.3, 109.4 (q, J_{CF} = 4.9 Hz), 86.8, 27.8 ^{19}FNMR ($CDCl_3$ 376 MHz): δ -55.50, -58.71; IR (neat): 1761, 1397, 1306, 1286, 1231, 1139, 1122, 1107, 842, 802; HRMS (ESI+) *m/z* calculated for $C_{19}H_{1g}F_6NO_2S$ ($[M+H]^+$), 437.0753 found 437.0757.

Halogenation/Demethylation of 6

methyl 6-bromo-5-(trifluoromethyl)nicotinate (25)

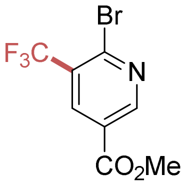

This reaction is un-optimized. To a 15 mL sealed pressure tube with a stir bar was added methyl 1-methyl-6-oxo-5-(trifluoromethyl)-1,6-dihydropyridine-3-carboxylate (100 mg, 0.43 mmol, 1.0 equiv), $POBr_3$ (610 mg, 2.13 mmol, 5.0 equiv) and PBr_3 (80.8 μL, 0.85 mmol, 2.0 equiv). The tube was flushed with N_2 then sealed and heated to 120 °C for 24 h. The black solution was cooled in an ice/water bath then cautiously quenched with ice cold water (5 mL over 5 min, patience is advised as there is typically a delay before addition of water causes a violent quenching of the excess reagents) followed by conc. NH_4OH (3 mL). The heterogeneous solution was diluted with water (50 mL) and CH_2Cl_2 (50 mL) in a beaker then transferred to a separatory funnel. The layers were separated, the aqueous layer extracted with CH_2Cl_2 (50 mL), and the combined organics were dried with Na_2SO_4 then filtered and concentrated to yield a light brown solid. The crude material was purified via column chromatography (0% to 5% Hexane:EtAOc) to yield the title compound (77 mg, 64%) as a white solid.

^{1}HNMR ($CDCl_3$, 400 MHz): δ 9.09 (d, J = 1.8 Hz, 1H), 8.53 (d, J = 1.8 Hz, 1H), 4.00 (s, 3H); ^{13}CNMR ($CDCl_3$, 175 MHz): δ 164.0, 153.3, 144.1, 137.3, (q, J_{CF} = 5.0 Hz), 128.1 (q, J_{CF} = 33.8 Hz), 125.3, 122.0 (q, J_{CF} = 273.2 Hz), 53.2 ^{19}FNMR ($CDCl_3$ 376 MHz): δ -63.77; IR (neat): 3064, 1727, 1595, 1568, 1432, 1407, 1250, 1129, 1047, 963, 770; HRMS (APCI) *m/z* calculated for $C_8H_6BrF_3NO_2$ ($[M+H]^+$), 283.9526 found 283.9529.

methyl 6-chloro-5-(trifluoromethyl)nicotinate:[16] **(24)**

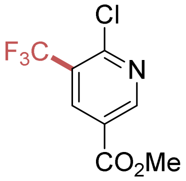

This reaction is un-optimized. To a 15 mL sealed pressure tube with a stir bar was added methyl 1-methyl-6-oxo-5-(trifluoromethyl)-1,6-dihydropyridine-3-carboxylate (100 mg, 0.43 mmol, 1.0 equiv), $POCl_3$ (396 μL, 4.25 mmol, 10.0 equiv) and PCl_5 (177 mg, 0.85 mmol, 2.0 equiv). The tube was flushed with N_2 then sealed and heated to 120 °C for 24 h. The black solution was cooled in an ice/water bath then cautiously quenched with ice cold water (5 mL over 5 min) followed by conc. NH_4OH (3 mL). The heterogeneous solution was diluted with water (50 mL) and CH_2Cl_2 (50 mL) in a beaker then transferred to a seporatory funnel. The layers were separated and the aqueous layer was extracted with CH_2Cl_2 (50 mL) and the combined organics were dried with Na_2SO_4 then filtered and concentrated to yield a light yellow oil that solidified on standing. The crude material was purified via column chromatography (0% to 5% Hexane:EtAOc to yield (48 mg, 47%) as a white solid.

The acquired ^{1}HNMR spectrum was consistent with that reported in the literature; ^{1}HNMR ($CDCl_3$, 500MHz): δ 9.14 (d, J = 2.0 Hz, 1H), 8.60 (d, J = 2.0 Hz, 1H), 4.00 (s, 3H).

Trifluoromethylation of *N*-Boc-pyrrole (18.4 gram scale)

***tert*-butyl 2-(trifluoromethyl)-1*H*-pyrrole-1-carboxylate and *tert*-butyl 2,5-bis(trifluoromethyl)-1*H*-pyrrole-1-carboxylate (20)**

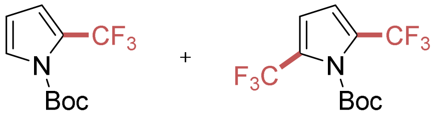

The reaction was run according to **Procedure 3.** $Ru(bpy)_3Cl_2$•$6H_2O$ (82.5 mg, 0.11 mmol, 0.1 mol%), pyridine *N*-oxide (21.0 g, 220 mmol, 2.0 equiv), MeCN (375 mL), *N*-Boc-pyrrole (18.4 g, 110 mmol, 1.0 equiv), and TFAA (48.6 g, 231 mmol, 2.1 equiv). After 15 h the crude dark red reaction mixture (59% yield mono 12% di by ^{19}F NMR) was diluted with CH_2Cl_2 (350 mL) then washed with 1N HCl (400 mL), sat $NaHCO_3$ (200 mL), dried with

Na_2SO_4 then filtered and concentrated to yield a crude dark brown oil. This was diluted with hexane (100 mL) and filtered through a silica column (6 inch x 1.5 inch) eluting with hexanes. The colorless hexanes solution (1000 mL) was dried with Na_2SO_4 then filtered and concentrated to yield the title compounds (15.8 g, 5:1 mono:di, 57%) as a colorless oil in accordance with the spectroscopic details previously reported (*vide supra*).

Trifluoromethylation of *N*-Boc-pyrrole (101 gram scale)
***tert*-butyl 2-(trifluoromethyl)-1*H*-pyrrole-1-carboxylate and *tert*-butyl 2,5-bis(trifluoromethyl)-1*H*-pyrrole-1-carboxylate (20)**

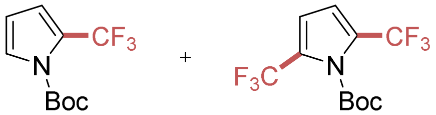

To a 3 neck 3L RBF fitted with an overhead stirrer and thermometer placed within a desiccator (See **picture below**), was added *N*-Boc-pyrrole (101 g, 605 mmol, 1.0 equiv), MeCN (1000 mL), $Ru(bpy)_3Cl_2•6H_2O$ (453 mg, 0.61 mmol, 0.1 mol%) and pyridine *N*-oxide (115 g, 1210 mmol, 2.0 equiv) washed in with MeCN (250 mL). This heterogeneous solution was stirred until the pyridine *N*-oxide had fully dissolved (~ 2 minutes) at which time the internal reaction temperature was 15 ˚C. TFAA (267 g, 1271 mmol, 2.1 equiv) was added portion-wise over 2 minutes with a concurrent exotherm to 35 ˚C. The bright red solution was irradiated with a single strip of 72 W (total power) LEDs that were air cooled from a flow or air within the desiccator, with the internal reaction temperature consistent at 35 ˚C over the course of the reaction. The homogenous solution was stirred for 62 h after which it was deemed by ^{19}F NMR to have stalled at 33% yield mono, 2% yield di (13:1 mono:di). As the reaction becomes increasingly dark over time we attribute the reduced reactivity to a factor of light penetration, the same total light intensity being used for 20 g and 100 g scale. Attempts to separate the residual *N*-Boc-pyrrole from the product via repeated distillation were unsuccessful and thus no further purification was undertaken. Research is underway to establish the cause of the solution darkening over time as well as further studies on scale employing a stronger (higher W) light source with a more optimized reaction set up.

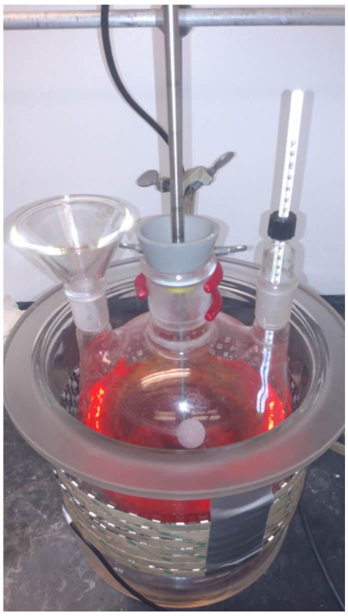

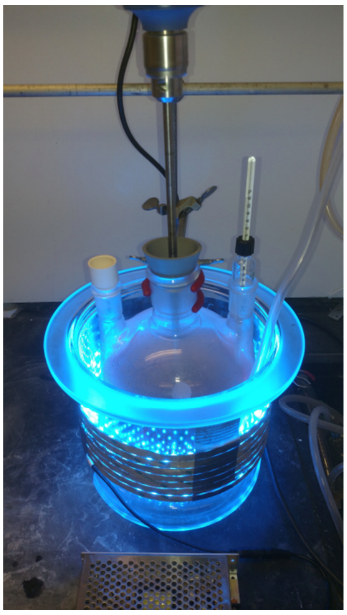

100 g batch reaction set up.

Reaction in continuous flow

***tert*-butyl 2-(trifluoromethyl)-1*H*-pyrrole-1-carboxylate and *tert*-butyl 2,5-bis(trifluoromethyl)-1*H*-pyrrole-1-carboxylate (20)**

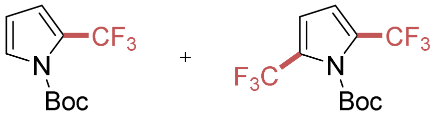

The following set up and conditions are un-optimized and serve as a proof of concept as to the ability to perform this reaction at steady state conditions in flow. No direct comparisons between batch and flow have been made at this time, as neither process is fully optimized or demonstrated on a wide selection of substrates.

A flow trifluoromethylation of 20 grams of *N*-Boc-pyrrole was performed using the Vapourtec® E-series integrated flow chemistry system equipped with a UV-150 photochemical reactor (http://www.vapourtec.co.uk). This UV-150 reactor was equipped with a 450 nm light source (24W Radiant Output) and a dichroic mirror. The PFA reactor cartridge (part no. 50-1287) possessed a 10 ml internal volume, with 1.3 mm internal diameter and 0.15 mm wall thickness. For reliable steady-state elution times, a known length of tubing (28 cm, supplied by Vapourtec®) connected each pump to a static T mixer (purchased from Upchurch Scientific via the Idex website, https://www.idex-hs.com, part P-632) with another 28 cm section leading from the T mixer to the reactor (1/2 of this volume was attributed to each reagent stream for a total length of 42cm of tubing each before the reactor). A further 115 cm of tubing led from the outlet of the reactor to a collection valve (Vapourtec® collection valve kit). The back-pressure regulator was adjusted to supply 4 bar of back pressure at 1 ml min^{-1} flow rate. Solution A contained TFAA (37.25 ml, 263.7 mmol, 2.1 equiv) and was made up to a volume of 189 ml. Solution B contained $Ru(bpy)_3Cl_2•6H_2O$ (94.0 mg, 0.126 mmol, 0.1 mol%), pyridine *N*-oxide (23.89 g, 251.2 mmol, 2.0 equiv), *N*-Boc-pyrrole (21.00 g, 125.6 mmol, 1.0 equiv) and was made up to a total volume of 189 mL with MeCN. Each solution was pumped at a flow rate of 0.5 ml/min to result in a 1 ml/min flow rate after the T mixer. A total volume of 372 ml of solution (186 ml of each solution) was pumped over a span of 6 hours and 12 minutes, with a steady state collection of 360 minutes (360 ml of 0.33M solution collected). An analysis of the final reaction solution yielded 77% yield (FNMR vs. trifluorotoluene) of the final product in 46% yield mono, 31% yield di (3:2 mono:di). The collected solution was concentrated (minimum pressure 150 mbar at 40 ˚C), diluted with CH_2Cl_2 (400 ml), washed with 1N HCl (400 ml) and sat. $NaHCO_3$ (400 ml). The aqueous layer from the bicarbonate wash was extracted with an additional 200 ml of CH_2Cl_2, and the organics were combined, washed with brine, over Na_2SO_4 and filtered. The resulting solution was concentrated (minimum pressure 150 mbar at 40 ˚C) to a dark brown oil. This was diluted with hexane (200 ml) and filtered through a plug of silica (10 in. x 1.5 in.), followed by a wash with 300 ml of hexanes. The resulting solution was concentrated (minimum pressure 150 mbar at 40 ˚C) to yield the title product as a clear oil (23.1 g, 3:2 mono:di, 71% yield) in accordance with the spectroscopic details previously reported (*vide supra*).

Supplementary References:

[1] Ye, Y., Lee, S. H., & Sanford, M. S. Silver-mediated trifluoromethylation of arenes using $TMSCF_3$. *Org. Lett.* **13,** 5464–5467 (2011).

[2] Lishchynskyi, A.; Novikov, M. A.; Martin, E.; Escudero-Adán, E. C.; Novák, P.; Grushin, V. V. Trifluoromethylation of aryl and heteroaryl halides with fluoroform-derived $CuCF_3$. *J. Org. Chem.* **78,** 11126–11146 (2013).

[3] Khan, B. A.; Buba, A. E.; Gooβen, L. J. Oxidative trifluoromethylation of arylboronates with shelf-stable potassium (trifluoromethyl)trimethoxyborate. *Chem. Eur. J.* **18,** 1577–1581 (2012).

[4] Danoun, G.; Bayarmagnai, B.; Grünberg, M. F.; Gooβen, L. J. Sandmeyer Trifluoromethylation of Arenediazonium Tetrafluoroborates. *Angew. Chem., Int. Ed.* **52,** 7972–7975 (2013).

[5] Sladojevich, F.; McNeill, E.; Börgel, J.; Zheng, S.-L.; Ritter, T. Condensed-phase, halogen-bonded CF_3I and C_2F_5I adducts for perfluoroalkylation reactions. *Angew. Chem., Int. Ed.* **54,** 3712–3716 (2015).

[6] Liu, T.; Shen, Q. Copper-catalyzed trifluoromethylation of aryl and vinyl boronic acids with an electrophilic trifluoromethylating reagent. *Org. Lett.* **13,** 2342–2345 (2011).

[7] Nagib, D. A.; MacMillan, D. W. C. Trifluoromethylation of arenes and heteroarenes by means of photoredox catalysis. *Nature*, **480,** 224–228 (2011).

[8] Li, Y.; Studer, A. Transition-metal-free trifluoromethylaminoxylation of alkenes. *Angew. Chem., Int. Ed.* **51,** 8221–8224 (2012).

[9] Yasu, Y.; Koike, T.; Akita, M. Visible-light-induced synthesis of a variety of trifluoromethylated alkenes from potassium vinyltrifluoroborates by photoredox catalysis. *Chem. Commun.* **49,** 2037–2039 (2013).

[10] Li, L.; Deng, M.; Zheng, S. C.; Xiong, Y. P.; Tan, B.; Liu, X. Y. Metal-free direct intramolecular carbotrifluoromethylation of alkenes to functionalized trifluoromethyl azaheterocycles. *Org. Lett.* **16,** 504–507 (2014).

[11] Li, Y.; Chen, T.; Wang, H.; Zhang, R.; Jin, K.; Wang, X.; Duan, C. A ligand-free copper-catalyzed decarboxylative trifluoromethylation of aryliodides with sodium trifluoroacetate using Ag_2O as a promoter. *Synlett* **12,** 1713–1716 (2011).

[12] Kino, T.; Nagase, Y.; Ohtsuka, Y.; Yamamoto, K.; Uragachi, D.; Tokuhisa, K.; Yamakawa, T. Trifluoromethylation of various aromatic compounds by CF_3I in the presence of Fe(II) compound, H_2O_2 and dimethylsulfoxide. *J. Fluorine Chem.* **131,** 98–105 (2010).

[13] Cui, L.; Matusaki, Y.; Tada, N.; Miura, T.; Uno, B.; Itoh, A. Metal-free direct C–H perfluoroalkylation of arenes and heteroarenes using a photoredox organocatalyst. *Adv. Synth. Catal.* **355,** 2203–2207 (2013).

[14] Ji, Y.; Bruecki, T.; Baxter, R. D.; Fujiwara, Y.; Seiple, I. B.; Su, S.; Blackmond, D. G.; Baran, P. S. Innate C–H trifluoromethylation of heterocycles. *P. Natl. Acad. Sci. USA* **108,** 14411–14415 (2011).

[15] Knapp, D. M.; Gillis, E. P.; Burke, M. D. A general solution for unstable boronic acids: slow-release cross-coupling from air-stable MIDA boronates. *J. Am. Chem. Soc.* **131,** 6961–6963 (2009).

[16] Mulder, J. A.; Frutos, R. P.; Patel, N. D.; Qu, B.; Sun, X.; Tampone, T. G.; Gao, J.; Sarvestani, M.; Eriksson, M. C.; Haddad, N.; Shen, S.; Song, J. J.; Senanayake, C. H. Development of a safe and economical synthesis if methyl 6-chloro-5-(trifluoromethyl)nicotinate: trifluoromethylation on kilogram scale. *Org. Process Res. Dev.* **17,** 940–945 (2013).
